# Unlocking the Power of Metaproteomics: A Novel Tool for Enhancing Shorebird Conservation and Blue-Economy Management

**DOI:** 10.3390/ijms27219508

**Published:** 2026-10-24

**Authors:** Almudena Escobar-Niño, María Victoria Calcis-Marzán, Andrea Forján-Miguéns, Irene Punta-Sánchez, Olivier Coste, Alejandro Pérez-Hurtado, Francisco-Javier Fernández-Acero

**Affiliations:** 1 Microbiology and Proteomics Laboratory, Institute for Viticulture and Agri-Food Research (IVAGRO), Faculty of Environmental and Marine Sciences, Department of Biomedicine, Biotechnology and Public Health, University of Cádiz, 11510 Puerto Real, Spain; 2 Department of Biology, University of Cádiz, 11510 Puerto Real, Spainalejandro.perez@gm.uca.es (A.P.-H.); 3 Microbiology and Proteomics Laboratory, Institute for Viticulture and Agri-Food Research (IVAGRO), Department of Analytical Chemistry, Faculty of Science, University of Cadiz, 11510 Puerto Real, Spain

**Keywords:** metaproteomics, dietary analysis, Cadiz Bay Natural Park, wader conservation, blue economy

## Abstract

Metaproteomics, the large-scale analysis of proteins from environmental or host-associated samples, has emerged as a transformative tool for characterizing microbial communities and their functional roles. Unlike metagenomics or metatranscriptomics, metaproteomics directly captures phenotypic expression, linking molecular function to ecological and physiological outcomes. Beyond its established applications in human microbiome and dietary research, metaproteomics enables the precise identification of food-derived protein biomarkers, including the discrimination of tissue-specific origins. This level of resolution is not attainable through DNA-based approaches. Here, we applied metaproteomics for the first time to analyze the feces of shorebirds in the salt flats and salt marshes of Cadiz Bay, aiming to determine their diet with unprecedented resolution. Conventional methods, based on direct observation or fecal inspection, are labor-intensive and risk losing critical dietary information. By contrast, metaproteomics provides rapid, high-resolution insights, offering a robust alternative for ecological monitoring. Our results demonstrate the potential of this technique to transform natural area management, optimize conservation strategies for avian species, and support the sustainable development of the blue economy. This study highlights metaproteomics as an innovative, cross-sectoral approach capable of advancing both fundamental ecological research and practical conservation outcomes.

## 1. Introduction

Recent advances in analytical methodologies have significantly improved our ability to investigate complex systems across different scientific disciplines. In materials science, spectroscopic techniques have been applied to characterize functional materials with potential applications in energy storage, providing valuable insights into their physicochemical properties [1]. Similarly, studies investigating structural modifications and their effects on material properties have contributed to the development of approaches for modeling and optimizing complex systems [2]. These examples highlight the importance of advanced analytical strategies in addressing diverse scientific challenges. In biological sciences, the analysis of complex biological samples has also gained increasing attention, particularly in areas such as food monitoring, agriculture, and the agri-food industry. In this context, proteomics has emerged as a promising tool for identifying protein biomarkers and peptide profiles that enable advanced monitoring and characterization of biological systems [3,4]. For this type of approach, a rapidly growing field known as metaproteomics has gained attention. This new approach, also referred to as community proteomics or environmental proteomics is mainly applied with the aim of fully understanding microbial communities and their interaction with the environment, which requires not only identifying the microorganisms involved and their biodiversity, but also determining their metabolic functions at both the cellular and community levels [5]. Since its introduction in 2004, mass spectrometry-based metaproteomics has rapidly become a powerful tool for the functional characterization of a wide variety of microbial communities, establishing the direct links of molecular-level phenotypes within their specific environments [6]. Thus, metaproteomics complements other meta-omic approaches. Metagenomics and metatranscriptomics provide only exploratory insights into microbial diversity and functional potential, but cannot capture their actual phenotypes [7]. In recent years, the use of metaproteomics has expanded beyond its traditional use in human microbiomes. The study by Petrone and colleagues applied metaproteomics for the first time to human fecal samples to identify proteins directly derived from consumed foods. Notably, this approach enabled the discrimination of tissue or food matrix-specific origins (e.g., meat, egg, and dairy), a level of specificity that cannot be achieved using DNA-based analyses [8]. This new approach significantly expands metaproteomic scope in nutritional and human health research [8]. In the present study, metaproteomics was applied for the first time to analyze fecal samples from shorebirds in the salt flats and salt marshes of Cadiz Bay, with the aim of determining their diet.

Coastal wetlands are key habitats for migratory shorebirds. Food availability and quality in these systems play a central role in shaping habitat use, foraging strategies and population dynamics along different flyways [9,10,11]. Both natural intertidal habitats and anthropogenic wetlands, such as coastal saltpans, act as important feeding grounds during periods of high energetic demand [12,13]. For this reason, understanding how shorebirds exploit trophic resources within coastal wetlands is essential to support effective conservation and management strategies. Most dietary studies on shorebirds have relied on direct observation or on the analysis of prey remains in feces and pellets. These methods require a considerable investment of time and are affected by well-documented biases. Small prey items and prey lacking hard, digestion-resistant structures, such as soft-bodied invertebrates, are often underestimated. Differential digestibility among prey types can distort diet reconstructions. This problem has been clearly illustrated in classic studies of shorebird diets in coastal wetlands and saltpans based on pellet and dropping analyses [12,14]. Similar biases occur when prey remains are used as proxies for diet composition, leading to the systematic underrepresentation of soft-bodied prey and limiting ecological inference [15]. Evidence from saltpan-based studies further indicates that traditional methods may not fully capture prey diversity or size spectra exploited by shorebirds [13].

Alternative approaches, including stable isotope analysis and DNA-based dietary techniques, have improved the study of shorebird diets. Stable isotope analysis has been used to assess habitat use and dietary contributions in migratory shorebirds from coastal wetlands and saltpans. However, its taxonomic resolution is limited and sampling usually requires capturing individuals [9,11]. DNA metabarcoding of fecal samples has greatly increased dietary resolution. It allows the detection of a wide range of prey taxa and reveals trophic links previously overlooked in both adult birds and chicks [10,16,17]. Nevertheless, DNA-based approaches cannot discriminate among consumed tissues or provide direct information on prey functional relevance. In this methodological context, metaproteomics represents a complementary approach with strong potential. Unlike DNA metabarcoding, which is subject to amplification biases during PCR that can distort relative quantification, metaproteomics does not rely on PCR, thereby avoiding this bias. The application of metaproteomics will provide highly valuable results for the management of natural areas, offering greater efficiency in terms of time and minimizing data loss. This will open new pathways for shorebird ecology and conservation, as well as the promotion of the blue economy.

## 2. Results

### 2.1. Custom Database Construction

For the generation of the custom databases required for this study, two groups of proteomes were used: invertebrate proteomes and shorebird proteomes. The shorebird proteomes were selected according to the species from which the fecal samples were obtained: *Actitis hypoleucos*, *Calidris alba*, *Calidris alpina*, *Charadrius alexandrinus*, *Charadrius hiaticula*, *Himantopus himantopus*, *Recurvirostra avosetta*, *Sternula albifrons*, *Tringa nebularia*, and *Tringa tetanus.* Since proteomes were not available for all these shorebird species, the closest available relatives were selected, *Arenaria interpres*, *Calidris pygmaea*, *Charadrius vociferus*, *Himantopus himantopus*, and *Phaetusa simplex*, comprising a total of 81,995 proteins. This protein group was used to filter shorebird proteins present in the fecal samples, preventing them from being misidentified as invertebrate proteins.

Next, as the ultimate aim of the project is to elucidate whether a metaproteomic approach is useful in the determination of the invertebrate diet of these shorebirds, all taxonomic groups containing invertebrates were selected. The selected proteomes included proteins available in UniProt from the following clades or subphyla: Spiralia (4,234,181), Cephalochordata (137,062), Tunicata (81,963), and Ecdysozoa (11,622,156).

After the stepwise reduction of the shorebird database, a total of 3647 proteins remained. In the case of invertebrates, four protein databases were obtained: Cephalochordata with 2780 proteins; Ecdysozoa with 79,092 proteins; Spiralia with 11,758 proteins; and Tunicata with 417 proteins. These four proteomes comprised a total of 97,694 proteins, which were used in the final identification of proteins from the feces under high FDR criteria.

### 2.2. Protein and Taxon Profile of Shorebirds Feces

During the high confidence protein identification step using Proteome Discoverer, the search (considering proteins identified with at least two peptides) yielded a total of 2037 proteins grouped into 1120 clusters. Of these, only one protein was of avian origin, leaving 2036 proteins and 1119 invertebrate protein groups for subsequent analyses (Table S1). Taxonomic analysis of the proteins for each bird group and season was performed using Prophane, Python, and Krona plots. Among the 2036 proteins identified in Proteome Discoverer, Prophane only took into account 1968 proteins (comprising 1068 protein groups) which had quantifiable abundance values (Table S2A). Those proteins were therefore processed and reclassified with Python to maintain only those proteins identified in at least 2/3 replicates of one of the shorebird/season groups. The results were then compiled in Excel (Table S2B–L) and were visualized graphically using Krona plots (Supplementary material 5_krona_output_UniProt.html). After all processing steps and filters were applied, the final protein counts for each sample group were as follows (Table S2C–L): 718 in autumn/winter_*A. hypoleucos;* 706 in autumn/winter samples of *C. alba*; 750 in autumn/winter samples of *C. alpina*; 826 in autumn/winter samples of *Charadrius* spp.; 657 in autumn/winter samples of *H. himantopus:* 670 in autumn/winter samples of *T. totanus;* 552 in spring samples of *C. alba*; 610 in spring samples of *Charadrius* spp.; 768 in spring samples of *R. avosetta*; and 587 in spring samples of *S. albifrons*.

### 2.3. Taxonomic Analysis in Autumn/Winter Samples

**Figure 1:**
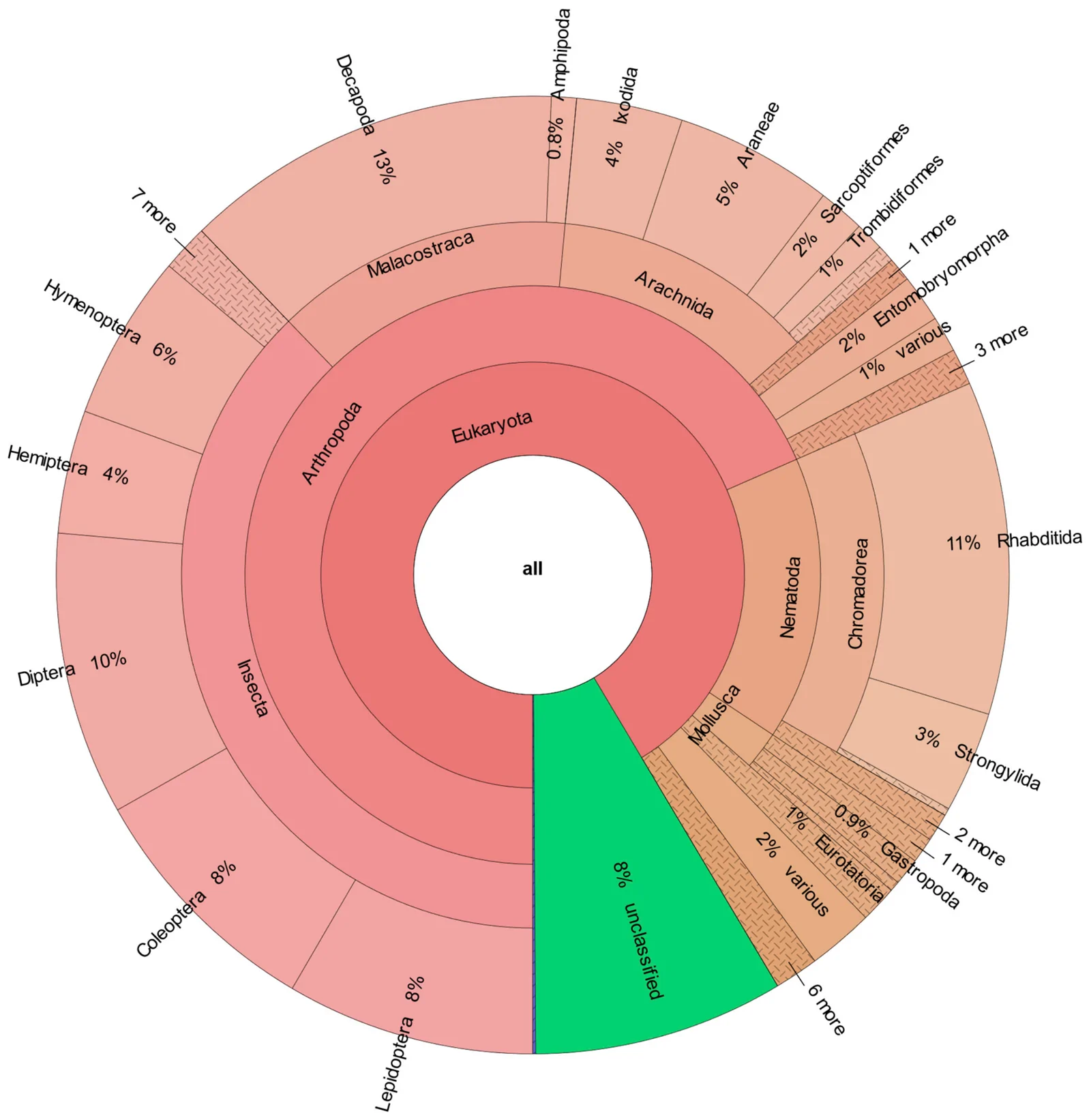
Distribution of invertebrate taxa detected in the feces of autumn/winter samples of *Calidris alba*. The percentages in the image show the average relative abundance of proteins assigned to each taxon across the replicates of this sample. This calculation uses the normalized abundance of proteins identified in at least two-thirds of the replicates analyzed. Unclassified: groups of proteins without taxonomic identification.

**Figure 2:**
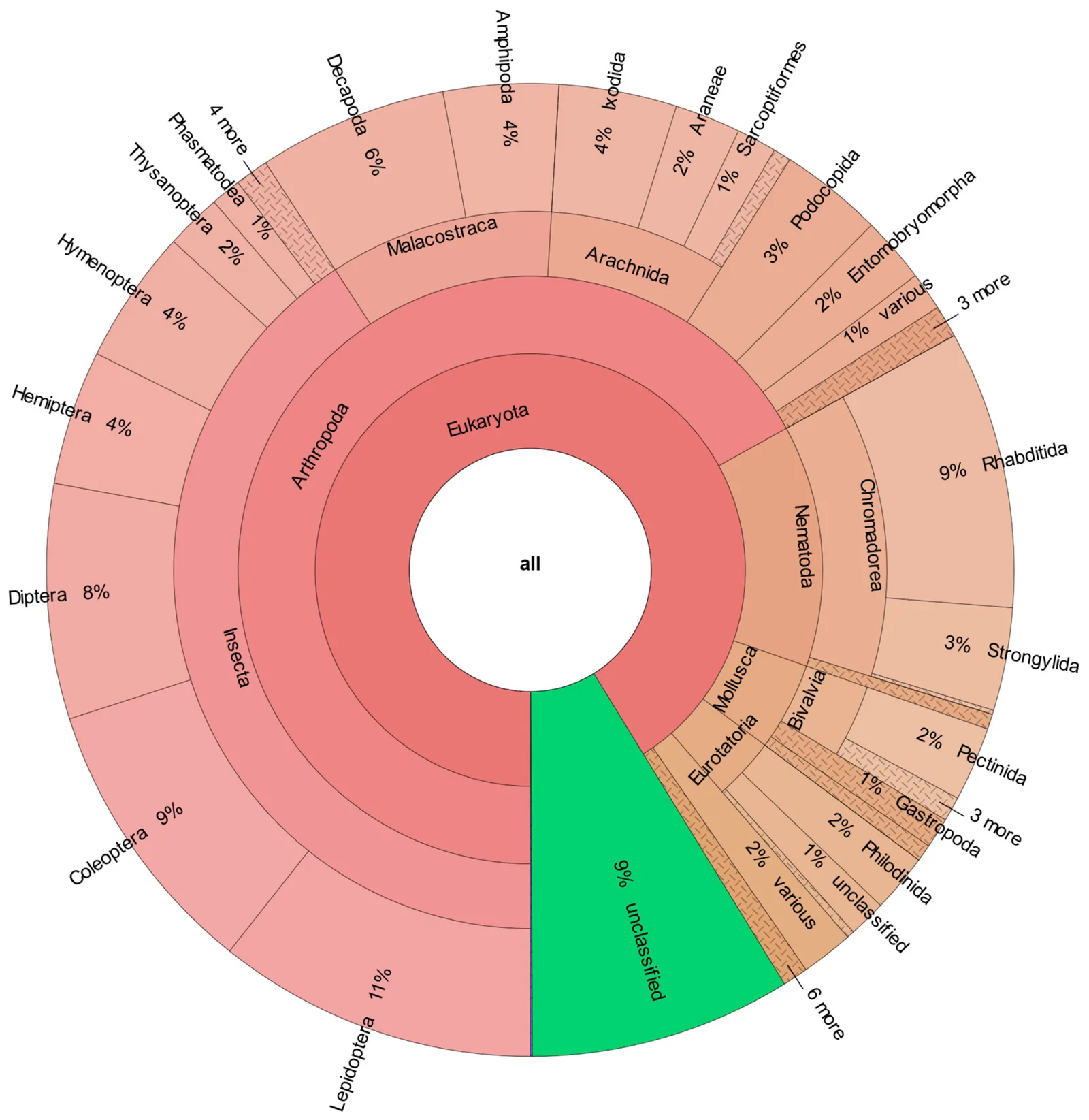
Distribution of invertebrate taxa detected in the feces of autumn/winter samples of *Actitis hypoleucos*. The percentages in the image show the average relative abundance of proteins assigned to each taxon across the replicates of this sample. This calculation uses the normalized abundance of proteins identified in at least two-thirds of the replicates analyzed. Unclassified: groups of proteins without taxonomic identification.

**Figure 3:**
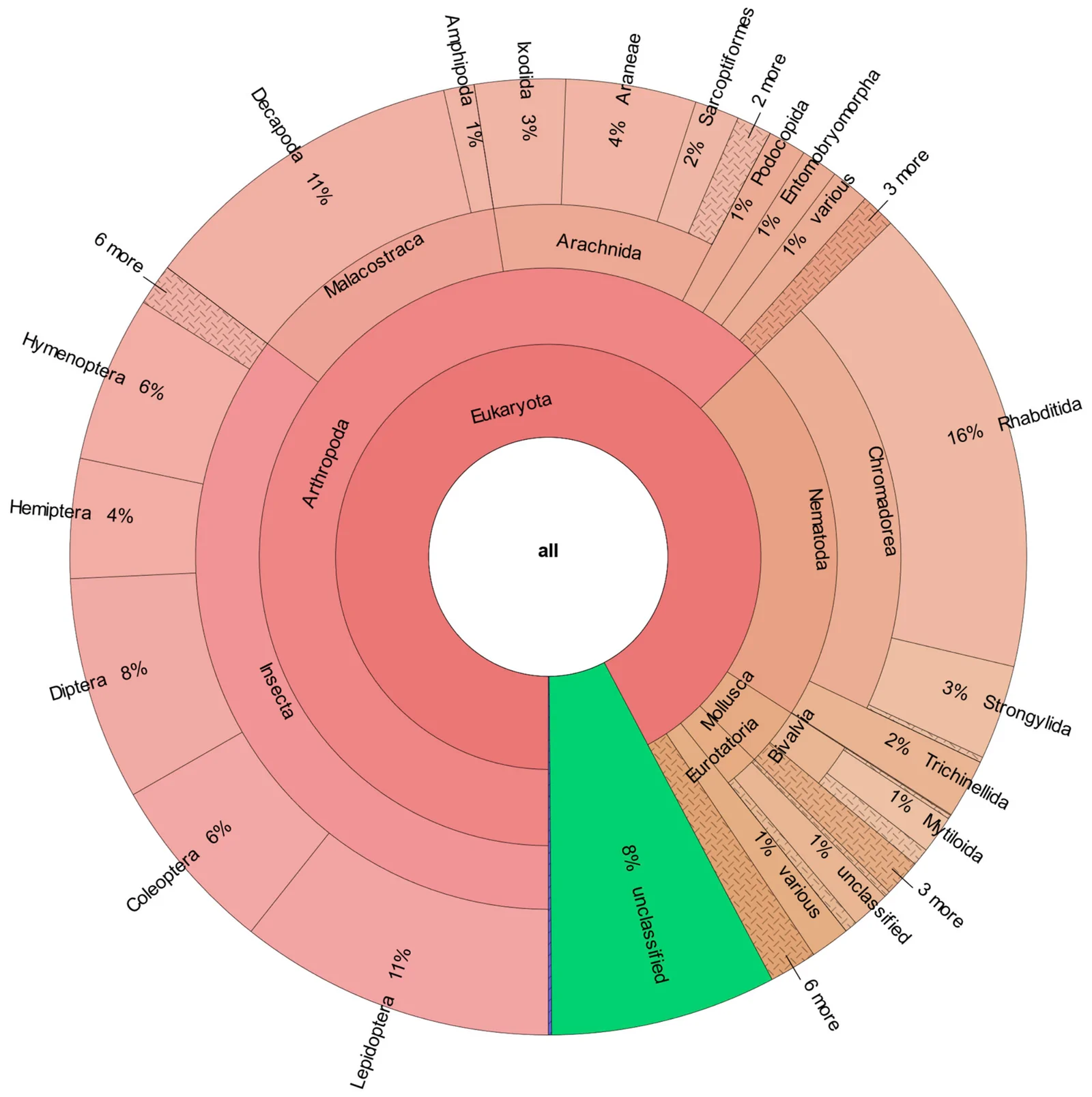
Distribution of invertebrate taxa detected in the feces of autumn/winter samples of *Calidris alpina*. The percentages in the image show the average relative abundance of proteins assigned to each taxon across the replicates of this sample. This calculation uses the normalized abundance of proteins identified in at least two-thirds of the replicates analyzed. Unclassified: groups of proteins without taxonomic identification.

**Figure 4:**
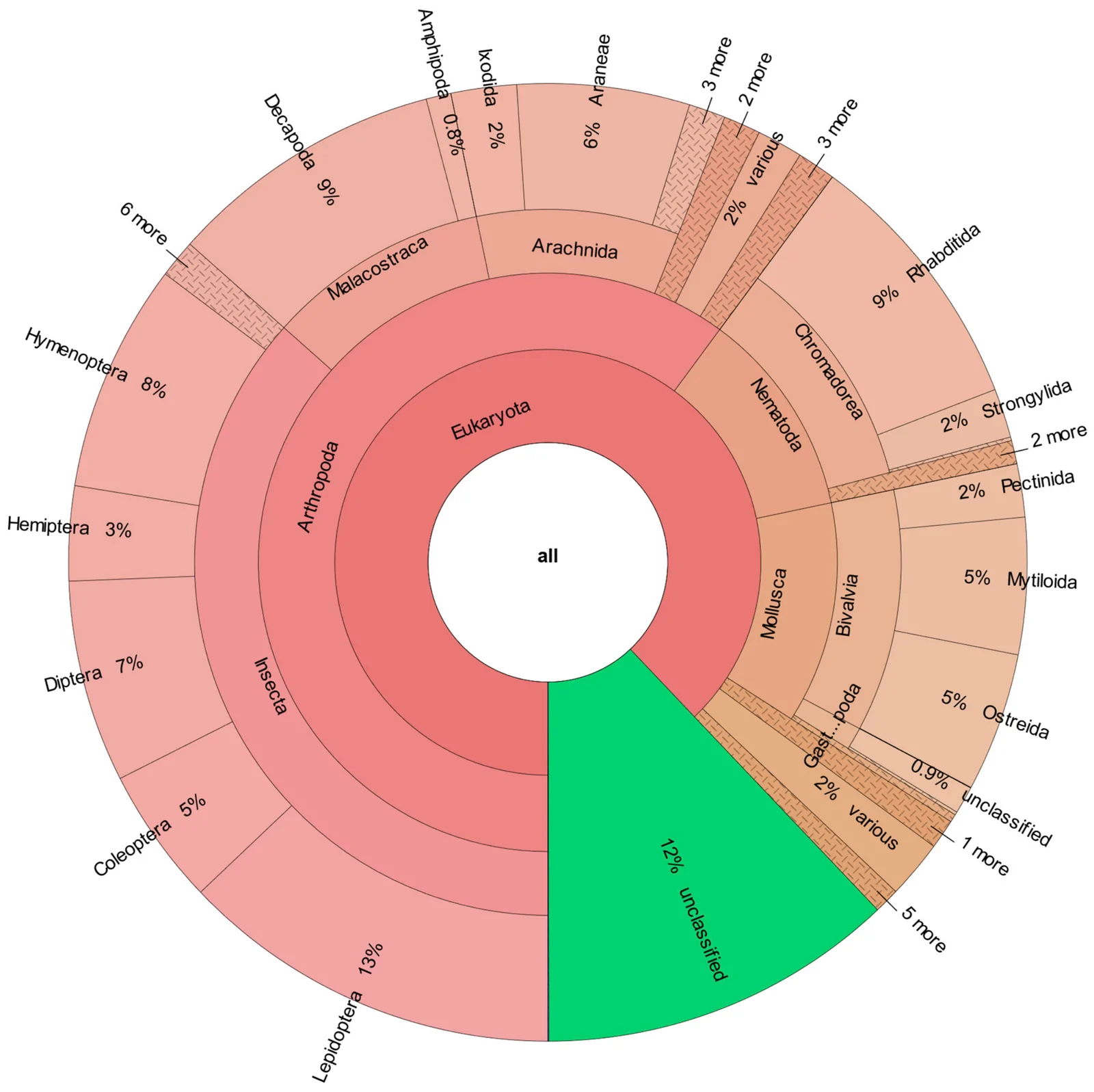
Distribution of invertebrate taxa detected in the feces of autumn/winter samples of *Himantopus himantopus*. The percentages in the image show the average relative abundance of proteins assigned to each taxon across the replicates of this sample. This calculation uses the normalized abundance of proteins identified in at least two-thirds of the replicates analyzed. Unclassified: groups of proteins without taxonomic identification.

**Figure 5:**
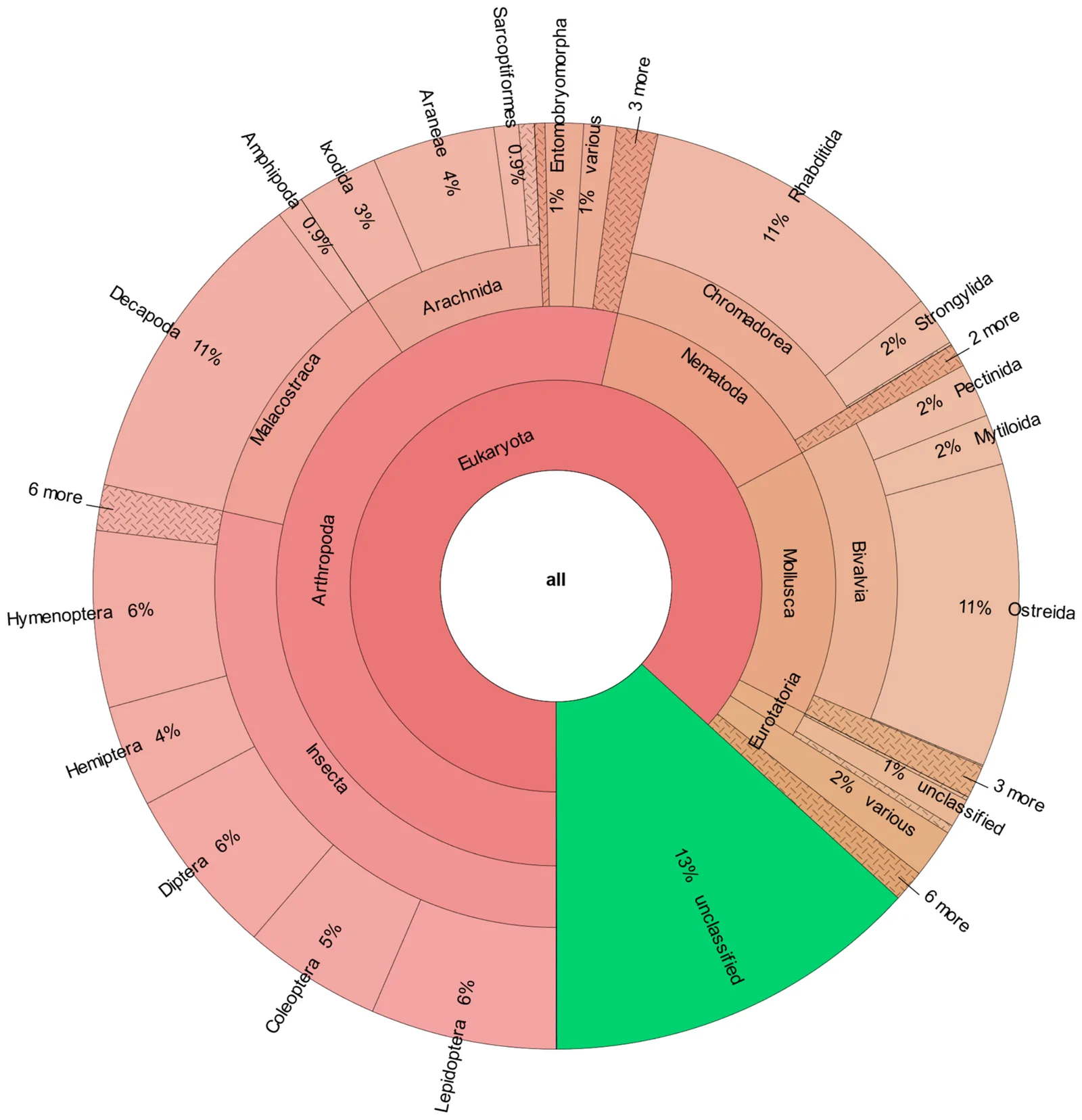
Distribution of invertebrate taxa detected in the feces of autumn/winter samples of *Tringa totanus*. The percentages in the image show the average relative abundance of proteins assigned to each taxon across the replicates of this sample. This calculation uses the normalized abundance of proteins identified in at least two-thirds of the replicates analyzed. Unclassified: groups of proteins without taxonomic identification.

**Figure 6:**
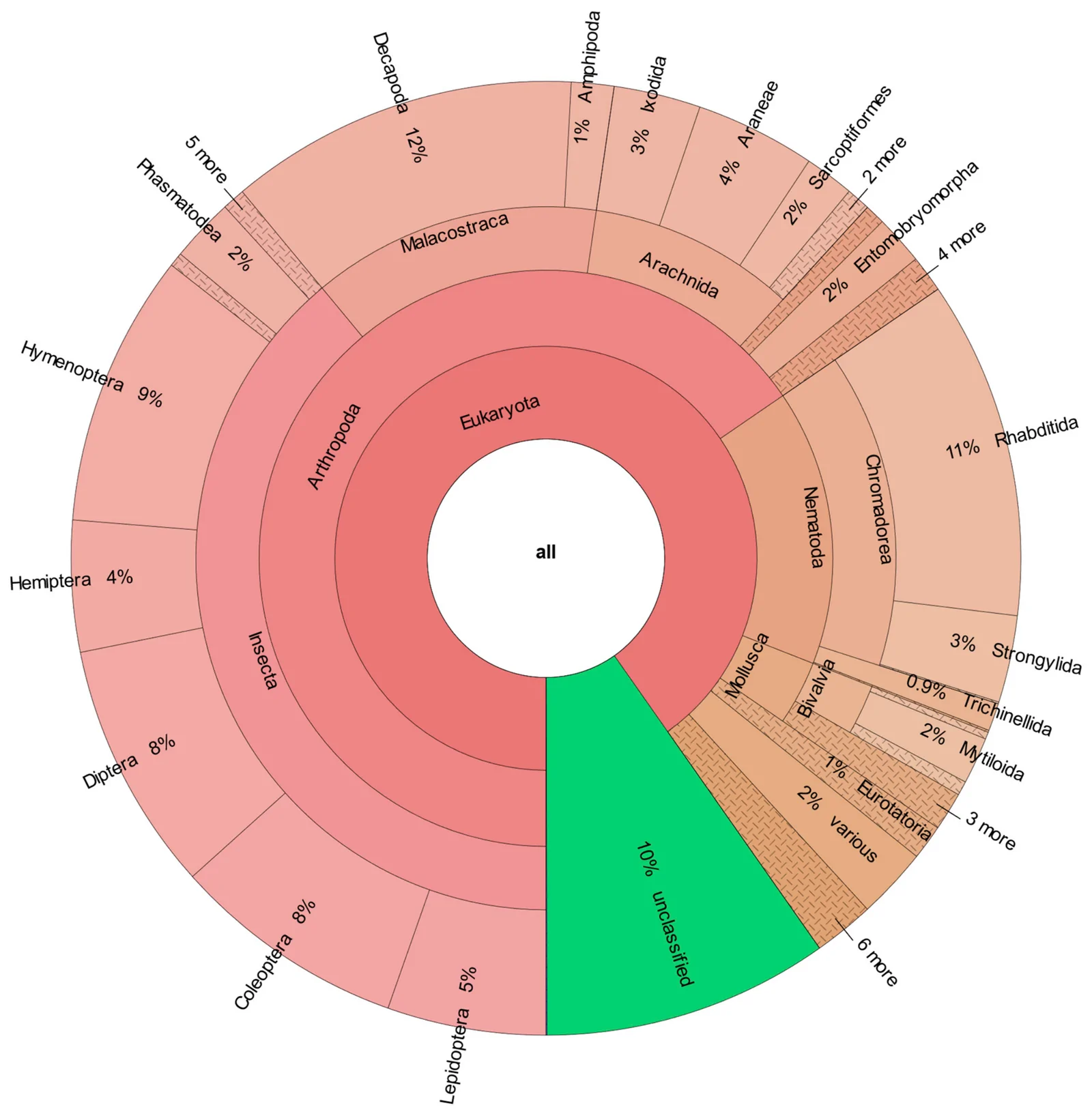
Distribution of invertebrate taxa detected in the feces of autumn/winter samples of *Charadrius* spp. The percentages in the image show the average relative abundance of proteins assigned to each taxon across the replicates of this sample. This calculation uses the normalized abundance of proteins identified in at least two-thirds of the replicates analyzed. Unclassified: groups of proteins without taxonomic identification.

These analyses have shown the identification of approximately 50 orders of invertebrates in the autumn/winter samples (Figure 1, Figure 2, Figure 3, Figure 4, Figure 5 and Figure 6, Table S2).

In the case of *Calidris alba* fecal samples, the most common orders were: two orders of nematodes, Rhabditida (11%) and Strongylida (3%); and eight orders of arthropods, Decapoda (malacostraca, 13%), Diptera (insect, 10%), Coleoptera (insect, 8%), Lepidoptera (insect, 8%), Hymenoptera (insect, 6%), Araneae (arachnid, 5%), Hemiptera (insect, 4%) and Ixodida (arachnid, 4%).

In the case of *Actitis hypoleucos* fecal samples, the major orders were practically the same as in the previous group: two orders of nematodes, Rhabditida (9%) and Strongylida (3%); and 10 orders of arthropods, Lepidoptera (insect, 11%), Coleoptera (insect, 9%), Decapoda (malacostraca, 9%), Diptera (insect, 8%), Hymenoptera (insect, 4%), Hemiptera (insect, 4%), Araneae (arachnid, 2%), Ixodida (arachnid, 4%), Amphipoda (malacostraca, 4%) and Podocopida (Ostracoda, 3%).

In the case of *Calidris alpina* fecal samples, the most common orders were practically the same as in the previous groups: two orders of nematodes, Rhabditida (16%) and Strongylida (3%); and eight orders of arthropods, Lepidoptera (insect, 11%), Diptera (insect, 8%), Coleoptera (insect, 6%), Hymenoptera (insect, 6%), Decapoda (malacostraca, 9%), Hemiptera (insect, 4%), Araneae (arachnid, 4%) and Ixodida (arachnid, 3%).

In the fecal samples of *Himantopus himantopus*, the major orders were practically the same as in the previous groups: one order of nematodes, Rhabditida (9%), and seven orders of arthropods, Lepidoptera (insects, 13%), Decapoda (malacostraca, 9%), Hymenoptera (insect, 8%), Diptera (insect, 7%), Araneae (arachnid, 6%), Coleoptera (insect, 5%), and Hemiptera (insect, 3%). Compared with the other autumn/winter groups, the relative abundance of proteins assigned to Diptera was lower, whereas that of Araneae was higher. Additionally, proteins assigned to the bivalve orders Mytilida and Ostreida accounted for 5% each.

In the case of *Tringa totanus* fecal samples, once again, the most abundant orders were: one order of nematodes, Rhabditida (11%), and six orders of arthropods, Decapoda (malacostraca, 11%), Diptera (insect, 6%), Lepidoptera (insect, 6%), Hymenoptera (insect, 6%), Coleoptera (insect, 5%), and Hemiptera (insect, 5%). Similarly, one order of bivalves, Ostreida (11%), appeared to be the most common.

In the case of *Charadrius* spp. fecal samples, again the most common orders were: two orders of nematodes, Rhabditida (11%) and Strongylida (3%); and eight orders of arthropods, Decapoda (malacostraca, 12%), Hymenoptera (insect, 9%), Diptera (insect, 8%), Coleoptera (insect, 8%), Araneae (arachnid, 4%), Lepidoptera (insect, 5%), Hemiptera (insect, 4%) and Ixodida (arachnid, 3%).

Overall, all species analyzed show a very similar dietary pattern in autumn/winters samples. In all cases, the dominant orders are: Rhabditida (Nematoda), accounting for 9–16% of relative protein abundances, and various orders of arthropods, especially: Diptera (5–10%), Lepidoptera (5–13%), Hymenoptera (4–9%), Coleoptera (5–9%), Araneae (2–6%), Decapoda (6–13%) and Hemiptera (3–6%). The orders Ixodida (2–4%) and Strongylida (Nematoda, 2–3%) appear in all the analyzed groups but are not among the most abundant in any of them.

Finally, the orders that have shown greater relevance in *Himantopus himantopus* (11%) and *Tringa totanus* (14%) are bivalves, with a relative protein abundance of less than 3% (0.9–3%) in the rest of the groups. The second most relevant group of bivalves in autumn was Mytilida, with 5% in *Hymantopus hymantopus* and less than 2% in the rest of the birds (0.1–2%).

### 2.4. Taxonomic Analysis in Spring Samples

**Figure 7:**
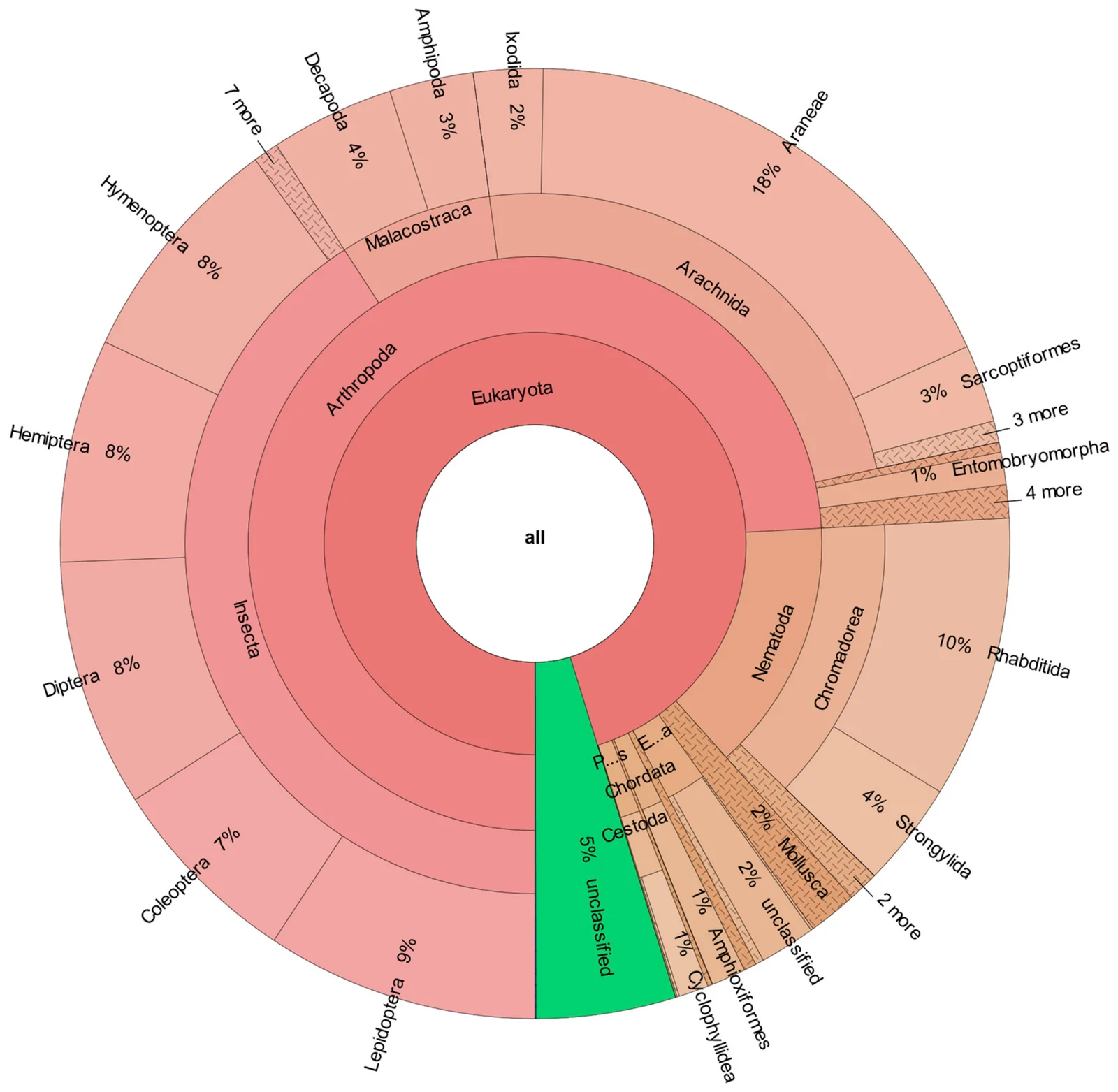
Distribution of invertebrate taxa detected in the feces of spring samples of *Calidris alba*. The percentages in the image show the average relative abundance of proteins assigned to each taxon across the replicates of this sample. This calculation uses the normalized abundance of proteins identified in at least two-thirds of the replicates analyzed. Unclassified: groups of proteins without taxonomic identification.

**Figure 8:**
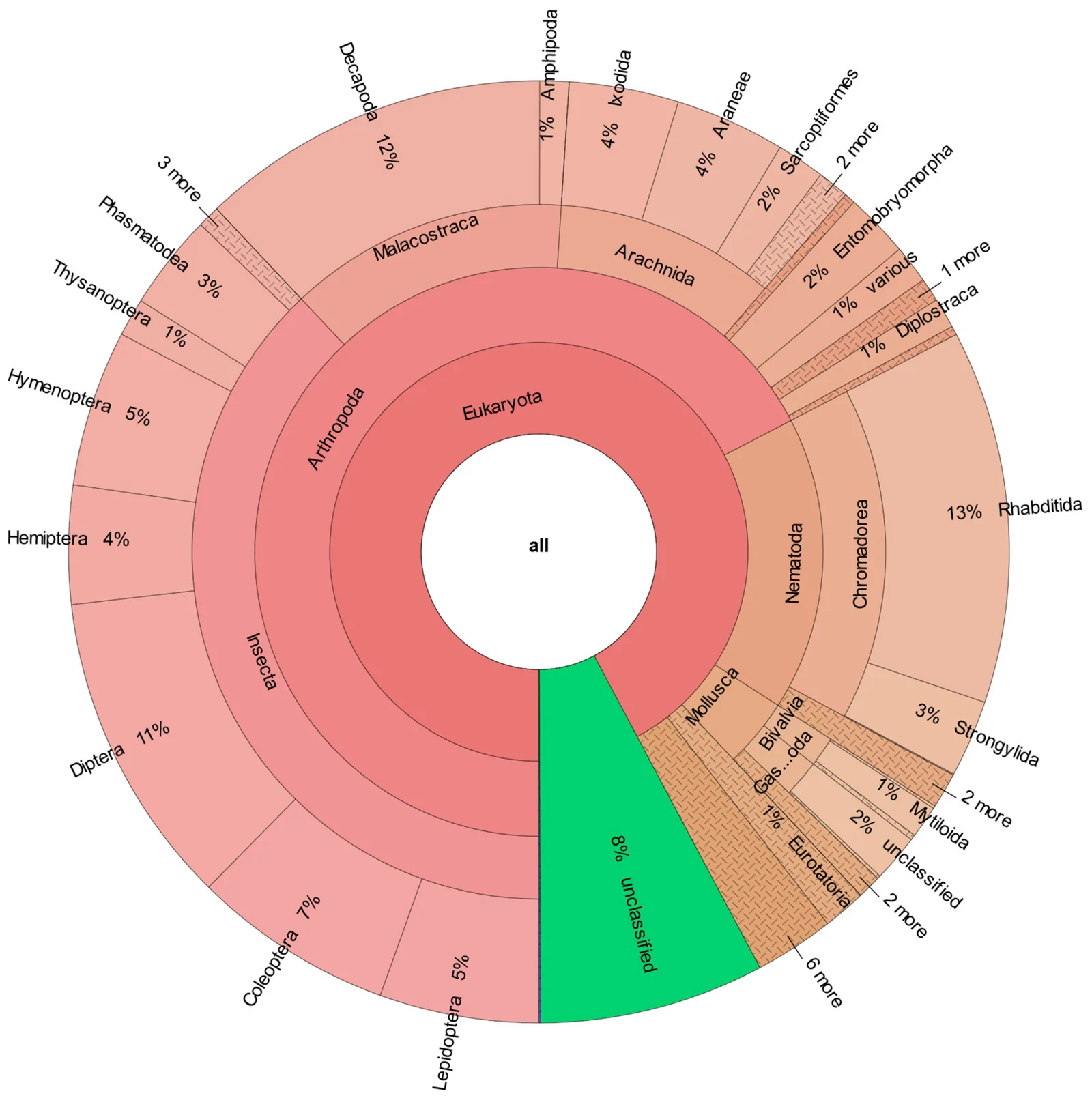
Distribution of invertebrate taxa detected in the feces of spring samples of *Charadrius* spp. The percentages in the image show the average relative abundance of proteins assigned to each taxon across the replicates of this sample. This calculation uses the normalized abundance of proteins identified in at least two-thirds of the replicates analyzed. Unclassified: groups of proteins without taxonomic identification.

**Figure 9:**
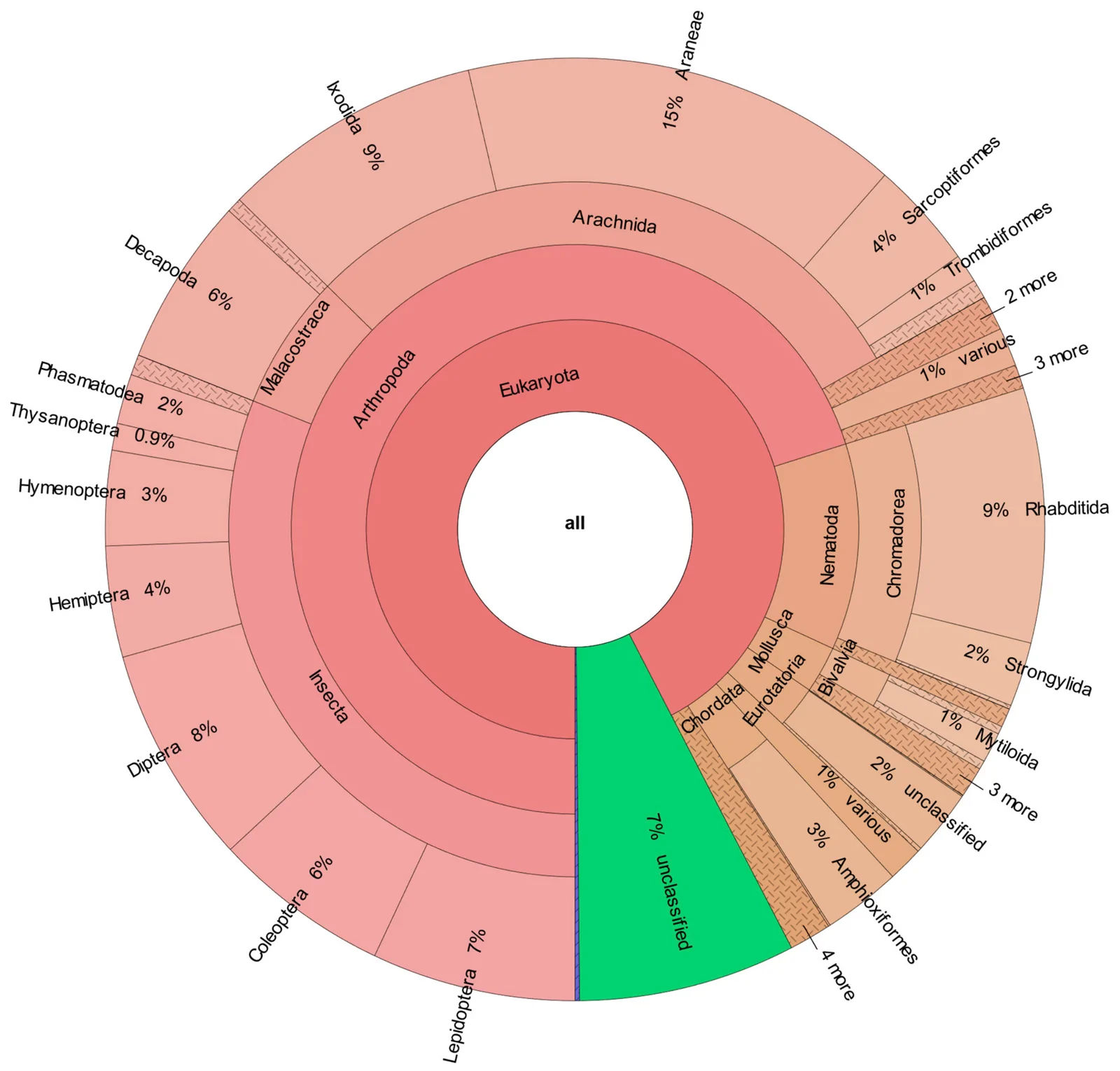
Distribution of invertebrate taxa detected in the feces of spring samples of *Recurvirostra avosetta*. The percentages in the image show the average relative abundance of proteins assigned to each taxon across the replicates of this sample. This calculation uses the normalized abundance of proteins identified in at least two-thirds of the replicates analyzed. Unclassified: groups of proteins without taxonomic identification.

**Figure 10:**
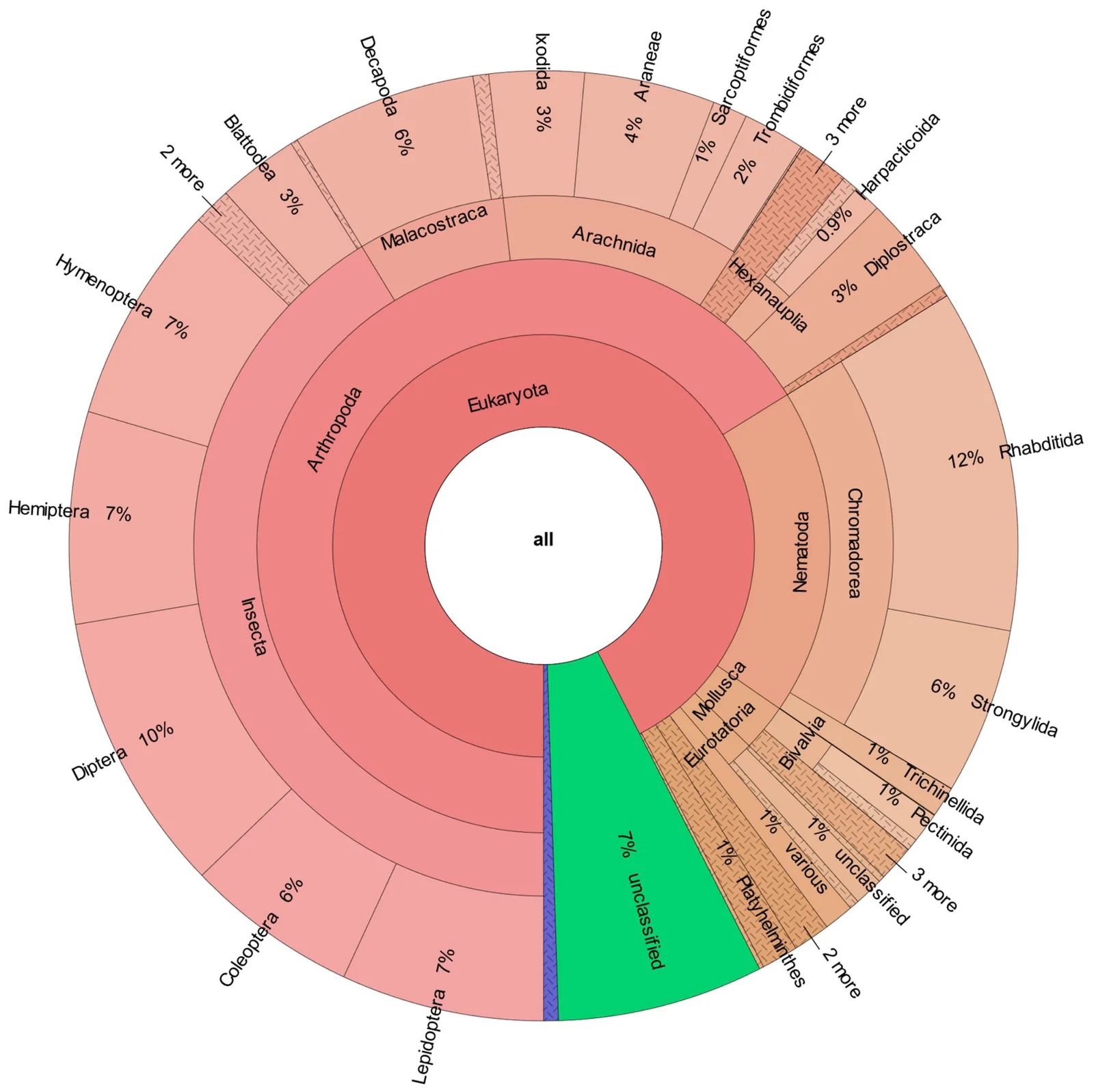
Distribution of invertebrate taxa detected in the feces of spring samples of *Sternula albifrons*. The percentages in the image show the average relative abundance of proteins assigned to each taxon across the replicates of this sample. This calculation uses the normalized abundance of proteins identified in at least two-thirds of the replicates analyzed. Unclassified: groups of proteins without taxonomic identification.

In the case of spring fecal samples (Figure 7, Figure 8, Figure 9 and Figure 10 and Table S2), once again, the most common orders were: two orders of nematodes, Rhabditida (9–13%) and Strongylida (nematode, 2–6%), and eight orders of arthropods, Diptera (insect, 8–11%), Araneae (arachnid, 4–18%), Decapoda (malacostraca, 4–12%), Lepidoptera (insect, 5–9%), Hymenoptera (insect, 3–8%), Coleoptera (insect, 6–7%), Hemiptera (insect, 4–8%) and Sarcoptiformes (arachnid, 1–4%).

### 2.5. Sample Group Comparisons and Statistical Differences

To assess whether statistically significant differences existed in taxonomic identification derived from fecal samples, non-parametric Kruskal–Wallis tests followed by post hoc Dunn tests were conducted. Considering both taxonomic and statistical analyses, several significant differences were detected among shorebird species and between seasons.

First, proteins from the class Bivalvia showed a relative abundance increase in autumn/winter samples from *H. himantopus* (11%) and *T. totanus* (14%) compared with other species sampled during the same season. Adjusted post hoc comparisons indicated significant decreases between *Calidris alba* (0.9%) and *C. alpina* (2%) relative to *H. himantopus* and *T. totanus* (Supporting Material 5: krona_output_UniProt.html; Supporting Table S3). For *A. hypoleucos*, significant decreases in Bivalvia detection compared with *H. himantopus* and *T. totanus* in autumn/winter were restricted to a single bivalve order, Ostreida, with relative protein abundance of 0.2%, 5%, and 11%, respectively. Significant decreases in the relative abundance of proteins assigned to order Ostreida were also observed for *C. alba* (0.5%) (Supporting Material 5: krona_output_UniProt.html; Supporting Table S4D). Although autumn/winter samples of *Charadrius* spp. exhibited a lower abundance of Bivalvia proteins (2%) compared with *H. himantopus* (11%) and *T. totanus* (14%), these differences were not identified as statistically significant in the post hoc analyses. Finally, *H. himantopus and T. totanus* also showed a significantly higher abundance of proteins assigned to Bivalvia compared with most spring samples, including those of *Charadrius* spp., *C. alba*, and *S. albifrons* (Supporting Material 5: krona_output_Uniprot.html; Supporting Table S4D). Differences between spring samples of *R. avosetta* (2%) and autumn/winter samples of *H. himantopus* and *T. totanus* were again only significant for the order Ostreida, with relative protein abundance of 0.09%, 5%, and 11%, respectively (Supporting Material 5: krona_output_Uniprot.html; Supporting Table S4D). Overall, these results indicate that the relative abundances of proteins assigned to Bivalvia, particularly Ostreida, were significantly higher in *H. himantopus* and *T. totanus* during the autumn/winter sampling period than in most other groups analyzed.

Proteins assigned to the class Cestoda also showed an increased relative abundance in *C. alba* samples (0.5–1%), with the highest values observed in spring (1%). The increase in cestode detection in *C. alba* was significantly greater compared with *A. hypoleucos* and *H. himantopus* than with other species, regardless of season (Supporting Material 5: krona_output_Uniprot.html; Supporting Table S4D). At the order level, Cyclophyllidea accounted for most of the observed differences in cestode composition (Supporting Material 5: krona_output_Uniprot.html; Supporting Table S4D). Cyclophyllidea also showed significant decrease in relative protein abundance in *T. totanus* samples compared to spring *C. alba* samples, and between *A. hypoleucos* and spring samples of *Charadrius* spp. and *R. avosetta*. When *p*-value adjustment was not applied, spring samples of *Charadrius* spp. for the class Cestoda, as well as *S. albifrons* for the order Cyclophyllidea, were also identified as significantly different compared with spring *C. alba* samples. Overall, these results indicate differences in the relative abundance of proteins assigned to Cestoda among the groups analyzed, with the highest values observed in spring samples of *C. alba*.

Dunn’s post hoc test with Benjamini–Hochberg-adjusted *p*-values revealed significantly higher relative abundances of proteins assigned to the order Architaenioglossa (class Gastropoda) in most autumn/winter samples (0.09–0.2%) compared with spring samples (0.007–0.04%). The exceptions were autumn/winter samples of *A. hypoleucos* (0.06%) and spring samples of *C. alba* (2%) (Supporting Material 5: *krona_output_Uniprot.html*; Supporting Table S4D). The most pronounced decrease among spring samples was observed in *S. albifrons* (0.007%), relative to all autumn/winter samples except *A. hypoleucos*. Additionally, *Charadrius* spp. also exhibited a significant decrease from autumn/winter (0.2%) to spring (0.04).

In addition, post hoc Dunn tests without adjusted *p*-value, suggested a higher abundance of Branchiopoda proteins detected in *S. albifrons* fecal samples (5%) compared with those of other species (0.3–2%), irrespective of season (Supporting Material 5: krona_output_Uniprot.html; Supporting Table S3E). When applying adjusted *p*-values, only differences with *H. Himantopus* (autumn/winter), *T. totanus* (autumn/winter), *C. alba* (spring) and *R. avosetta* (spring) remained significant (Supporting Material 5: krona_output_Uniprot.html; Supporting Table S3D). Taken together, these results indicate that proteins assigned to Branchiopoda showed higher relative abundances in *S. albifrons*, with significant differences relative to several of the other groups analyzed after adjustment for multiple comparisons.

Finally, the class Arachnida showed lower average values in the autumn/winter samples (8–12%) than in the spring samples (10–29%), with *C. alba* and *R. avosetta* accounting for the majority, with 24% and 29%, respectively (Supporting Material 5: krona_output_Uniprot.html). Dunn’s post hoc test without *p*-value adjustment suggested potential differences in Arachnida-associated protein abundances between groups, although these differences were not statistically significant after adjustment for multiple comparisons (Supporting Table S3E). For *C. alba*, the differences in Arachnida were mainly associated with the orders Sarcoptiformes and Trombidiformes (Supporting Table S4E).

### 2.6. Representation of Invertebrate Taxa in the Metaproteomic Analysis

Table 1 compares invertebrate taxa previously recorded in the marshes and salt flats of Cadiz Bay, including taxa identified in bird feces, with their closest taxonomic representatives available in the UniProt reference database integrated into Prophane (March 2022 release). The taxa listed in Table 1 encompass 19 orders and 10 classes. This comparison was used to assess the representation of locally recorded invertebrate taxa in the database used for taxonomic annotation and to identify potential gaps in taxonomic coverage.

The invertebrate list presented in Table 1 showed that most of the 42 invertebrate entries had taxonomically related representatives in the UniProt reference database integrated into Prophane. Closely related representatives were available for several taxa, including chironomids (family *Chironomidae*), *Ixodes* spp. (genus *Ixodes*), Formicidae (family), Ephemeroptera (order), *Enochrus* spp., Hydrophiliidae. and *Berosus* spp. (family Hydrophilidae) and Amphipoda (order). Other taxa were represented by more distantly related organisms, such as *Artemia* spp. (same class Branchiopoda, genus *Daphnia*) or *Hydrobia* spp. (same order Architaenioglossa, subclass Caenogastropoda). Of the 42 entries, 11 (marked with an asterisk in Table 1) lacked clear taxonomic representatives or presented potential ambiguities in protein-based taxonomic assignment. These included Trichoptera and Plecoptera, whose closest available representatives belonged to different orders, Lepidoptera (same superorder Amphiesmenoptera) and Orthoptera (same cohort Polyneoptera), respectively. Consequently, protein matches to taxonomically related organisms should not be considered unequivocal species-level identifications, as the absence of species-specific reference proteomes may limit the taxonomic resolution and reliability of these assignments.

In the present metaproteomic analysis, several of the taxonomic groups previously documented using conventional methods were also detected (Supporting Material 5: krona_output.html). Additional groups identified included arachnids belonging to the orders Ixodida, Mesostigmata, Araneae, Sarcoptiformes, Scorpiones, and Trombidiformes, as well as copepods (Hexanauplia) and insects belonging to the orders Hemiptera, Hymenoptera, and Ephemeroptera.

Finally, it should be noted that in the present work, proteins assigned to Nematoda were among the most abundant invertebrate-associated proteins detected in the fecal samples analyzed.

## 3. Discussion

Overall, the metaproteomic profiles showed broadly similar taxonomic patterns across the shorebird species and seasons analyzed, with proteins assigned to arthropods and nematodes among the most abundant. This homogeneity suggests that the different species of shorebirds studied may exploit similar trophic resources available in the marshes and salt flats of Cadiz, at least during the period analyzed. Several of the major arthropod orders have previously been described as relevant groups in the diet of wintering shorebirds in Cadiz Bay, such as Diptera and Coleoptera [12]. Those remaining arthropod orders not reported in the work of Pérez Hurtado et al [12] (Hymenoptera, Araneae, Decapoda and Hemiptera), have been described in similar studies conducted in other areas. One example is the work of Sanchez et. al. [18] in the Odiel estuarine marshes, where these orders were detected in the pellets or feces of *T. totanus*. In fact, the order Hymenoptera was as relevant as Diptera in winter samples, reaching similar percentages of occurrence. Similarly, the order Decapoda also showed an increase in its percentage of occurrence in winter samples. These findings are consistent with our metaproteomic results, in which proteins assigned to Hymenoptera, Araneae, Decapoda, and Hemiptera were also detected. Nevertheless, only some taxonomic groups showed significant differences between species or sampling seasons after correction for multiple comparisons.

The results also showed significantly higher relative abundances of proteins assigned to Bivalvia, particularly Ostreida, in *Himantopus himantopus* and *Tringa totanus* during autumn/winter, compared with most other groups analyzed. These differences may be related to the morphological characteristics and foraging strategies of each species, as well as to the differential selection of microhabitats within the marshes and salt flats of Cadiz [19]. Specifically, species such as *Tringa totanus* (Scolopacidae) and *Himantopus himantopus* (Recurvirostrinae) may be better suited to exploiting sandy or muddy benthos, where bivalves are buried or semi-buried, than other shorebirds with a more superficial diet. This protein pattern could be related to morphological and behavioral adaptations described in previous studies, particularly the relationship between beak length, shape, tactile sensitivity, and the ability to detect and exploit buried prey such as bivalves, especially when these are abundant [19]. Studies on the feeding ecology of *T. totanus* show that bivalves are among its frequent prey [12], with mollusks being more important in autumn [18], indicating that their beak and “probing” behavior in muddy sediment allow them to successfully extract buried prey. Similarly, both *Tringa totanus* and *Himantopus himantopus* have shown a higher intake of bivalves compared to *Charadrius* spp. in previous studies [12]. Thus, the higher relative abundances of proteins assigned to Bivalvia in *H. himantopus* and *T. totanus* may reflect differences in the exploitation of benthic food resources among the shorebird species analyzed. However, these findings should not be interpreted as direct evidence of dietary preference, as prey availability and habitat use were not independently assessed.

Another notable observation is the higher relative abundance of proteins assigned to the gastropod order Architaenioglossa in the autumn/winter samples. This pattern is consistent with previous studies in the Odiel Marshes, where *Tringa totanus* was reported to consume more gastropods in winter than in spring [18].

Similarly, proteins assigned to Branchiopoda showed significantly higher relative abundances in spring samples of *S. albifrons* than in several other groups analyzed. Large branchiopods are commonly associated with temporary wetlands, salt marshes, and brackish or hypersaline waters, which are among the habitats used by *S. albifrons* for foraging [20,21]. The presence of branchiopods in the study area, as documented in the local invertebrate survey (Table 1), together with their association with habitats used by *S. albifrons* for foraging, may help explain the higher relative abundance of Branchiopoda-associated proteins detected in our spring samples.

Although differences in Arachnida-associated protein abundances were not statistically significant after adjustment for multiple comparisons, higher relative abundances were observed in spring samples than in autumn/winter samples. Although there are no specific studies in the salt flats of Cadiz or Andalusia that measure the seasonal abundance of spiders, research in eastern Spain and on Mediterranean islands have reported seasonal variation in Araneae communities, including increases in spider abundance during spring [22,23]. These previous observations suggest that seasonal changes in arachnid communities could contribute to the higher relative abundances of proteins assigned to Arachnida detected in spring samples, although this interpretation remains exploratory.

Proteins assigned to Cestoda showed higher relative abundances in *Calidris alba*, particularly in spring samples. Although this pattern could initially be interpreted as suggesting a species-specific or seasonal dietary preference, caution is warranted, as the detection of cestode proteins may reflect parasitic infections rather than direct prey consumption. For instance, a study of six shorebird species (Charadriiformes) recovered multiple cestode species in related species such as *Calidris fuscicollis* and other coastal birds, highlighting the presence of cestodes in these hosts along the Belizean coast (Charadriidae and Scolopacidae) [24].

Finally, comparison of our taxonomic identifications with previous studies conducted in Cadiz Bay using fecal and pellet analyses and direct feeding observations revealed agreement in the detection of several invertebrate groups, including insects (Diptera and Coleoptera), crustaceans (Amphipoda, Decapoda, Ostracoda, Isopoda, and Branchiopoda), mollusks (Gastropoda and Bivalvia), and annelids (Polychaeta). Our metaproteomic analysis also identified proteins assigned to additional taxonomic groups, including arachnids, particularly the order Ixodida. Other orders of arachnids have also been found (Mesostigmata, Araneae, Sarcoptiformes, Scorpiones, Trombidiformes). Among these, Araneae, the most abundant order in our analysis, and Trombidiformes include species adapted to salt-marsh habitats, especially in elevated or vegetated areas [22]. Similarly, our metaproteomic approach has helped to identify copepods (Hexanauplia) and insects (Hemiptera, Hymenoptera, Ephemeroptera), which are also among the typical invertebrates found in the salt flats of Cadiz (unpublished surveys conducted by our research group). Moreover, the detection of nematode proteins in shorebirds’ feces is also a noteworthy finding, since nematodes had not been recorded in previous surveys conducted by our research group using conventional methods. There is evidence of the presence of these invertebrates in marshes and salt flats of Cadiz Bay. Studies of intertidal fauna and regional work have detected nematodes in sediments in the bay and in adjacent coastal dunes, and reviews of marsh macrofauna indicate that annelids and nematodes are dominant components of the benthic community in these ecosystems [25,26]. In addition, previous studies in other areas of Spain have identified nematodes in the pellets and feces of *T. totanus* [18]. Although the presence of nematode and cestode proteins in shorebird feces may be associated with parasitism rather than direct consumption, the failure to detect nematodes may also be explained by the limitations of traditional dietary analyses, in which small, soft-bodied organisms can go undetected due to their almost complete digestion. Fecal analysis alone can lead to erroneous conclusions, given that only worms leave traces in feces, and the researcher may infer from fecal analysis that all observations revealed worms, when in fact some parts were actually clam siphons. These limitations may also explain the lower relevance of polychaetes in these analyses compared to previous studies, where this group may have been overestimated. In this case, fecal analysis alone is not the best method for inferring a bird’s diet selection [18,27].

Therefore, this preliminary study highlights the potential of metaproteomics to identify dietary patterns consistent with those previously documented using conventional approaches, while also detecting taxonomic groups that may be overlooked by traditional analyses. In particular, metaproteomics may help address limitations of dietary analyses based on feces or pellets, especially those related to differential digestibility and the underrepresentation of small, soft-bodied organisms. Furthermore, this approach has the potential to reduce the time required for taxonomic identification compared with traditional methodologies. In comparison with other modern approaches, metaproteomics complements DNA-based dietary methods, which enable the detection of a wide range of taxa but may be affected by primer-related biases, DNA degradation, and the completeness of reference databases [10,16]. By analyzing proteins rather than DNA, metaproteomics provides an alternative source of taxonomic information and may also offer insights into the functional characteristics of the detected proteins and their tissue of origin. On the other hand, stable isotope analysis reflects assimilated resources but requires tissue sampling and provides limited taxonomic resolution [9,11]. Although temporary capture is required, metaproteomics avoids tissue extraction and represents a relatively less invasive approach with clear potential for ecological and conservation-oriented studies of shorebirds. Nevertheless, metaproteomic identification also has limitations, particularly its dependence on the completeness and taxonomic coverage of protein reference databases, which may affect the detection of certain organisms and the taxonomic resolution achieved.

## 4. Materials and Methods

### 4.1. Sample Collection

Fecal samples were obtained from shorebirds captured using passive trapping methods adapted to different periods of the annual cycle. During the wintering period, individuals were captured using walk-in clap traps triggered by a cord-release mechanism while shorebirds were actively foraging. During the breeding period, captures were conducted primarily using funnel traps, passive devices that allow birds to enter while incubating, thereby minimizing disturbance.

Following capture, each individual was temporarily placed in a clean, individual holding box until defecation occurred. Fresh fecal material was collected using sterile techniques, transferred into sterile Eppendorf tubes, and immediately stored under refrigerated field conditions at 4 °C. Samples were maintained under cold-chain conditions during transport and, upon arrival at the laboratory, were stored at −80 °C until further analysis.

Captured individuals were released immediately after sample collection to minimize handling time and stress.

### 4.2. Protein Extraction and Digestion

Each fecal sample frozen at –80 °C was mixed with 1.0 mL of extraction solution (4% SDS, 0.1 M Tris-HCl [pH 7.6], 40 mM DTT, and Pierce Mini Tablets protease inhibitors, EDTA-free [Thermo Scientific, Rockford, IL, USA; Cat. No. A32955]) in 2 mL tubes containing 2.4 mm metal beads (Fisherbrand™, Fisher Scientific, Pittsburgh, PA, USA; Cat. No. 15535799). Samples were homogenized using a Bead Mill 4 homogenizer (Fisherbrand). Five cycles of 20 s at speed 5 were performed, keeping the samples on ice between cycles. The homogenate was transferred to 2 mL Eppendorf tubes and centrifuged at 9000 *g* for 10 min. The supernatant was pipetted into new 15 mL tubes, and 4 volumes of 0.1 M ammonium acetate in cold methanol were added. After overnight incubation at –20 °C, the samples were centrifuged at 15,000 *g* for 10 min, and the supernatant was discarded. Immediately, 1.5 volumes of 10% TCA in 100% acetone were added, and samples were stored at –20 °C for 2 h. The protein pellet was recovered by centrifugation at 15,000 *g* for 10 min. Finally, the pellet was washed through three successive precipitation steps with ammonium acetate (0.1 M) in 80% acetone, 70% ethanol, and 80% acetone, vortexed, incubated on ice for 10 min, and centrifuged between each solvent. Protein pellets were air-dried briefly (1–30 min) and dissolved in denaturing buffer (8 M urea, 100 mM Tris-HCl pH 8.5, 5 mM DTT). Protein concentrations were determined using the Qubit Protein Assay kit (Thermo Scientific™, Waltham, MA, USA) following the manufacturer’s instructions.

Protein digestion was performed using the small-scale FASP protocol described by Escobar Nino et. al. [28]. Briefly, the protein pellet was reduced for 30 min at room temperature in denaturing buffer. An aliquot containing 50 μg of protein, diluted in UA buffer (8 M urea, 100 mM Tris-HCl pH 8.5), was added to a 30 kDa cutoff filter (Vivacon 500, VN01H22; Sartorius, Göttingen, Germany) and centrifuged at 14,000 *g* for 10 min. The filter was washed with 450 μL UA buffer at 14,000 *g* for 10 min. Proteins were alkylated for 30 min in the dark with 100 μL CAA solution (55 mM chloroacetamide, 8 M urea, 100 mM Tris-HCl pH 8.5). After centrifugation at 14,000 *g* for 10 min, the filter was washed twice with 450 μL UA buffer. Protein digestion was carried out with 0.5 μg trypsin (Pierce Trypsin MS-Grade, Thermo Scientific, Rockford, IL, USA; Cat. No. 90057) in 450 μL dilution solution (100 mM Tris-HCl pH 8.5, 1 mM CaCl_2_) at 37 °C overnight. Peptides were collected by centrifuging the filter at 14,000 *g* for 10 min and acidified with 20% TFA (final concentration 0.5%).

### 4.3. LC-MS/MS Analysis

Digested peptides were desalted using C18 solid-phase extraction columns (Pierce 89873, Thermo Scientific™, Waltham, MA, USA) according to the manufacturer’s instructions. Eluted peptides were dried in a vacuum concentrator (Vacufuge Vacuum Concentrator 5301 Centrifuge; Eppendorf, Hamburg, Germany) for 40 min. The peptide pellet was resuspended in 25 μL solubilization buffer (0.1% formic acid/0.02% DDM [n-dodecyl β-D-maltoside]), and peptide concentration was measured using a Nanodrop 2000 (Thermo Scientific™, Waltham, MA, USA). Finally, the peptide solution was diluted to 0.1 μg/μL, and 4 μL were used for LC-MS analysis.

The samples were analyzed using a Vanquish Neo liquid chromatography system (Thermo Scientific, Waltham, MA, USA) coupled to an Orbitrap Exploris 240 mass spectrometer (Thermo Scientific, Waltham, MA, USA). Peptides were separated on a PepMap RSLC C18 column (2 µm, 100 Å, 75 µm inner diameter, 50 cm) (Thermo Scientific, Waltham, MA, USA). Peptides were loaded onto the column and eluted over 28 min using a segmented linear gradient from 2% to 95% of solvent B (0 min: 1% B; 0–10 min: 30% B; 10–20 min: 45% B; 20–21 min: 97.5% B; 21–28 min: 97.5% B) (solvent A: 0.1% formic acid; solvent B: 80% acetonitrile, 0.1% formic acid) at a flow rate of 300 nL/min.

Mass spectra were acquired in data-dependent acquisition (DDA) mode using a TOP15 method. MS spectra were obtained in the Orbitrap Exploris 240 analyzer (Thermo Scientific, Waltham, MA, USA) in full-scan mode, with a mass range of 350–1500 *m*/*z*, a resolution of 120,000 FWHM, an AGC target of 300%, and a maximum injection time of 60 ms. Precursors were selected with an isolation window of 1.6 m/z. HCD fragmentation was performed with a normalized collision energy of 30%. MS/MS spectra were acquired with a standard AGC target at a resolution of 15,000 FWHM, a maximum injection time of 30 ms, and a fixed first mass of m/z 100. Peptides with a charge state of +1, greater than +6, or with unassigned charge were excluded from MS2 fragmentation. A dynamic exclusion of 30 s prevented repeated selection of precursors.

### 4.4. Proteomic Database Generation

A custom invertebrate and avian protein sequence database was generated using a two-step sequential database reduction strategy. To this aim, in an initial search, mass spectral data (raw data files) were processed using Proteome Discoverer software (version 3.1.1.93). MS/MS raw data files were analyzed in groups of ten using the Sequest search engine against databases containing invertebrate proteins deposited in UniProtKB (September 2025) for Spiralia [2,495,697], Ecdysozoa [1,206,794], Tunicata [7712], and Cephalochordata [7735] (downloaded on 25 September 2025). The digestion mode was set to trypsin. The minimum peptide length was set to six amino acids. Methionine oxidation, N-terminal protein acetylation and methionine loss were set as variable modifications. Carbamidomethylation of cysteine residues was set as a fixed modification. Peptide scores were recalibrated using the Percolator node in Proteome Discoverer, which employs a target/decoy approach and machine learning to estimate the probability of correct identification and control the false discovery rate (FDR). In our consensus workflow, peptides identified with low confidence (FDR < 5%) were included, proteins were required to be identified with at least one peptide, and all peptides were considered regardless of the number of peptide-spectrum matches (PSMs). Shared peptides among proteins were fully included rather than being assigned only to the highest-scoring protein. The FASTA files generated for each taxon in each search group were unified into a single database using the “Compile Fasta Database” tool in Proteome Discoverer. The taxon-specific FASTA file generated in this step was then used for a second search in Proteome Discoverer (version 3.1.1.93), using the same criteria but increasing peptide identification confidence to medium (FDR > 5%).

The same approach was used to reduce the avian database. Proteins belonging to the closest avian relatives of the analyzed shorebird species were used as protein databases (proteins deposited in UniProtKB September 2025 of *Arenaria interpres*, *Calidris pygmaea*, *Charadrius vociferus*, *Himantopus himantopus*, and *Phaetusa simplex*).

The FASTA files generated for invertebrates and shorebird proteins during the second step of database reduction were used in the final search alongside the contaminant database.

### 4.5. Protein Identification from Avian Fecal Samples

Final protein identification and taxonomic annotation were performed using Proteome Discoverer and Prophane. First, protein identification was carried out in Proteome Discoverer (version 3.1.1.93). The Processing Workflow “PWF_Hybrid_Precursor_Quant_and_LFQ_MPS_SEquestHP_Percolator” and the Consensus Workflow “CWF_Comprehensive_Enhanced_Annotation_LFQ_and_Precursor_Quan” were applied with modifications. Spectrum Files RC and Minora Feature Detector nodes were set to offline mass recalibration and label-free MS1 quantitation, respectively. Peptide identification was carried out using Sequest-HT as the search engine and Percolator for peptide validation, setting the FDR to 1%. The search parameters applied were: trypsin (full) digestion mode; minimum peptide length of six amino acids; methionine oxidation; N-terminal protein acetylation and methionine loss as variable modifications; and Carbamidomethylation of cysteine residues as a fixed modification. Only proteins identified with two or more peptides were kept. Protein grouping for inference was performed without applying strict parsimony, thereby applying an anti-Occam’s razor approach as the best option for metaproteomic grouping [5]. Feature Mapper was used to align and match precursor features across LC–MS/MS runs, ensuring consistent identification of the same precursor ions between samples and improving quantitative coverage. Precursor Ions Quantifier was applied to perform MS1-based quantification by calculating precursor ion abundances from signal intensities, providing the basis for label-free quantification and protein-level abundance estimation. Label free protein quantification was performed using the abundance of each protein, calculated by summing the abundances of their identified peptides. Abundances were normalized to the total peptide abundance per sample. The databases used were those generated in the previous step, including a list of contaminant proteins from The Global Proteome Machine (TheGPM). The protein group identification file was filtered to remove contaminant proteins and proteins from shorebirds. The remaining invertebrate proteins were retained for analysis with Prophane (https://www.prophane.de (accessed on 10 December 2025)) [29].

Prophane was used in expert mode to allow modification of default parameters, loading the protein groups file from the Proteome discoverer search. Samples were grouped according to their respective analysis groups (Autumn_Actitis_hypoleucos, Autumn_Calidris_alba, Autumn_Calidris_alpina, Autumn_Charadrius_sp, Autumn_Himantopus_himantopus, Autumn_Tringa_nebularia, Autumn_Tringa_totanus, Spring_Calidris_alba, Spring_Charadrius_sp, Spring_Recurvirostra_avosetta, Spring_Sternula_albifrons). The protein FASTA required as input for Prophane was generated in Proteome Discoverer from the final protein identification step.

Prophane results were filtered using a Python-based algorithm (version 3.14.3) employing pandas (version 2.3.3), argparse, os, tqdm (version 4.67.1), logging, and sys libraries. This algorithm processes the output files generated by Prophane, performing individual recalculation for each group, retaining only normalized abundance values (quant::sample name (mean)) from protein groups with quantification data in at least 2/3 of the analyzed replicates. Values for those not meeting this criterion were set to 0. From the recalculated data, input files for Krona plot construction were generated. Visualization of the results was carried out using KronaTools (version 2.8.1) [30], a set of scripts designed to generate interactive hierarchical data plots. These files were processed to produce interactive Krona charts, allowing visualization of the taxonomic distribution for each analyzed group.

### 4.6. Statistical Analysis

Quantitative data were grouped by class (Supplementary Table S3) or order (Supplementary Table S4) to enable statistical analyses and to identify significant differences between groups at these taxonomic levels. The Shapiro–Wilk normality test was applied to normalized abundances, and the results indicated that the data did not follow a normal distribution. Therefore, the non-parametric Kruskal–Wallis test was used to evaluate whether there were significant differences in abundance among the different taxonomic orders analyzed. After detecting overall differences between groups in certain orders, post hoc comparisons were performed using Dunn’s test. Additionally, Dunn’s post hoc test was also performed without *p*-value adjustment to explore potential differences between shorebird groups that might go undetected due to heterogeneity among replicates. To control for type I error in multiple comparisons, the Benjamini–Hochberg correction was applied. Adjusted *p*-values below 0.05 were considered statistically significant, whereas unadjusted *p*-values below 0.05 were considered for exploratory comparative analyses.

## 5. Conclusions

Fecal metaproteomic analysis of shorebirds inhabiting the marshes and salt flats of Cadiz Bay revealed diverse taxonomic profiles, predominantly characterized by proteins assigned to arthropods, with broadly similar patterns across species and sampling seasons. Nevertheless, metaproteomic analysis revealed significant differences in specific taxonomic groups after correction for multiple comparisons, including higher relative abundances of proteins assigned to Bivalvia, particularly Ostreida, in *Himantopus himantopus* and *Tringa totanus* during autumn/winter, as well as differences involving *Branchiopoda* and *Architaenioglossa*. These findings are consistent with previous dietary studies, supporting the potential of metaproteomics as an alternative to traditional dietary analyses. Furthermore, this approach allows the detection of taxon-specific differences within otherwise similar trophic profiles.

Beyond these ecological findings, metaproteomics provided taxonomic information that may be difficult to obtain through traditional morphological examination of fecal remains. In particular, the detection of proteins assigned to a wide range of invertebrate groups, including organisms that may leave few recognizable remains, provides a more detailed characterization of fecal composition than dietary studies based solely on fecal analysis. Furthermore, the identification of proteins associated with potential parasites, such as cestodes and nematodes, highlights the importance of distinguishing dietary components from other biological sources present in fecal samples. Thus, metaproteomics represents a promising alternative to conventional morphological dietary analyses, with the potential to reduce processing time and reliance on operator-dependent visual identification. However, further improvements in reference proteome databases and adequate replication across groups will be essential to strengthen taxonomic resolution and statistical robustness, ultimately facilitating the replacement of traditional approaches.

## Figures and Tables

**Table 1:** List of invertebrate taxa recorded in Cadiz Bay and their closest relative in UniProt. List of invertebrates detected in previous studies involving manual analysis of feces and direct observation of bird feeding, and those identified in the salt flats of Cadiz Bay, with their closest relatives in UniProt.

UniProt (March 2022)	Genus	Family	Order	Class	Organism
Family *Chironomidae* (*Clunio* and polypedinum)	Undetermined	Chironomidae	Diptera	Insecta	*Chironomid larvae*
	Undetermined	Chironomidae	Diptera	Insecta	*Chironomid nymphae*
	*Chironomus*	Chironomidae	Diptera	Insecta	*Chironomus salinarius*
	*Chironomus*	Chironomidae	Diptera	Insecta	*Chironomus tanytarsus*
	*Chironomus*	Chironomidae	Diptera	Insecta	*Chironomus* sp.
Mosquito genera (*Anopheles*, *Culex* and *Aedes*)	Undetermined	Undetermined	Diptera	Insecta	other mosquitoes
Superfamily ephydroidea (*Drosophila* genera), and other Schizophora families (Tephritidae, Glossinidae, Muscidae and Calliphoridae)	*Ephydra*	Ephydridae	Diptera	Insecta	*Ephydra larvae*
	*Ephydra*	Ephydridae	Diptera	Insecta	*Ephydra pupae*
Phoridae family (same infraorder same higher taxonomic sections above the family level, Aschiza)	*Syrphidae*	Syrphidae	Diptera	Insecta	*Syrphidae* sp.
*Phoridae family (No Aschiza, so more closet related to Syrphidae)	Undetermined	Empididae	Diptera	Insecta	*Empididae (family)
Formicidae family	Undetermined	Formicidae	Hymenoptera	Insecta	Formicidae (family)
Bee families (no Apidae), Halictidae	Undetermined	Undetermined	Hymenoptera	Insecta	Undetermined bee
Bee family Apidae	*Xylocopa*	Apidae	Hymenoptera	Insecta	*Xylocopa* sp.
Order Hemiptera, suborder Heteroptera, same clade Panheteroptera (Miridae, Reduviidae, Cimicidae)	*Corixa*	Corixidae	Hemiptera (heteroptera)	Insecta	*Corixa* sp.
	*Hesperocorixa*	Corixidae	Hemiptera (heteroptera)	Insecta	*Hesperocorixa* sp.
		Notonectidae	Hemiptera (heteroptera)	Insecta	Notonectidae.
*Same clade Neoheteroptera, not Panheteroptera (Miridae, Reduviidae, Cimicidae). Closer related to Corixa, hesperocorixa and Notonectidae.	*Halobates*	Gerridae	Hemiptera (heteroptera)	Insecta	*Halobates sp.
	Undetermined	Hebridae	Hemiptera (heteroptera)	Insecta	*Hebridae
*No closer relative (no Adephaga members in UniProt)	*Gyrinus*	Gyrinidae	Coleoptera	Insecta	*Gyrinus sp.
	*Gyrinus*	Gyrinidae	Coleoptera	Insecta	*Gyrinus dejeani
	*Potamonectes (Nebrioporus)*	Dytiscidae	Coleoptera	Insecta	*Potamonecte sp.
Family Lampyridae, Elateridae and Buprestidae (same suborder Polyphaga and infraorder Elateriformia)	*Dubiraphia*	Elmidae	Coleoptera	Insecta	*Dubiraphia* sp.
Family Hydrophilidae	*Enochrus*	Hydrophilidae	Coleoptera	Insecta	*Enochrus* sp.
	Undetermined	Hydrophiliidae	Coleoptera	Insecta	*Hydrophiliidae* sp.
	*Berosus*	Hydrophilidae	Coleoptera	Insecta	*Berosus* sp.
*Polyphaga; Staphyliniformia; Staphylinoidea, the closest relative is Hydrophilidae family, but this is more related to members of hydrophilidae family	*Octhebius*	Hydraenidae	Coleoptera	Insecta	*Octhebius sp.
*Lepidoptera (closest order, same superorder Amphiesmenoptera)	Undetermined	Undetermined	Trichoptera	Insecta	*Trichoptera sp.
*Orthoptera, Phasmatodea, Blattodea (closest order, same cohort Polyneoptera)	Undetermined	Undetermined	Plecoptera	Insecta	*Plecoptera sp.
Ephemeroptera order (genus Ephemera, family Ephemeridae)	Undetermined	Undetermined	Ephemeroptera	Insecta	*Ephemeroptera* (order)
Family Hyalellidae (same Amphipoda order and Senticaudata suborder)	*Melita*	Melitidae	Amphipoda	Malacostraca	*Melita palmata*
	*Microdeutopus*	Aoridae	Amphipoda	Malacostraca	*Microdeutopus gryllotalpa*
Family Lysianassidae (same Amphipoda order, different from the family Hyalellidae)	Undetermined	Undetermined	Amphipoda	Malacostraca	Amphipoda (order)
Order Isopoda: genus Armadillidium	*Lekanesphaera*	Sphaeromatidae	Isopoda	Malacostraca	*Lekanesphaera hookeri*
Families Alpheidae, Atyidae and Alvinocarididae (same Infraorder Caridea)	*Palaemonetes (o Palaemon)*	Palaemonidae	Decapoda	Malacostraca	*Palaemonetes varians*
Family Portunidae	*Carcinus*	Portunidae	Decapoda	Malacostraca	*Carcinus maenas*
*Superorder Peracarida: Order Mysida, Isopoda and Amphipoda. Mysida is more distantly related to Amphipoda and Isopoda than those two are to each other.	*Mesopodopsis*	Mysidae	Mysida	Malacostraca	*Mesopodopsis slabberi
Orders Siphonostomatoida and Harpacticoida (same Class Hexanauplia and Subclass Copepoda)	Undetermined	Undetermined	Undetermined	Hexanauplia (subclass Copepoda)	Copepoda
Order Podocopida: family Cyprididae and Darwinulidae (same Class Ostracoda)	Undetermined	Undetermined	Undetermined	Ostracoda	Ostracoda
Family Daphniidae (same Class Branchiopoda and Order Diplostraca)	*Artemia*	Artemiidae	Anostraca	Branchiopoda	*Artemia* sp./*Franciscana*
Order Architaenioglossa (same Class Gastropoda and Subclass Caenogastropoda)	*Hydrobia*	Hydrobiidae	Littorinimorpha	Gastropoda	*Hydrobia ulvae*
	*Hydrobia*	Hydrobiidae	Littorinimorpha	Gastropoda	*Hydrobia minoricensis*
Families Mytilidae, Pectinidae, Pteriidae (same order Ostreida)	*Cerastoderma*	Cardiidae	Cardiida	Bivalvia	*Cerastoderma edule*
	*Abra*	Semelidae	Cardiida	Bivalvia	*Abra ovata*
	*Scrobicularia*	Scrobiculariidae	Cardiida	Bivalvia	*Scrobicularia plana*
Other Polychaeta, excluding Sedentaria	*Hediste*	Nereididae	Phyllodocida	Polychaeta	*Hediste diversicolor*
	*Nereis*	Nereididae	Phyllodocida	Polychaeta	*Nereis diversicolor*
	*Diopatra*	Onuphidae	Phyllodocida	Polychaeta	*Diopatra* spp.
	*Glycera*	Glyceridae	Phyllodocida	Polychaeta	*Glycera* sp.
	Undetermined	Undetermined	Phyllodocida	Polychaeta	*Phyllodocida* sp.
*Family Capitellidae, genera *Capitella* (more specific for genera *Capitella*)	*Capitella*	Capitellidae	unclassified	Polychaeta	*Capitella capitata*
	*Notomastus*	Capitellidae	unclassified	Polychaeta	*Notomastus sp.
*Subclass Sedentaria of Polychaeta (family Chaetopteridae and Capitellidae, closer related to Capitellidae)	*Polydora*	Spionidae	Spionida	Polychaeta	*Polydora sp.
Other Polychaeta, excluding Sedentaria	Undetermined	Undetermined	Undetermined	Polychaeta	Polychaeta sedentaria
Other Arachnida (no *Ixodes* genera)	Undetermined	Undetermined	Undetermined	Arachnida	Arachnida
Genera *Ixodes*	*Ixodes*	Ixodidae	Ixodida	Arachnida	*Ixodes* sp.

* Species with ambiguity issues in their possible identification, usually because there is another invertebrate on the list with a closer taxonomic relationship to their closest relative in UniProt or there is no closest relative in this database.

## Data Availability

The mass spectrometry proteomics data have been deposited to the Proteome Xchange Consortium via PRIDE partner repository [31] with the dataset identifier PXD072353.

## Supplementary Materials

The following supporting information can be downloaded at: https://www.mdpi.com/article/10.3390/ijms27219508/s1.
