# Supplementary material for: Unlocking the Power of Metaproteomics: A Novel Tool for Enhancing Shorebird Conservation and Blue-Economy Management
Source: Int J Mol Sci. 2026 Oct 24;27(21):9508. doi: 10.3390/ijms27219508 (PMC13763323; doi:10.3390/ijms27219508)
Supplement: Supplementary file 1 [file ijms-27-09508-s001.zip › Suplementary_material_5_krona_output_Uniprot.html]

Javascript must be enabled to view this page.

magnitude
magnitudeUnassigned

Autum\_Actitis\_hypoleucos
Autumn\_Calidris\_alba
Autumn\_Calidris\_alpina
Autumn\_Charadrius\_spp
Autumn\_Himantopus\_himantopus
Autumn\_Tringa\_nebularia
Autumn\_Tringa\_totanus
Spring\_Calidris\_alba
Spring\_Charadrius\_sp
Spring\_Recurvirostra\_avosetta
Spring\_Sternula\_albifrons

1328101311.287531190810790.099181253377877.736581173851453.327431521307835.150111035121533.59191401547829.88961214336100.988161209118296.113061342259309.379451081796150.37873

116026941.915324101101576.70391995651173.9692977114299524.570578183205068.89532695649133.55881185094076.31942257687804.837855493410432.1257664100240076.61607975974384.86087

116026941.915324101101576.70391995651173.9692977114299524.570578183205068.89532695649133.55881185094076.31942257687804.837855493410432.1257664100240076.61607975974384.86087

116026941.915324101101576.70391995651173.9692977114299524.570578183205068.89532695649133.55881185094076.31942257687804.837855493410432.1257664100240076.61607975974384.86087

116026941.915324101101576.70391995651173.9692977114299524.570578183205068.89532695649133.55881185094076.31942257687804.837855493410432.1257664100240076.61607975974384.86087

116026941.915324101101576.70391995651173.9692977114299524.570578183205068.89532695649133.55881185094076.31942257687804.837855493410432.1257664100240076.61607975974384.86087

116026941.915324101101576.70391995651173.9692977114299524.570578183205068.89532695649133.55881185094076.31942257687804.837855493410432.1257664100240076.61607975974384.86087

116026941.915324101101576.70391995651173.9692977114299524.570578183205068.89532695649133.55881185094076.31942257687804.837855493410432.1257664100240076.61607975974384.86087

883492.2067209681171726.317744341459839.46468567403142.900103134458631.1914485591835598.1533385253.053862282400631.661601841704740.8056006262060280.894576295435380.51601069

580804.4368474483.732240558192213.7544184393.794986817194176.2153220464.291065849157665.4052262047.250752353588108.0536954151559605.27943683

580804.4368474483.732240558192213.7544184393.794986817194176.2153220464.291065849157665.4052262047.250752353588108.0536954151559605.27943683

580804.4368474483.732240558192213.7544184393.794986817194176.2153220464.291065849157665.4052262047.250752353588108.0536954151559605.27943683

580804.4368474483.732240558192213.7544184393.794986817194176.2153220464.291065849157665.4052262047.250752353588108.0536954151559605.27943683

580804.4368474483.732240558192213.7544184393.794986817194176.2153220464.291065849157665.4052262047.250752353588108.0536954151559605.27943683

580804.4368474483.732240558192213.7544184393.794986817194176.2153220464.291065849157665.4052262047.250752353588108.0536954151559605.27943683

302687.769920968697242.5855037861285369.80211064195681.209274237.3964617421641421.938164788.762796433242966.25640184442693.5548482731460653.815053823875775.23657386

302687.769920968697242.5855037861285369.80211064195681.209274237.3964617421641421.938164788.762796433242966.25640184442693.5548482731460653.815053823875775.23657386

302687.769920968697242.5855037861285369.80211064195681.209274237.3964617421641421.938164788.762796433242966.25640184442693.5548482731460653.815053823875775.23657386

302687.769920968697242.5855037861285369.80211064195681.209274237.3964617421641421.938164788.762796433242966.25640184442693.5548482731460653.815053823875775.23657386

302687.769920968697242.5855037861285369.80211064195681.209274237.3964617421641421.938164788.762796433242966.25640184442693.5548482731460653.815053823875775.23657386

302687.769920968697242.5855037861285369.80211064195681.209274237.3964617421641421.938164788.762796433242966.25640184442693.5548482731460653.815053823875775.23657386

174469.66257503215247.936703133911519.0258270556

174469.66257503215247.936703133911519.0258270556

174469.66257503215247.936703133911519.0258270556

174469.66257503215247.936703133911519.0258270556

174469.66257503215247.936703133911519.0258270556

174469.66257503215247.936703133911519.0258270556

1211190877.165481088537487.077521156266864.30261059148785.856751337644135.06333937636801.8797911216068500.516311156247664.48871115003123.181691239958951.868791000386385.00185

605918.865929749221179.05108389831562.501309386875777.0233020473811323.0028256293.57083953628000.830154743365639.2553485111072522.74462413

182181.581716749221179.05108389116893.290607372875777.0233020473811323.0028256293.57083953165332.167854742365639.255348511962479.324404567

182181.581716749221179.05108389116893.290607372875777.0233020473811323.0028256293.57083953165332.167854742365639.255348511962479.324404567

182181.581716749221179.05108389116893.290607372875777.0233020473811323.0028256293.57083953165332.167854742365639.255348511962479.324404567

182181.581716749221179.05108389116893.290607372875777.0233020473811323.0028256293.57083953165332.167854742365639.255348511962479.324404567

182181.581716749221179.05108389116893.290607372875777.0233020473811323.0028256293.57083953165332.167854742365639.255348511962479.324404567

423737.284213714669.210702014462668.6623110043.420219564

423737.284213714669.210702014462668.6623110043.420219564

423737.284213714669.210702014462668.6623110043.420219564

423737.284213714669.210702014462668.6623110043.420219564

423737.284213714669.210702014462668.6623110043.420219564

6397108.4583068196028.0365907410044790.82489569507898.630592757456609.4403377612200637.88487519717.3270641114370268.53086411409082.527764136851448.82521658541804.1393087

6139384.088026498196028.0365907410044790.82489569322758.122992757456609.4403377612200637.88487519717.3270641114200842.90126411409082.527764136001056.68758757842836.98609438

6139384.088026498196028.0365907410044790.82489569322758.122992757456609.4403377612200637.88487519717.3270641114200842.90126411409082.527764136001056.68758757842836.98609438

6139384.088026498196028.0365907410044790.82489569322758.122992757456609.4403377612200637.88487519717.3270641114200842.90126411409082.527764136001056.68758757842836.98609438

6139384.088026498196028.0365907410044790.82489569322758.122992757456609.4403377612200637.88487519717.3270641114200842.90126411409082.527764136001056.68758757842836.98609438

147501.9289184882.50181039476.76018147477997.647890097735582.02933738

2973058.57751537188869.511554798489407.276745118049415.689529196376376.630767247929298.672844850115.908275261725440.246347397500837.411941934885404.8684585100467.85637953

3166325.510511191007158.525035951555383.548150491273342.43346356932730.8806705224086456.710162669601.4187888512475402.65491662868768.35564071637654.1712394752006787.10037746

160528.301829004

160528.301829004

160528.301829004

160528.301829004

160528.301829004

257724.370279518185140.5076169425.6296689863.8358698967.15321433

257724.370279518185140.5076169425.6296689863.8358698967.15321433

257724.370279518185140.5076169425.6296689863.8358698967.15321433

257724.370279518185140.5076169425.6296689863.8358698967.15321433

257724.370279518185140.5076169425.6296689863.8358698967.15321433

3091076.475792641977753.635701993672991.200665383199447.269644552019435.561962222637619.92312385864.97466641903533.956752413907931.971378694609673.728841445126066.63980527

3091076.475792641977753.635701993672991.200665383199447.269644552019435.561962222637619.92312385864.97466641903533.956752413907931.971378694609673.728841445126066.63980527

3091076.475792641977753.635701993672991.200665383199447.269644552019435.561962222637619.92312385864.97466641903533.956752413907931.971378694609673.728841445126066.63980527

865807.70303688211559.083009386934592.546067558127931.959057206175634.6511441210.420020959767437.452211237

865807.70303688211559.083009386934592.546067558127931.959057206175634.6511441210.420020959767437.452211237

865807.70303688211559.083009386934592.546067558127931.959057206175634.6511441210.420020959767437.452211237

2225268.772755761766194.55269263672991.200665382264854.723576991891503.602905022461985.2722385864.97466641903533.956752413466721.551357733842236.276630215126066.63980527

2225268.772755761766194.55269263672991.200665382264854.723576991891503.602905022461985.2722385864.97466641903533.956752413466721.551357733842236.276630215126066.63980527

2225268.772755761766194.55269263672991.200665382264854.723576991891503.602905022461985.2722385864.97466641903533.956752413466721.551357733842236.276630215126066.63980527

889748713.080253815119261.844103785833027.489385768380607.0938914537119.362349667617431.11266750371191.783897900380423.318333814534353.033346941647390.546864715897766.623698

4524232.801321355218840.019156034983571.714369885580183.430567715019746.303761887837734.476974484011.038006742822361.6876509512597587.03662513069060.4320541436208564.4332096

4524232.801321355218840.019156034983571.714369885580183.430567715019746.303761887837734.476974484011.038006742822361.6876509512597587.03662513069060.4320541436208564.4332096

4524232.801321355218840.019156034983571.714369885580183.430567715019746.303761887837734.476974484011.038006742822361.6876509512597587.03662513069060.4320541436208564.4332096

4524232.801321355218840.019156034983571.714369885580183.430567715019746.303761887837734.476974484011.038006742822361.6876509512597587.03662513069060.4320541436208564.4332096

610280.57662416809.123941011502177.244963751560166.49273481949283.24440252991964.8451417936.4891439259652.00211576343247015.76365121232759.581641017468599.850922458

3642698.165740591960926.267064243005816.402749673119237.098304412897491.517438284501572.00072732938.292588032762709.685535198987909.941557812395517.5047760435367988.5131948

281385.92771166187115.719201311406261.7292898752668.29787333136.256274796211642.015019278200068.1121

305795.522033862172971.541921094240715.233537085

271254.058980761559718.700439121288462.347455151188722.588204773291529.3334151019.316396778371976.06909236

45716201.65510659709129.0785074316196500.19755968089392.316139688328021.726905225358746.25585098998.370074244167858.788760494546538.611507787883982.029273654567772.03600744

45716201.65510659709129.0785074316196500.19755968089392.316139688328021.726905225358746.25585098998.370074244167858.788760494546538.611507787883982.029273654567772.03600744

45716201.65510659709129.0785074316196500.19755968089392.316139688328021.726905225358746.25585098998.370074244167858.788760494546538.611507787883982.029273654567772.03600744

17528398.50493242398157.417730143045981.099385413133843.403139681570052.133328032225098.4641787431.033224961334396.559760491699427.788966731088407.040209183066559.2382041

17528398.50493242398157.417730143045981.099385413133843.403139681570052.133328032225098.4641787431.033224961334396.559760491699427.788966731088407.040209183066559.2382041

28187803.1501747310971.6607772913150519.09817424955548.9136757969.593577193133647.79183311567.336849282833462.2292847110.822541056795574.989064471501212.79780334

28187803.1501747310971.6607772913150519.09817424955548.9136757969.593577193133647.79183311567.336849282833462.2292847110.822541056795574.989064471501212.79780334

16900203.691670614481649.110654216331149.89038662948743.0497827923538402.55799763286653.783915974218.6578839941460.280815822352.936986317802725.95397484589033.56317691

16900203.691670614481649.110654216331149.89038662948743.0497827923538402.55799763286653.783915974218.6578839941460.280815822352.936986317802725.95397484589033.56317691

16900203.691670614481649.110654216331149.89038662948743.0497827923538402.55799763286653.783915974218.6578839941460.280815822352.936986317802725.95397484589033.56317691

16900203.691670614481649.110654216331149.89038662948743.0497827923538402.55799763286653.783915974218.6578839941460.280815822352.936986317802725.95397484589033.56317691

16900203.691670614481649.110654216331149.89038662948743.0497827923538402.55799763286653.783915974218.6578839941460.280815822352.936986317802725.95397484589033.56317691

134914292.524189163221510.845295152639529.935475156609310.238406154870513.753749115580769.76385171087399.55156586005343.4307649157705523.56162284595125.408374574026281.5736642

51038766.248795210103980.864794812880172.927737517009692.699760712767310.237784210735615.7166212936099.121444434743829.277182812075444.18014496882597.160250325777145.37576818

3808608.612431613778531.803970665473045.2607020910054994.44402645170184.543345833998236.40576881268.1508965332024806.25124586026688.380121183952139.70620821686024.12971628

3808608.612431613778531.803970665473045.2607020910054994.44402645170184.543345833998236.40576881268.1508965332024806.25124586026688.380121183952139.70620821686024.12971628

3808608.612431613778531.803970665473045.2607020910054994.44402645170184.543345833998236.40576881268.1508965332024806.25124586026688.380121183952139.70620821686024.12971628

47230157.63636366325449.060824127407127.667035436954698.255734347597125.694438336737379.310926054830.970547842719023.025936946048755.800023672930457.454042124091121.2460519

47230157.63636366325449.060824127407127.667035436954698.255734347597125.694438336737379.310926054830.970547842719023.025936946048755.800023672930457.454042124091121.2460519

47230157.63636366325449.060824127407127.667035436954698.255734347597125.694438336737379.310926054830.970547842719023.025936946048755.800023672930457.454042124091121.2460519

83875526.2753942153117529.980501139759357.007738139599617.538646142103203.515965104845154.04723158151300.43012151261514.1535821145630079.38147777712528.248124268249136.197896

2081019.598316472085893.355705092702364.752411852684341.850557972009456.88423341024620.4021693854.141693463308732.0651945647.32481089665426.24076237181.6635331

2081019.598316472085893.355705092702364.752411852684341.850557972009456.88423341024620.4021693854.141693463308732.0651945647.32481089665426.24076237181.6635331

2081019.598316472085893.355705092702364.752411852684341.850557972009456.88423341024620.4021693854.141693463308732.0651945647.32481089665426.24076237181.6635331

3474388.27044084

3474388.27044084

3474388.27044084

1417883.103447592814657.343521243270842.769009971300280.55158691779321.5354548811930729.961528596.98795448713304.304845407674340.8030764091074168.500327052868060.63611685

1417883.103447592814657.343521243270842.769009971300280.55158691779321.5354548811930729.961528596.98795448713304.304845407674340.8030764091074168.500327052868060.63611685

1417883.103447592814657.343521243270842.769009971300280.55158691779321.5354548811930729.961528596.98795448713304.304845407674340.8030764091074168.500327052868060.63611685

3845846.650588427376909.020608382161787.53454783861945.25651930379.70717381806525.91461772196.471164293667883.2041003546.6161488620.833763791018885.77326974

3845846.650588427376909.020608382161787.53454783861945.25651930379.70717381806525.91461772196.471164293667883.2041003546.6161488620.833763791018885.77326974

3845846.650588427376909.020608382161787.53454783861945.25651930379.70717381806525.91461772196.471164293667883.2041003546.6161488620.833763791018885.77326974

1643278.69857677761592.713121243735459.337443636560536.23969312614531.817427451932606.3694677940.9978650161646144.40393691250939.110712954418541.796936719581291.876849639

1643278.69857677761592.713121243735459.337443636560536.23969312614531.817427451932606.3694677940.9978650161646144.40393691250939.110712954418541.796936719581291.876849639

1643278.69857677761592.713121243735459.337443636560536.23969312614531.817427451932606.3694677940.9978650161646144.40393691250939.110712954418541.796936719581291.876849639

60011623.712973123967269.776042114862925.171912100945192.321639126457853.9345590559691.04431142704353.73608233598754.3959772111505405.97615444249165.475062246566318.5373327

44171275.8140463103106536.63463495992417.170574685175508.2216895103659753.89093782150704.65821121930356.00176313725972.745376101421025.64081830341718.341203135351694.6217274

44171275.8140463103106536.63463495992417.170574685175508.2216895103659753.89093782150704.65821121930356.00176313725972.745376101421025.64081830341718.341203135351694.6217274

7266896.8041389214810455.290003310335809.96846336025041.695315915910544.19084616583640.619913921026.270085316863779.82308155172002.397936753814140.927752724772074.81346497

7266896.8041389214810455.290003310335809.96846336025041.695315915910544.19084616583640.619913921026.270085316863779.82308155172002.397936753814140.927752724772074.81346497

7158804.564489485170357.349133097758590.4947801583.229808435379180.65874198721581.69825513891.492022972491810.6081858043.761646319505282.583394985273886.977

7158804.564489485170357.349133097758590.4947801583.229808435379180.65874198721581.69825513891.492022972491810.6081858043.761646319505282.583394985273886.977

1414646.5302983879920.502272013776107.5388744051943059.174825511508375.194024821103764.0681339079.97221049517191.2195196783054334.17575206588023.6227113971168662.12514037

1414646.5302983879920.502272013776107.5388744051943059.174825511508375.194024821103764.0681339079.97221049517191.2195196783054334.17575206588023.6227113971168662.12514037

14875874.511491916111207.771502516025977.44241233247321.318668210311659.63712589590980.356929774358.095361484852307.5093817730250199.550723529816605.401334410977397.710794

14875874.511491916111207.771502516025977.44241233247321.318668210311659.63712589590980.356929774358.095361484852307.5093817730250199.550723529816605.401334410977397.710794

874003.940212366591571.351856611339469.8149932141372160.828339102.296023851938075.369815817666576.650916978

124955.934032793119255.227174147287938.54640310457189.7596094245103730.584162323169475.309165490.442246415769659.2699210462.15802078958109.883023964

13876914.637246715400381.192471715738038.896008933190131.55905879868459.237970278049344.219929269765.357091224082648.2394817729312124.180907729606143.24331369352711.17685307

2253205.553504057203911.149099834638531.612182352862202.67042144708713.557988538293199.787610050091.39586232523025.323391243657933.278488512841947.547323234540733.57047245

2253205.553504057203911.149099834638531.612182352862202.67042144708713.557988537767701.037210050091.39586232523025.323391243657933.278488512117733.046523234540733.57047245

2253205.553504057203911.149099834638531.612182352862202.67042144708713.557988537767701.037210050091.39586232523025.323391243657933.278488512117733.046523234540733.57047245

2253205.553504057203911.149099834638531.612182352862202.67042144708713.557988537767701.037210050091.39586232523025.323391243657933.278488512117733.046523234540733.57047245

2253205.553504057203911.149099834638531.612182352862202.67042144708713.557988537767701.037210050091.39586232523025.323391243657933.278488512117733.046523234540733.57047245

525498.7504724214.5008

525498.7504724214.5008

525498.7504724214.5008

525498.7504724214.5008

541727946.833826449694152.398464442969244.900751457276105.883275556395278.102159401467114.5739399716189.27173495844293.678912459295362.179362416427973.668541446869543.816408

2268825.34125996624980.0535339023104691.99192786757448.3484715632302260.836811186830763.46863559207.337671261.83041560138.46000113186272.004766183995173.79276313

2268825.34125996624980.0535339023104691.99192786757448.3484715632302260.836811186830763.46863559207.337671261.83041560138.46000113186272.004766183995173.79276313

2268825.34125996624980.0535339023104691.99192786757448.3484715632302260.836811186830763.46863559207.337671261.83041560138.46000113186272.004766183995173.79276313

2268825.34125996624980.0535339023104691.99192786757448.3484715632302260.836811186830763.46863559207.337671261.83041560138.46000113186272.004766183995173.79276313

24687710.130775276656.190072152258942.932154625606492.308571632666218.7281038442976915.96182503315.720208251351533.3141176615782451.931178612358570.11599655451219.07009623

24687710.130775276656.190072152258942.932154625606492.308571632666218.7281038442976915.96182503315.720208251351533.3141176615782451.931178612358570.11599655451219.07009623

10212138.04277184366022.888328781593195.25232365088842.051993912307611.565767513320436.58372012802.0607603970686.14728246615620634.319577811719055.77857885451219.07009623

10212138.04277184366022.888328781593195.25232365088842.051993912307611.565767513320436.58372012802.0607603970686.14728246615620634.319577811719055.77857885451219.07009623

14475572.0879981910633.301743362665747.679831025132500.87470266358607.16233633339498030.6447490513.659447958250285.45434728792048.9845237097418255.82391959

14475572.0879981910633.301743362665747.679831025132500.87470266358607.16233633339498030.6447490513.659447958250285.45434728792048.9845237097418255.82391959

385149.381875061158448.7334130561.71248790369768.6270770414221258.513498066

385149.381875061158448.7334130561.71248790369768.6270770414221258.513498066

13436079.38708878359137.810440068197900.5494468925449688.25534117414408.5940583910707172.799517293452.792238215600373.317939339644090.724618522938897.43818778423476.59105208

13436079.38708878359137.810440068197900.5494468925449688.25534117414408.5940583910707172.799517293452.792238215600373.317939339644090.724618522938897.43818778423476.59105208

13436079.38708878359137.810440068197900.5494468925449688.25534117414408.5940583910707172.799517293452.792238215600373.317939339644090.724618522938897.43818778423476.59105208

341307.892804146650391.326875248670991.500418673508.30566981359345.88558946214863.85511898555.80698248473885.67498391133855966.98318217738975.7047479525934.648940159

1291981.242220171680175.734087232408183.375008392174625.207849991609393.021921142007902.8211393075.782642731835155.611046331506491.97002377806095.071807732576126.875745521

258889.080125668600269.73084831639871.49116484968224.0425958331334188.314594784.28261870643.94303868

4805312.121385942769312.16844482227146.330404532845589.921840682161289.561676133886827.48933055680.077557162696547.749309063905575.086662491143417.871600596805842.99675638

121005.985045293717948.489676837308208.9267105019.89076135785389.67385299317.55356637993006.3548424956

138971.39612758955211.1049584644737412.399161386520468.0865376056.684750244115773.515792745

89845.908139480390854.29606515572.069610021

6876472.145633192143449.615102691838043.6729877873661.0388930841316156.082275832566678.2637646823.5714894671170984.97635758

9346081.942753471318235.954455721678335.395940274455993.309307932908542.14175791897072.73545071792.87140798812777.5842267046001278.386821276142850.431893929738859.090086

1116351.04252894227363.223693819715723.1506842029.3416130561.712487903405830.255292853959732.732818617376807.874388194

1116351.04252894227363.223693819715723.1506842029.3416130561.712487903405830.255292853959732.732818617376807.874388194

1116351.04252894227363.223693819715723.1506842029.3416130561.712487903405830.255292853959732.732818617376807.874388194

344705.770544018763075.393029778264794.3491013041475397.97215576409925.43592112745.85486085385825.705744257158951.5231532182.50683403

344705.770544018763075.393029778264794.3491013041475397.97215576409925.43592112745.85486085385825.705744257158951.5231532182.50683403

344705.770544018763075.393029778264794.3491013041475397.97215576409925.43592112745.85486085385825.705744257158951.5231532182.50683403

179746.8494757

179746.8494757

179746.8494757

1429780.67349197173622.98234908290186.9037161237.5308164612.6318140565.6195

1429780.67349197173622.98234908290186.9037161237.5308164612.6318140565.6195

1429780.67349197173622.98234908290186.9037161237.5308164612.6318140565.6195

6455244.456188531090872.73076191915260.0029104923122105.977781851433144.16960214354931.05422959047.01654713520978.3409388015209622.425784164859553.5441752928689303.0893638

6455244.456188531090872.73076191915260.0029104923122105.977781851433144.16960214354931.05422959047.01654713520978.3409388015209622.425784164859553.5441752928689303.0893638

6455244.456188531090872.73076191915260.0029104923122105.977781851433144.16960214354931.05422959047.01654713520978.3409388015209622.425784164859553.5441752928689303.0893638

50415.1154971067129001.038436912218333.887790812373114.556985312156390.20325736475078.23164878884217.318138361

50415.1154971067129001.038436912218333.887790812373114.556985312156390.20325736475078.23164878884217.318138361

50415.1154971067129001.038436912218333.887790812373114.556985312156390.20325736475078.23164878884217.318138361

50415.1154971067129001.038436912218333.887790812373114.556985312156390.20325736475078.23164878884217.318138361

124546423.096298100252554.53190375770290.494084293560502.215679870663371.536454357202633.0276567652963.278051680984026.312892683593707.686673981891446.74801363628042.6698492

16061202.788394126162438.20118738232551.1330812415430379.57220164644704.747361983162067.334665765.7908047422990989.699269726517950.750724911287236.815903529767932.5300952

15651778.862403525032315.72960668051279.7933741115022455.18056414273085.657194423162067.334359364.9960700322516320.808769723278666.368333310159668.836727429767932.5300952

15651778.862403525032315.72960668051279.7933741115022455.18056414273085.657194423162067.334359364.9960700322516320.808769723278666.368333310159668.836727429767932.5300952

409423.9259906021130122.4715807181271.339707134407924.391637527371619.090167564306400.794734716474668.89053239284.382391621127567.97917618

409423.9259906021130122.4715807181271.339707134407924.391637527371619.090167564306400.794734716474668.89053239284.382391621127567.97917618

58718879.50255634372704.141322232183207.98376731736009.383947837018191.443884624298416.4453236430807.466648817268746.769394213554498.5540415336408.785864315420953.4930703

758734.504866563728681.081595228598941.36575674627789.710100091554449.590628291691764.5113378484.6155144341263205.45267711551228.0116439372005342.94031316521444.755479418

758734.504866563728681.081595228598941.36575674627789.710100091554449.590628291691764.5113378484.6155144341263205.45267711551228.0116439372005342.94031316521444.755479418

44127076.322046416743934.956729217096103.61403589743043.2524760519578024.31863975829305.8163219639320.98894824164309.835046426399435.429266756190498.06661265379494.96239624

44127076.322046416743934.956729217096103.61403589743043.2524760519578024.31863975829305.8163219639320.98894824164309.835046426399435.429266756190498.06661265379494.96239624

7930389.151010358180028.485611778793078.1259529216847574.73942117132566.1028447715253284.47766206097.006245311299542.14493192306297.869989594128956.22485322944232.45355936

7930389.151010358180028.485611778793078.1259529216847574.73942117132566.1028447715253284.47766206097.006245311299542.14493192306297.869989594128956.22485322944232.45355936

5902679.52463278720059.617386055695084.878021524517601.68195069753151.43177192524061.640110206904.8559409541689.3367387974297537.243139763011611.554085336575781.32163532

5902679.52463278720059.617386055695084.878021524517601.68195069753151.43177192524061.640110206904.8559409541689.3367387974297537.243139763011611.554085336575781.32163532

9298.63826885324

9298.63826885324

9298.63826885324

2367642.084451292624948.224198661709401.669530041811756.432222352048342.130268611745340.25991405386.2842066715038494.7264282942184.3449247033271739.84468835453812.781526369

2367642.084451292624948.224198661709401.669530041811756.432222352048342.130268611745340.25991405386.2842066715038494.7264282942184.3449247033271739.84468835453812.781526369

2367642.084451292624948.224198661709401.669530041811756.432222352048342.130268611745340.25991405386.2842066715038494.7264282942184.3449247033271739.84468835453812.781526369

1009100.00555157613366.6691492320.701214716239689.1352737677.4085400641969346.6911575674.95417137563629.798239811336366.352918706560173.161739701

1009100.00555157613366.6691492320.701214716239689.1352737677.4085400641969346.6911575674.95417137563629.798239811336366.352918706560173.161739701

1009100.00555157613366.6691492320.701214716239689.1352737677.4085400641969346.6911575674.95417137563629.798239811336366.352918706560173.161739701

32020978.815908519873175.851737918488961.836994631944518.757895914740081.600541717663746.5191711682741.61942812780465.406819631987815.400134345250014.579928512264967.9498366

207621.343980688561345.048372786557797.085768328770854.63841033414.11470346

207621.343980688561345.048372786557797.085768328770854.63841033414.11470346

8090798.333036748143444.5552476211492874.6766258672031.479447377367167.30823116205339.12267292941.213675872431080.7731088818204175.708825213903056.37551895918610.69660724

8090798.333036748143444.5552476211492874.6766258672031.479447377367167.30823116205339.12267292941.213675872431080.7731088818204175.708825213903056.37551895918610.69660724

23722559.138891111729731.29649036434742.1119967622714690.19268027372914.2923106310687552.758174389800.405752110349384.633710812750225.576605631346958.20440966346357.25322939

23722559.138891111729731.29649036434742.1119967622714690.19268027372914.2923106310687552.758174389800.405752110349384.633710812750225.576605631346958.20440966346357.25322939

952903.777790863363863.046115327109949.0629644691543692.70324848163841.08031773385.24573802244033.630682861487318.9295777236.757166535

952903.777790863363863.046115327109949.0629644691543692.70324848163841.08031773385.24573802244033.630682861487318.9295777236.757166535

952903.777790863363863.046115327109949.0629644691543692.70324848163841.08031773385.24573802244033.630682861487318.9295777236.757166535

816359.3915084798.68983164607478.203108166522330.713503554679468.22978646852772.9163508892.432276991318995.140622172451075.0352731266809.668786221616373.70630652

816359.3915084798.68983164607478.203108166522330.713503554679468.22978646852772.9163508892.432276991318995.140622172451075.0352731266809.668786221616373.70630652

816359.3915084798.68983164607478.203108166522330.713503554679468.22978646852772.9163508892.432276991318995.140622172451075.0352731266809.668786221616373.70630652

12590058.092376511521122.75452589692505.920273235765869.157744125251213.272822371347101.785966610309.4847770411022704.77211897559783.617975524431744.961602423326765.4518476

246456.113664959199500.811232002

246456.113664959199500.811232002

12590058.092376511274666.64086099692505.920273235765869.157744125251213.272822371347101.785966410808.6735450411022704.77211897559783.617975524431744.961602423326765.4518476

12590058.092376511274666.64086099692505.920273235765869.157744125251213.272822371347101.785966410808.6735450411022704.77211897559783.617975524431744.961602423326765.4518476

174321.62773409578239.0115014586168520.9889274177.351162342.912291118203512.441574763

174321.62773409578239.0115014586168520.9889274177.351162342.912291118203512.441574763

174321.62773409578239.0115014586168520.9889274177.351162342.912291118203512.441574763

174321.62773409578239.0115014586168520.9889274177.351162342.912291118203512.441574763

59353272.558057866894256.415799270491597.7353816108614158.149185117475497.87530870384647.7384286228427.178986497090853.339598264872427.26300243681340.11076580912301.7563578

14503812.980644122964887.866296925517824.494307932796090.366338520037233.992627320768192.316525767170.284414248119090.049533729986437.895391818849262.914289617022331.0068884

5446176.056250536113523.672052434606211.172939992732059.554603085941339.051166353105066.68383368119.173824512029436.45449665268942.288624561571586.817212963513131.18022648

5446176.056250536113523.672052434606211.172939992732059.554603085941339.051166353105066.68383368119.173824512029436.45449665268942.288624561571586.817212963513131.18022648

993333.3725229633957377.530079043322444.0905214518786133.94164452926712.63658771832175.93722756272.26213938846626.9709167641499810.63435671362875.5851032981446334.66950677

993333.3725229633957377.530079043322444.0905214518786133.94164452926712.63658771832175.93722756272.26213938846626.9709167641499810.63435671362875.5851032981446334.66950677

2101741.664349638594587.207835939188453.044477078105509.9111077835187.259455211426121.772315763698.925666617688317.393956413323849.252420710957187.14703912876616.66623535

796082.9700769031321926.814111211225677.155718031756750.578586613105907.38145527314553.48764132884.158071181778906.6083132510095345.0577771443271.871679504412793.09548947

1305658.694272727272660.393724737962775.888759046348759.332520394729279.8784111568.284711630814.767595415909410.78564323228504.194643510513915.27535962463823.57074588

1297228.549744021813194.108866112731807.336256081729468.623321132269561.473295941632287.51691939585.5519282215909764.18440027911936.938796254406114.208240764984058.36920734

1297228.549744021813194.108866112731807.336256081729468.623321132269561.473295941632287.51691939585.5519282215909764.18440027911936.938796254406114.208240764984058.36920734

4521647.370189171519328.428388255245568.890504241369826.187182781064433.572122072488673.95961768772.158670991270535.836423991745841.272547251551499.156693554011875.15546427

1125479.608645581519328.428388255038237.708297071341586.452062981064433.572122072342277.79831768772.158670991270535.836423991745841.272547251551499.156693554011875.15546427

3396167.76154358207331.1822071728239.7351198043146396.1613

143685.967587772966876.91907517423339.95960912473092.14848283866.4467170722.212184514374409.209339804236057.508646413190314.96624814

143685.967587772966876.91907517423339.95960912473092.14848283866.4467170722.212184514374409.209339804236057.508646413190314.96624814

262334.393418325602295.2574019121514629.35034907337899.505778608942464.917730123708603.8536683299.6817993424874401.81534187171094.431896546

262334.393418325602295.2574019121514629.35034907337899.505778608942464.917730123708603.8536683299.6817993424874401.81534187171094.431896546

262334.393418325602295.2574019121514629.35034907337899.505778608942464.917730123708603.8536683299.6817993424874401.81534187171094.431896546

4822038.234596292652366.03383073412263.6319788323377326.789747952553816.21073082258299.570624745017.85250811081692.886641333620854.349482241663517.2875721423488472.7048843

4058695.916176442335582.928706382650133.8785728622172767.229892651792982.15340151672066.860524321113.0142521789009.7151034451896128.72048224690165.53520300222137354.237882

4058695.916176442335582.928706382650133.8785728622172767.229892651792982.15340151672066.860524321113.0142521789009.7151034451896128.72048224690165.53520300222137354.237882

763342.318419854242687.261635265561676.7908041421204559.55985528608805.70524075103887.8365423904.838255989212167.5653778861724725.629465939.7767513111351118.46700236

763342.318419854242687.261635265561676.7908041421204559.55985528608805.70524075103887.8365423904.838255989212167.5653778861724725.629465939.7767513111351118.46700236

74095.8434890559200452.962601828152028.352088572482344.873680515.60616507411.975617824

74095.8434890559200452.962601828152028.352088572482344.873680515.60616507411.975617824

393477.526489917562333.4998839691030586.61695839524922.2329923347.486551453380447.6370211733557925.31881623200798.2077488751494341.704446393858862.31805669

393477.526489917562333.4998839691030586.61695839524922.2329923347.486551453380447.6370211733557925.31881623200798.2077488751494341.704446393858862.31805669

393477.526489917562333.4998839691030586.61695839524922.2329923347.486551453380447.6370211733557925.31881623200798.2077488751494341.704446393858862.31805669

405363.206493657175379.5768365639.255348511534800.8806

405363.206493657175379.5768365639.255348511534800.8806

405363.206493657175379.5768365639.255348511534800.8806

356798.728106497408446.983530129870921.8051467341488264.86634462201836.1317341461157398.80282199587.133963824184544.0364415241415920.00190551051503.053145111129150.17563973

356798.728106497408446.983530129870921.8051467341488264.86634462201836.1317341461157398.80282199587.133963824184544.0364415241415920.00190551051503.053145111129150.17563973

356798.728106497408446.983530129870921.8051467341488264.86634462201836.1317341461157398.80282199587.133963824184544.0364415241415920.00190551051503.053145111129150.17563973

3827729.356788184920518.002493621471317.636291943313368.394476871874021.804264663623851.8962202623.9957505153560693.929358851835873.377516341585590.56369383864727.056965523

3827729.356788184920518.002493621471317.636291943313368.394476871874021.804264663623851.8962202623.9957505153560693.929358851835873.377516341585590.56369383864727.056965523

3263317.202413023780662.77957386526676.3836735762263980.66463515429531.3633787281964298.6918332000.4162633200.7842119791061051.62582986506232.157829633

462258.581854451950269.721201162758800.992743623933733.5795058161252166.1293228693.513158851038619.41637211407465.63746397358494.89913589

102153.572520706189585.501718598185840.259874742115654.1503359121444490.44088593407387.0754202623.995750515164053.176932254117073.3004

13904754.201679621580713.123877416760394.538278314841582.911609920975938.204818116359060.569318357379.053804319671610.418650910144151.61914918583195.6160037114767872.3029033

9885554.8016062319067855.515954914280373.541660210758069.081797817853401.465050414945860.779815258046.733061114528316.43568036938900.102571286356322.138887237787158.65724676

9885554.8016062319067855.515954914280373.541660210758069.081797817853401.465050414945860.779815258046.733061114528316.43568036938900.102571286356322.138887237787158.65724676

96804.213197244454631.13937542182159.3540864342954424.324376413142922.28425515487491.9274142348.582613598

96804.213197244454631.13937542182159.3540864342954424.324376413142922.28425515487491.9274142348.582613598

826411.46894589681362.718447203816565.75380017422248.923964151966746.84184432558856.07011859418.888189931698824.63629058379324.603429183501145.40245129812.846

826411.46894589681362.718447203816565.75380017422248.923964151966746.84184432558856.07011859418.888189931698824.63629058379324.603429183501145.40245129812.846

271746.513927751227876.285578602108813.753471533111326.485875656183015.625351304879881.935710005123718.822215494459000.505321213426201.244494754

271746.513927751227876.285578602108813.753471533111326.485875656183015.625351304879881.935710005123718.822215494459000.505321213426201.244494754

165143.66412393226900.317570057112136.2093120657.483102433364783.415313419143069.364881668

165143.66412393226900.317570057112136.2093120657.483102433364783.415313419143069.364881668

2824237.204002511148987.464521221416152.224607552598026.828674640.810222495654715.5827936240.324099532199803.995656612702208.09093318981309.62191424699.55516178

2824237.204002511148987.464521221416152.224607552598026.828674640.810222495654715.5827936240.324099532199803.995656612702208.09093318981309.62191424699.55516178

21282327.136334913202695.648484619913659.662070431529339.87549519966839.126851225333861.152615892901.53972516040894.884813817302752.55645969748033.6591176819780886.1910199

5605074.798023694322399.005462754156841.077164074941397.265417288209496.232616283179745.09534741225.839723282592418.605903823648066.113513061699419.012505736712914.52746001

5605074.798023694322399.005462754156841.077164074941397.265417288209496.232616283179745.09534741225.839723282592418.605903823648066.113513061699419.012505736712914.52746001

3695985.361732434418495.572447576859905.965846933647603.669889255543470.647579917097039.42114308063.256701653251743.667947963383751.704557221052697.808607153085768.28069854

3695985.361732434418495.572447576859905.965846933647603.669889255543470.647579917097039.42114308063.256701653251743.667947963383751.704557221052697.808607153085768.28069854

1966297.408491025050318.78168057.502724245262495.158337432

1966297.408491025050318.78168057.502724245262495.158337432

1009440.68471201609.022467872276461.32072961312808.348457432321893.444736021622563.35222288200.568814961171683.833029361711385.400140481850810.556217835239564.6986558

516551.9642298290.4248511561009716.96869726889271.0570574321122661.07874861592549.3462859372.38148958489920.366629364895949.8114053371192653.284766844523316.43699211

492888.7205903318.5976167161266744.35203234423537.29141199232.365987411030014.0061428828.18732538681763.4664815435.588735139658157.27145099716248.261663699

671509.7472453751218928.4094898720095.946560949588097.8492116181155194.177232951979387.34561387353.314617111134767.785413431670641.621244511099272.211074382074689.44470159

671509.7472453751218928.4094898720095.946560949588097.8492116181155194.177232951979387.34561387353.314617111134767.785413431670641.621244511099272.211074382074689.44470159

54676.4604132631.19005181642199.684230500873019.70643

54676.4604132631.19005181642199.684230500873019.70643

2174313.454660042.035510989952961.765020849626739.053399714996207.907193456829154.88271745584.435189655384304.858963423264176.77400887788074.488850336517547.965440219

2174313.454660042.035510989952961.765020849626739.053399714996207.907193456829154.88271745584.435189655384304.858963423264176.77400887788074.488850336517547.965440219

260953.533692301846070.239336713326790.78451780517189371.874934699013.095383488999076.4351587788.685379165852447.474125949248306.355711348851515.953865311

260953.533692301846070.239336713326790.78451780517189371.874934699013.095383488999076.4351587788.685379165852447.474125949248306.355711348851515.953865311

4495352.491513740.658974896448468.410551762275032.499

4495352.491513740.658974896448468.410551762275032.499

3369697.06594109535151.3637689562140564.734764272720176.943233951041563.622109054576575.8406834685.4392991931653528.659429873492099.752943632524163.54019616963370.455430995

3258172.1674410899613.1042972833252982.613407488603359.667196108742111.644474823185324.3416334160.7506822911151009.588419481035389.1059422985.953491201

111524.8985435538.2594716721887582.121356782116817.27603784299451.9776342314391251.499500524.688616903502519.0710103883492099.752943631488774.43429616540384.501939794

59494640.273988249468759.466140150637226.842594352193157.972261748544196.368447158571158.3219749809736.226980292265406.633777949152652.361884151128873.234251477999284.7642443

116647.796375961885102.618796071753677.0919908551818458.300759551997277.60891365331416.04531519885.83839542844918.552585233673604.526084897876735.010977603803807.540329644

116647.796375961885102.618796071753677.0919908551818458.300759551997277.60891365331416.04531519885.83839542844918.552585233673604.526084897876735.010977603803807.540329644

116647.796375961885102.618796071753677.0919908551818458.300759551997277.60891365331416.04531519885.83839542844918.552585233673604.526084897876735.010977603803807.540329644

622608.111129152837186.4650727882285112.958494221690352.13044203775889.2469595031267140.72542849612.392853721666334.6007657672310081.23751199785596.173837656793892.496897493

470576.095247757837186.4650727882177740.296760331690352.13044203722730.4504212831220707.9144849612.392853721666334.6007657672310081.23751199755873.814608969793892.496897493

470576.095247757837186.4650727882177740.296760331690352.13044203722730.4504212831220707.9144849612.392853721666334.6007657672310081.23751199755873.814608969793892.496897493

152032.015881395107372.66173388253158.796538219946432.8110229722.3592286866

152032.015881395107372.66173388253158.796538219946432.8110229722.3592286866

349524.469171744990897.580093491650885.4323319246967.7894433189.70347919561656.87161996473841.6284857145214175.070289567

349524.469171744990897.580093491650885.4323319246967.7894433189.70347919561656.87161996473841.6284857145214175.070289567

349524.469171744990897.580093491650885.4323319246967.7894433189.70347919561656.87161996473841.6284857145214175.070289567

660501.029817601604817.491304542586297.1136336667.734341686645912.8677858516.308139706387166.8938458812247010.53533259249325.162288324

660501.029817601604817.491304542586297.1136336667.734341686645912.8677858516.308139706387166.8938458812247010.53533259249325.162288324

660501.029817601604817.491304542586297.1136336667.734341686645912.8677858516.308139706387166.8938458812247010.53533259249325.162288324

25046269.21460518644203.2170934210719426.24986147394010.946985775104977.462477518993166.9267040805.642264775127961.863502027392912.58238556822819.2632793916923482.1858711

770212.64311460611.578100561371783.69113875747125.981728689122933.2104042632634906.0861596145.12077376924365.796935391639554.025655529490510.7533

770212.64311460611.578100561371783.69113875747125.981728689122933.2104042632634906.0861596145.12077376924365.796935391639554.025655529490510.7533

1840642.424000521841088.288133344883335.411804651570532.622903451796647.724569752621685.2522321247.83461352627805.127518621794384.728800676597065.93072624311011753.94808

1840642.424000521841088.288133344883335.411804651570532.622903451796647.724569752621685.2522321247.83461352627805.127518621794384.728800676597065.93072624311011753.94808

22435414.14750465342503.350859524173179.965528244981073.277110043185396.52750353736575.5882592555.272994094266972.08337245674162.056649435586199.306897625421217.48449107

22435414.14750465342503.350859524173179.965528244981073.277110043185396.52750353736575.5882592555.272994094266972.08337245674162.056649435586199.306897625421217.48449107

291127.18138978995279.0652435802530857.4138834233184.652611002

291127.18138978995279.0652435802530857.4138834233184.652611002

12863589.297404713065670.79995998257400.544621915475800.7932045714962362.337579211137025.7074514396828.432427159668664.99668949003289.718030475480044.2678760232170542.9742533

7041725.509104041402181.286183321462492.531931951831771.540509351579806.486952020162.9530469528403304.84210952035318.13037716235504.03183511325322233.7937563

7041725.509104041402181.286183321462492.531931951831771.540509351579806.486952020162.9530469528403304.84210952035318.13037716235504.03183511325322233.7937563

1815582.481879911706785.305532262181485.516648761525761.515869223302156.178414282450517.3982068823.6691737716354710.53987135743733.515328351904587.777716765746000.18580744

1815582.481879911706785.305532262181485.516648761525761.515869223302156.178414282450517.3982068823.6691737716354710.53987135743733.515328351904587.777716765746000.18580744

4006281.306420769956704.208244334613422.49604122118267.7368259911660206.1591657106701.822510307841.810206414910649.61470861224238.072324963339952.458324141102308.99468958

4006281.306420769956704.208244334613422.49604122118267.7368259911660206.1591657106701.822510307841.810206414910649.61470861224238.072324963339952.458324141102308.99468958

15640787.918414818869437.183406217919451.77178926898157.39468921325537.137494430350396.072121925716.864485619522702.601626724010257.021818739115.953668315848823.2344406

2363899.407829021318580.281975493156131.955222931607592.376823366020854.594069521019019.33587043204.26331092786189.799237181500184.912716181520365.104292423016968.74539044

2363899.407829021318580.281975492973325.658757031393978.088847925907844.35331627692753.176793365.852643891251975.855237181500184.912716181520365.104292422764534.79002129

182806.296465892213614.287975439113010.240753251326266.1658249838.4106670191534213.944252433.955369147

4258203.601326744985042.782166497025744.820498955301238.236623944140378.064679424384453.0784536600.377612223705839.291004586550763.107768733806490.117974013772939.69173591

4258203.601326744985042.782166497025744.820498955301238.236623944140378.064679424384453.0784536600.377612223705839.291004586550763.107768733806490.117974013772939.69173591

1625228.039366973855011.56436092385035.191990423178984.228694598729424.752156419573530.8957502953.950549894077734.762884941123305.834211861457832.925957561280844.90670502

1625228.039366973855011.56436092385035.191990423178984.228694598729424.752156419573530.8957502953.950549894077734.762884941123305.834211861457832.925957561280844.90670502

5620629.033255827718085.418702874329330.6291770715318160.2052751900609.969579713948211.3232629808.479121038357634.63614111282.156863611806625.51751556678960.17391464

5620629.033255827718085.418702874329330.6291770715318160.2052751900609.969579713948211.3232629808.479121038357634.63614111282.156863611806625.51751556678960.17391464

120408.207141299403034.621210638228286.202554541676355.125273885534269.757009356167021.43213149.793891551125631.424424065147802.2879288111099109.71669463

120408.207141299403034.621210638228286.202554541676355.125273885534269.757009356167021.43213149.793891551125631.424424065147802.2879288111099109.71669463

499555.788875811286538.557778356196075.6348228955.16911155154171.4473220777.402967622

499555.788875811286538.557778356196075.6348228955.16911155154171.4473220777.402967622

1152863.8406191303143.957211468598847.337545138586872.0528866921103988.563595304.1125378312.182847944

1152863.8406191303143.957211468598847.337545138586872.0528866921103988.563595304.1125378312.182847944

837675.338583375458722.6135601934999938.45990393656697.480224618238810.420727055249219.93791117895.27244634350361.7219760007.031307965203976.8621826033144780.57425239

837675.338583375458722.6135601934999938.45990393656697.480224618238810.420727055249219.93791117895.27244634350361.7219760007.031307965203976.8621826033144780.57425239

837675.338583375458722.6135601934999938.45990393656697.480224618238810.420727055249219.93791117895.27244634350361.7219760007.031307965203976.8621826033144780.57425239

2052055.600771294354909.971936773043610.593029725099591.480927062336527.225891163075817.46251869469.921672242218154.149565831901188.9028688114890649.64744811991418.10652529

2052055.600771294354909.971936773043610.593029725099591.480927062336527.225891163075817.46251869469.921672242218154.149565831901188.9028688114890649.64744811991418.10652529

2052055.600771294354909.971936773043610.593029725099591.480927062336527.225891163075817.46251869469.921672242218154.149565831901188.9028688114890649.64744811991418.10652529

1965482.527532051693025.566497172053791.681598681582894.75133563815261.7617309572274094.7882656332.1589558553007791.839003222152487.576428671009083.891163495859037.4190965

1965482.527532051693025.566497172053791.681598681582894.75133563815261.7617309572274094.7882656332.1589558553007791.839003222152487.576428671009083.891163495859037.4190965

1965482.527532051693025.566497172053791.681598681582894.75133563815261.7617309572274094.7882656332.1589558553007791.839003222152487.576428671009083.891163495859037.4190965

103611305.386637113825177.74126394683701.243286399425948.2159864103820741.83740968048022.2748482734767.9078784102458308.218689130857890.348167100849857.751643103346275.787985

182434.836739654584328.18944483858498.103193423541310.7889761946.77641113215.634976265

182434.836739654584328.18944483858498.103193423541310.7889761946.77641113215.634976265

182434.836739654584328.18944483858498.103193423541310.7889761946.77641113215.634976265

1305929.59383676275042.424858775.119974794563903.11604765000982.67159132748048.4993597835.149472971473303.3294821735.152013541439280.569191081229817.37738207

1305929.59383676275042.424858775.119974794563903.11604765000982.67159132748048.4993597835.149472971473303.3294821735.152013541439280.569191081229817.37738207

1305929.59383676275042.424858775.119974794563903.11604765000982.67159132748048.4993597835.149472971473303.3294821735.152013541439280.569191081229817.37738207

45208809.141356437241366.608347547030224.064405437268990.638779735757200.591959628908504.3317327194460.558588828588224.017756548731507.734049220753217.526671735934318.1567594

587389.8681252031407988.516389342707786.39269332312151.901704521461765.812361921430353.28261076745.54395421644731.530513999674651.8504723291491211.14184364610770.941607725

587389.8681252031407988.516389342707786.39269332312151.901704521461765.812361921430353.28261076745.54395421644731.530513999674651.8504723291491211.14184364610770.941607725

20531608.477035616976678.90048325157964.654044220361301.060174121148247.918132413168536.8036312487304.963872615361254.559406521415662.67251869549789.1583440226663189.2236386

1523776.982322434292853.253551784175657.927043074657244.81779527986346.408767541940070.7821948448.4364851362556405.675774612.4633015864.69456245

471184.975978276560128.443070271926991.048956653526024.716909954177369.7923447456.354525268247410.072844694874104.372988012.1230269937350108.9491

667003.400886271539382.8874273992.289667695502376.239488444238451.320496968703082.481921043182177.845412152281913.472989714

2861686.600475511218320.745529512561483.616875053472819.348330168812786.67966654421176.07861103342.668863192399174.561754452853184.883883761331576.086862421314113.7068963

810108.9724464061661241.379046651522289.181016821262641.894381421139473.487806721658933.0897859738.2749129331575892.01621749618.587426251081295.862255595622909.20073491

1185337.291709411317273.578816944735518.961669451217264.156354581350452.786513421853858.1594879594.344070691291602.640625871476302.42983602577786.546703929935022.171606281

4476781.150221811836278.081289731949786.291665042566061.364257511260053.989803061211195.01421742242.851674352401075.355435464004557.513249281753354.196257221373425.78150641

8129509.376218184122970.422079994734753.878491714104295.640880264969123.092347824795912.8414129133.45896933681507.6589131978843597.288993841614286.951288263434444.42687632

497233.264591681128581.2089245489.9785207192.6751390741225682.1302100257.719016617395523.433262767

662831.2815247941363504.774690512816854.014914831125348.188420581220569.92744999464645.25351117255.901029973505104.09671181432119.750817321678543.891794789680040.01407066

410391.846138781433528.24299796509152.33263173847.914239814419693.66263921383.633828164368407.564165118541736.84502248

24089810.796195618856699.191475119164473.017667914595537.67690113147186.861465314309614.245513630410.05076212582237.92783626641193.21105829712217.226484028660357.99151314

2692473.265625384736450.829263215569124.221249214501952.481022214133840.94405943225405.9564618393.252651751225632.20896277747539.628155351661699.072674931465214.01040539

9331730.82326087225517.447736436408480.527562917837937.170255416757812.104286539793836.8616818308.689955263639023.8854810310252837.04424224376866.550394914925446.90415119

11839877.22080943653730.581443646496328.438821621455469.521651161204795.190915511290371.42852193708.108154985570983.93470378640816.538660713496523.186264252269697.07695656

225729.48653241000.33303183690539.830034158800178.503972251050738.622203822146597.89868856177128.417149929

6600185.807572761427262.583272371003583.317700214065642.018795567168.903495513131334.7609171879.679569851032681.777249592275222.72710694510368.697243919

6600185.807572761427262.583272371003583.317700214065642.018795567168.903495513131334.7609171879.679569851032681.777249592275222.72710694510368.697243919

5897772.392472761145108.85084095286396.2312420533773513.10953673131334.7609171879.67956985435971.91081806043.45132451

702413.4151282153.732431412717187.086458155292128.909258266567168.903495513596709.866449591469179.275782424510368.697243919

3315989.714787663065954.159939441922048.0428170612832153.06119634549780.199757842725096.88269156285.8516602412015617.642183916459979.71717323661194.3675211713821561.8438158

3315989.714787663065954.159939441922048.0428170612832153.06119634549780.199757842725096.88269156285.8516602412015617.642183916459979.71717323661194.3675211713821561.8438158

3315989.714787663065954.159939441922048.0428170612832153.06119634549780.199757842725096.88269156285.8516602412015617.642183916459979.71717323661194.3675211713821561.8438158

1174818.859947082090445.861906691989450.214796556735873.65122527533422.385174687128110.3268517398.636171734660478.0978692248709819.657376152073954.727257073630893.95612143

937460.3315454162090445.861906691430556.547568091181252.34754939533422.385174687128110.3268517398.636171734875730.4552468691152698.27251345676481.953786319

937460.3315454162090445.861906691430556.547568091181252.34754939533422.385174687128110.3268517398.636171734875730.4552468691152698.27251345676481.953786319

237358.528401662558893.6672284575554621.30367587660478.0978692247834089.20212928921256.454743622954412.00233511

237358.528401662558893.6672284575554621.30367587660478.0978692247834089.20212928921256.454743622954412.00233511

4336483.2863533697.940322313477648.529200254800429.4822569268.993686836229220.2362625706.616779887494385.751766711932276.598966114965194.25966003

4336483.2863533697.940322313477648.529200254800429.4822569268.993686836229220.2362625706.616779887494385.751766711932276.598966114965194.25966003

4336483.2863533697.940322313477648.529200254800429.4822569268.993686836229220.2362625706.616779887494385.751766711932276.598966114965194.25966003

7518952.222037567212114.363423967009745.26693616499678.297960668938914.330897044609770.2036969943.918062713044450.8266138818780971.807357712953300.19457164154321.55298439

975186.042871393844366.001288171930793.126753923377386.773897041367942.6331901065.547834641393032.76811342925753.630682092618216.21341328743.68906585

975186.042871393844366.001288171930793.126753923377386.773897041367942.6331901065.547834641393032.76811342925753.630682092618216.21341328743.68906585

7518952.222037566236928.320552573165379.265647934568885.171206735561527.5573241827.575068878.370228061651418.0585004517855218.176675612335083.98117162825577.86391855

7518952.222037566236928.320552573165379.265647934568885.171206735561527.5573241827.575068878.370228061651418.0585004517855218.176675612335083.98117162825577.86391855

33475217.796035349975992.890744526194173.722166321143601.909126744515637.561313519555589.758832002535.575185456676234.305265524297088.913093955498449.652091738022930.2279367

33181201.24989748968931.501500625365670.061604520729043.640581343737537.548522317856304.038831143138.619740456676234.305265523576249.039344455498449.652091738022930.2279367

1649482.78256995635289.454517511244809.554342791251112.260290171455142.79511745621.705760992358862.22690057315069.6436537484559859.418419972044039.3545603

2429757.9890985758509.588941316124060.644822894419972.691140828413713.8948331451352967.925207221122435.85466488135669.767415471173938.970453774

1447947.83078076295851.17734374244069.476720916188571.939666127135625.0454368896.795674132121958.4415417065.8529671691059048.84343055

214340.30289662456047.8505737653309866.960948377102378.1857249978.108382762147596.027406018236688.68653594837344.0040190792120536.679916236

3235114.602617473242059.193650885201447.004090072702525.9945206174.026641623493555.3865654919.973182783949292.917280841243845.87477739572345.9448755672393320.54002317

3004913.97095165184463.758162653236771.654842262646139.929092263710567.02774556537749.35657186860.42312583608.42729943349995.9077545530473416.690445617496418.4526687

2652116.057468561970463.1166252696305.018403412926532.526245111114846.43457466767158.68351683924.21944248904509.791469588.25062466874748.171948378756139.4255

3240642.165285595919573.666871948554661.477150245562115.388364878260470.4873614967531.17136280008.891119062767296.783249555524836.418315248434785.154286163571960.58259914

6881434.3596339620532244.5903482441927.2650795311050467.856495112461950.77672456920861.66540551.60312449112577773.9276747606151.333374611833206.4484950424593611.15697219

449682.349498351679448.8970809142362684.29141561781558.34364467

1030582.01309755932048.761607425858372.230279161581103.34210596848734.009915845974517.7061752808.961111635818063.68411335174.96809291878942.46306538398048.976526434

6776555.347326935493605.308268492177113.388144451513800.86582182317737.925766781179750.8331738187.135911416165728.719063022359807.852190014308849.3561578126.96766387

328601.278327902526575.696501221333209.72336768342877.767924835219706.07672325793.97539311265748.65217967

68897.56204258197.983131131134821.9171204356.56934372768477.7303574342620083.561821171

436924.980689164196890.408738988168556.332965244482741.753886764270908.29235814253.370152971777179.340911481011659.06390993

851053.0504348222173277.334645291754836.691292011950672.7309829615768269.22893472696596.93011602497.9648350310833313.53073341185166.966456841088290.105846611114187.97016534

294016.5461383371007061.38924387828503.6605618414558.268545388778100.0127912071699285.72859396.955444969720839.873749534

294016.5461383371007061.38924387828503.6605618414558.268545388778100.0127912071699285.72859396.955444969720839.873749534

492484.1283235662418972.723861511139554.862096231474365.251885191388366.199533032950400.4996498721.922386858529719.838087502262961.388266005963654.081105053

477351.882275985560012.274773735311952.149529338583532.63919421309602.343972967805230.2066529719.838087502262961.388266005963654.081105053

430936.808165985311952.149529338583532.63919421309602.343972967529719.838087502262961.388266005963654.081105053

46415.07411560012.274773735805230.2066

15132.24604758071858960.44908777827602.71256689890832.6126909841078763.855560062145170.293498721.922386858

15132.24604758071858960.44908777827602.71256689890832.6126909841078763.855560062145170.293498721.922386858

3178864.406635523098902.828488592319097.722819093483014.954767423938654.367903423860519.06973806468.33146941824904.799205111651282.617294422030869.290284151566066.33440619

3178864.406635523098902.828488592319097.722819093483014.954767423938654.367903423860519.06973806468.33146941824904.799205111651282.617294422030869.290284151566066.33440619

3178864.406635523098902.828488592319097.722819093483014.954767423938654.367903423860519.06973806468.33146941824904.799205111651282.617294422030869.290284151566066.33440619

3178864.406635523098902.828488592319097.722819093483014.954767423938654.367903423860519.06973806468.33146941824904.799205111651282.617294422030869.290284151566066.33440619

141754329.194841100272168.740196133749220.98161463342847.2770116196288271.25892180714031.8250190899667.4242524112547427.18412666179442.399720494131266.78302774808843.9595681

2374437.642350931411696.906888672376442.891119341590000.338729763157328.026790812908099.27372702088.96754981622753.159025092178961.924997614478932.09474418925.62704102

2374437.642350931411696.906888672376442.891119341590000.338729763157328.026790812908099.27372702088.96754981622753.159025092178961.924997614478932.09474418925.62704102

2374437.642350931411696.906888672376442.891119341590000.338729763157328.026790812908099.27372702088.96754981622753.159025092178961.924997614478932.09474418925.62704102

32958564.4253839668200.0533544611149410.3701557144883.9283883510141286.960213319955041.56610566527.81490069574960.936651246562837.8100910811220953.3712275994870.05450226

1733170.35354884210165.877736701951777.1841814711458958.719688583142725.290436351665130.723613222273.15809096693317.3334782431613774.961641881012272.71772282661615.96915568

1733170.35354884210165.877736701951777.1841814711458958.719688583142725.290436351665130.723613222273.15809096693317.3334782431613774.961641881012272.71772282661615.96915568

2923940.351944621863408.098471651356656.520092761046098.605420211950471.47330315998252.964092049361.837614051263165.019305841272621.60623587523791.3159429721106061.40388317

2923940.351944621863408.098471651356656.520092761046098.605420211950471.47330315998252.964092049361.837614051263165.019305841272621.60623587523791.3159429721106061.40388317

20393090.5983523970321.210070253752846.35445882942062.683818692196252.359578543605161.59312074321.556832394024819.3052585978.781959283107619.630843761770625.88884489

20393090.5983523970321.210070253752846.35445882942062.683818692196252.359578543605161.59312074321.556832394024819.3052585978.781959283107619.630843761770625.88884489

6068493.1152946182.431600634563957.448122071272107.248027752167618.57899523477313.65122688562.216917613318678.6122266745162.2686890184870105.4481353403.55031881

6068493.1152946182.431600634563957.448122071272107.248027752167618.57899523477313.65122688562.216917613318678.6122266745162.2686890184870105.4481353403.55031881

1839870.00653753678122.435475238524172.863299872425656.671433124684219.257913209182.634532009.045445582274980.666640565345300.1915650351707164.258717421103163.24229971

1839870.00653753678122.435475238524172.863299872425656.671433124684219.257913209182.634532009.045445582274980.666640565345300.1915650351707164.258717421103163.24229971

5644782.387699966550316.8233392625069401.94743584320885.61959775543394.380233954029255.16554544465.144853185959308.725643792609402.712616933677671.3176674

5644782.387699966550316.8233392625069401.94743584320885.61959775543394.380233954029255.16554544465.144853185959308.725643792609402.712616933677671.3176674

5644782.387699966550316.8233392625069401.94743584320885.61959775543394.380233954029255.16554544465.144853185959308.725643792609402.712616933677671.3176674

4555055.172596871742941.741279912673546.564113361617590.92127346762480.638773641198997.38151848310.246483453071604.758407033189849.972768042794848.990054341367156.64038591

4555055.172596871742941.741279912673546.564113361617590.92127346762480.638773641198997.38151848310.246483453071604.758407033189849.972768042794848.990054341367156.64038591

826516.8646170313.632416742279905.323489957299669.602537959500846.936404713.6208768381215160.38669312319460.131278646639530.640069244

232533.032602215

3728538.307996871572628.108863172393641.24062341317921.318735446762480.63877364698150.44551443596.625606613071604.758407031974689.586074932242855.82617348727626.000316662

3912555.063393892193483.860922031834180.682021261718501.927457142096302.043761092793230.98191250021.636024471786747.956879011655665.24811115532777.2912155784689823.27484511

3912555.063393892193483.860922031834180.682021261718501.927457142096302.043761092793230.98191250021.636024471786747.956879011655665.24811115532777.2912155784689823.27484511

3912555.063393892193483.860922031834180.682021261718501.927457142096302.043761092793230.98191250021.636024471786747.956879011655665.24811115532777.2912155784689823.27484511

29816183.677605411632892.765811422999930.561298117652746.928687213509696.483222920446115.987110871154.945085530990537.717088725781961.481230339154718.170811639843446.9845259

29816183.677605411632892.765811422999930.561298117652746.928687213509696.483222920446115.987110871154.945085530990537.717088725781961.481230339154718.170811639843446.9845259

29816183.677605411632892.765811422999930.561298117652746.928687213509696.483222920446115.987110871154.945085530990537.717088725781961.481230339154718.170811639843446.9845259

40956095.593455246538724.517291630739420.673782311477044.880656113501812.720974317520104.722512749312.799442860153532.665633515091162.48219796465604.651815026956569.06979048

13117680.162986711636993.07460139031274.612248175804244.3329352710389262.69117366293068.40379480007.375546773193721.0600199611335501.41081234291857.918447825839339.11518465

13117680.162986711636993.07460139031274.612248175804244.3329352710389262.69117366293068.40379480007.375546773193721.0600199611335501.41081234291857.918447825839339.11518465

1323184.5582352210905848.05866542170354.300923641672905.13009149813755.498732929567704.8088797215.70045389830313.4123553121801313.045298641354612.8089321417730.856405823

1323184.5582352210905848.05866542170354.300923641672905.13009149813755.498732929567704.8088797215.70045389830313.4123553121801313.045298641354612.8089321417730.856405823

26515230.872233223995883.384024919537791.76061053999895.417629312298794.531067810659331.512472089.7234421456129498.19325821954348.02608691819133.924435095699499.0982

26515230.872233223995883.384024919537791.76061053999895.417629312298794.531067810659331.512472089.7234421456129498.19325821954348.02608691819133.924435095699499.0982

2336889.6773700513144189.07646035505149.230067317194144.131717751675839.925905171560918.53542268350.460048221708223.136432671623679.628361884310163.199455173247906.10162139

2226552.9114685812240251.77580714585475.6796846820.428776341570337.83809027703768.52561732723.61045471544047.405534791144969.893687343553643.982262813247906.10162139

2226552.9114685812240251.77580714585475.6796846820.428776341570337.83809027703768.52561732723.61045471544047.405534791144969.893687343553643.982262813247906.10162139

110336.765901466903937.300653132919673.551067316347323.702941408105502.0878149857150.0098535626.849593522164175.730897886478709.734674538756519.217192366

110336.765901466903937.300653132919673.551067316347323.702941408105502.0878149857150.0098535626.849593522164175.730897886478709.734674538756519.217192366

6481642.999063031050279.2227810613437902.49302893870462.1956984568241733.6687411901230.442920379769.94028291046122.322924771057904.185573229512339.718966681317801.20091164

6481642.999063031050279.2227810613437902.49302893870462.1956984568241733.6687411901230.442920379769.94028291046122.322924771057904.185573229512339.718966681317801.20091164

6481642.999063031050279.2227810613437902.49302893870462.1956984568241733.6687411901230.442920379769.94028291046122.322924771057904.185573229512339.718966681317801.20091164

12559904.08874242922047.04342215375634.02880934658533.2578263268887350.99432111178580.7051121307380.45798411939940.424852291790410.9539628912861765.0443685969265.775575769

6099951.00419029811796.588913069919.76811713701895.4148171467756926.2997783246622.931720145638.2886457578353.0598364011.3570221578925302.76248248661008.348510252

6099951.00419029811796.588913069919.76811713701895.4148171467756926.2997783246622.931720145638.2886457578353.0598364011.3570221578925302.76248248661008.348510252

6459953.084552162110250.4545222305714.26069217956637.8430091841130424.6945428931957.773411161742.169338341361587.365052291426399.596940743936462.28188606308257.427065517

6459953.084552162110250.4545222305714.26069217956637.8430091841130424.6945428931957.773411161742.169338341361587.365052291426399.596940743936462.28188606308257.427065517

158218.4671804983417396.728645692588201.539783132098053.146979492771045.415983228222457.06342412285.01159739653004.1062315481287699.98678247189761.5377961512325407.91270116

158218.4671804983417396.728645692588201.539783132098053.146979492771045.415983228222457.06342412285.01159739653004.1062315481287699.98678247189761.5377961512325407.91270116

158218.4671804983417396.728645692588201.539783132098053.146979492771045.415983228222457.06342412285.01159739653004.1062315481287699.98678247189761.5377961512325407.91270116

107107386.048496144136564.918721127343876.120342111981599.532771140866387.226587113305218.11604119495951.679918287267932.779186124235312.893789395822360.345515119438719.626849

3527399.021733746942757.323445125714468.253356142207053.47749331781104.440232376590132.34081124163.244309953787359.183652197809781.12738946543885.332362148698888.820961081

3527399.021733746942757.323445125714468.253356142207053.47749331781104.440232376590132.34081124163.244309953787359.183652197809781.12738946543885.332362148698888.820961081

3527399.021733746942757.323445125714468.253356142207053.47749331781104.440232376590132.34081124163.244309953787359.183652197809781.12738946543885.332362148698888.820961081

3527399.021733746942757.323445125714468.253356142207053.47749331781104.440232376590132.34081124163.244309953787359.183652197809781.12738946543885.332362148698888.820961081

4390587.5526603211637947.23067919165512.437775477348160.480601796325548.823239597077003.0596494167.223979664864951.828732385255429.8591649513190582.238690222755999.8557839

3221121.3454168370.109813684173599.3481551115.703607032408530.8171930883.222362961324269.668197612104609.6768552510852355.5719982818496.909986048

3221121.3454168370.109813684173599.3481551115.703607032408530.8171930883.222362961324269.668197612104609.6768552510852355.5719982818496.909986048

3221121.3454168370.109813684173599.3481551115.703607032408530.8171930883.222362961324269.668197612104609.6768552510852355.5719982818496.909986048

3570107.016854882938312.769474951759264.11259027459983.006423533257899.310950312904169.2272739049.36151922677691.304326688908319.494170488119005.488366899889621.40143103

3570107.016854882938312.769474951759264.11259027459983.006423533257899.310950312904169.2272739049.36151922677691.304326688908319.494170488119005.488366899889621.40143103

3570107.016854882938312.769474951759264.11259027459983.006423533257899.310950312904169.2272739049.36151922677691.304326688908319.494170488119005.488366899889621.40143103

820480.5358054365478513.116204153237878.215371512714578.126178261516533.808682251764303.0151824234.640097482862990.856208092242500.688139222219221.1783250221047881.5443668

820480.5358054365478513.116204153237878.215371512714578.126178261516533.808682251764303.0151824234.640097482862990.856208092242500.688139222219221.1783250221047881.5443668

820480.5358054365478513.116204153237878.215371512714578.126178261516533.808682251764303.0151824234.640097482862990.856208092242500.688139222219221.1783250221047881.5443668

52269337.01635442880142.832004938440371.677232534716398.708179633592750.078728327777921.0018439905443.725411228669256.429252245480998.3918531121133114.52004535004719.575857

40451783.991232240506295.333303330756961.514856530706847.065510630306917.658566824364591.2711434349263.541038623594701.120162243773104.5640953117886801.81161931949756.4205233

136692.4608181470.889478327.6566289886

136692.4608181470.889478327.6566289886

28223155.757288313107379.714306214418226.160749912151771.227507417809736.734002815793414.4567624105782.002580515632910.406521718317971.095022913182689.604356114264284.6156756

6095057.259201116041761.99245576225709.825550253717902.5952256310741037.21796737270824.2528617054194.4046856583704.350344269167053.807775283745969.796361167446101.4597025

11717940.54889166096102.912711497607764.225604097875042.452239095727171.309047188522590.20395564356.106383488554365.259362338700395.378227219138033.505775745971515.33432022

10410157.9491956969514.809138966532871.444472887558826.1800427341341528.206988241487231.49151202494840.796815141450521.909020364298686.302219155846667.821652901

51880.6651227063

2397171.450492943157117.138125556737371.387524317657808.411833335723973.13275973133913.42744058364.700628232562904.0055996818964234.91968745338520.017443254820343.98008899

155554.478521214245152.215549798233476.310278753250173.450090439188300.1232212141.085749499150052.40240416155328.5724368555121172.223362876

332028.47898863711919598.321135220979.430267868

223630.654

893859.408709309858523.1104977211110134.58020745885139.7179918191050939.0448954827777.16051037956.66156711619428.3874839872241820.071389447.199361498690495.447124943

1347757.563262421528616.648255685235698.942089335796218.964763533994873.504196021627804.92992435053.245211621943475.618115694310849.817799814549246.538054043654605.35680117

524825.163822347391537.865227535410944.939810593427987.13357784266400.5598373213.7081341914.307348365123518.277322993354070.9528

9767609.9035909524241798.48087169339247.1513391310897267.42616986773207.791804325163487.05086185116.83782995398886.708040846490898.5493850799287264.533190312865127.8247587

446674.90510172519848016.2984371810200.8981456386590851.2000702466418.4169120822409684.6838814910.4040968411952631.979234061215207.9357819332645221.71356528107541.3877313

9320934.998489234393782.18243458529046.253193494306416.22609966306789.374892242753802.3675370206.433733063446254.728806785275690.6136031466642042.81962524757586.43702736

63846.87986125424.35444308692305.44678

63846.87986125424.35444308692305.44678

11817553.02512192373847.498701647683410.1623764009551.642669043285832.420161533413329.73075556180.184372615074555.309089961707893.827757783246312.708426373054963.15533372

11817553.02512192373847.498701647683410.1623764009551.642669043285832.420161533413329.73075556180.184372615074555.309089961707893.827757783246312.708426373054963.15533372

4854232.478428512275255.197458675204458.111024834009551.642669042798338.723939893413329.73075190939.315507185074555.309089961469557.800602063067810.42541243054963.15533372

6963320.5466933598592.30124296222478952.05135117487493.696221644365240.868865433238336.02715572178502.283013977

29185380.428765163883700.398987355215920.001240647955647.389424588440442.022087556945311.272359921902.9976212217608890.85987745677327.4895534202843183.5884448213941.3286036

950935.393909311415303.610087362205055.574114532510509.1913966916770821.19985952657084.29224801861.928098791112412.858190833633250.166899584771886.144369494566319.53935529

950935.393909311415303.610087362205055.574114532510509.1913966916770821.19985952657084.29224801861.928098791112412.858190833633250.166899584771886.144369494566319.53935529

950935.393909311415303.610087362205055.574114532510509.1913966916770821.19985952657084.29224801861.928098791112412.858190833633250.166899584771886.144369494566319.53935529

28074221.266156662119599.737674652370719.080707244896921.498427870232444.353818653968207.684953697384.575202216496478.00168641432847.8863326192440947.36415842685406.8417237

28074221.266156662119599.737674652370719.080707244896921.498427870232444.353818653968207.684953697384.575202216496478.00168641432847.8863326192440947.36415842685406.8417237

28074221.266156662119599.737674652370719.080707244896921.498427870232444.353818653968207.684953697384.575202216496478.00168641432847.8863326192440947.36415842685406.8417237

160223.768699132348797.051225313640145.34641885548216.69961437176.46840948320019.29521422656.49432047611229.4363212565630350.07991275962214.947524575

160223.768699132348797.051225313640145.34641885548216.69961437176.46840948320019.29521422656.49432047611229.4363212565630350.07991275962214.947524575

160223.768699132348797.051225313640145.34641885548216.69961437176.46840948320019.29521422656.49432047611229.4363212565630350.07991275962214.947524575

214967.2817182269.4211300568357692.78782651

214967.2817182269.4211300568357692.78782651

214967.2817182269.4211300568357692.78782651

214967.2817182269.4211300568357692.78782651

17734682.028982918792017.133604618807603.750737219754339.477071611726541.862299620699883.160412050274.488595732155205.056541720011776.025828449753901.878150712765170.0456431

12591195.864798515387228.909556913912956.603639516620863.33407889908133.2376737714318856.597910337822.065524218204951.799371514567271.76010356898117.6872827310442475.2051685

1879194.54753882817296.2544744683059446.16241789680203.6078091914519878.159521862114320.9891708486.02420506244227.585137076532032.4832061561177634.035326961220157.44007139

1879194.54753882817296.2544744683059446.16241789680203.6078091914519878.159521862114320.9891708486.02420506244227.585137076532032.4832061561177634.035326961220157.44007139

6742610.432267277480889.043553266138766.2797519211194396.62304823668884.917985958767978.8174748678.712349715085995.4410611716.63267993078145.488069497507171.761

6742610.432267277480889.043553266138766.2797519211194396.62304823668884.917985958767978.8174748678.712349715085995.4410611716.63267993078145.488069497507171.761

3969390.88499247089043.611529174714744.16146974746263.10322141719370.160165963436556.79193880657.328969412874728.774234473423522.644217442642338.163886291715146.00409711

3969390.88499247089043.611529174714744.16146974746263.10322141719370.160165963436556.79193880657.328969412874728.774234473423522.644217442642338.163886291715146.00409711

2594244.3621190462.337886521797954.28338018806656.59292268419.78161007514977.3664558484832.4568849055

2594244.3621190462.337886521797954.28338018806656.59292268419.78161007514977.3664558484832.4568849055

2594244.3621190462.337886521797954.28338018806656.59292268419.78161007514977.3664558484832.4568849055

2549241.802184372214325.88616123096692.863717533133476.142992851818408.624625875574369.96961712452.4230715211681833.47556014929526.8992690942770951.73398312322694.84047463

185215.933143489308198.099177975457368.8413539653856449.578193899.491438951301382.90425374493323.709050181

185215.933143489308198.099177975457368.8413539653856449.578193899.491438951301382.90425374493323.709050181

2364025.869040882214325.88616122788494.764539552676107.301638881818408.624625871717920.39161518552.9316325711681833.47556014628143.9950153542277628.02493292322694.84047463

2364025.869040882214325.88616122788494.764539552676107.301638881818408.624625871717920.39161518552.9316325711681833.47556014628143.9950153542277628.02493292322694.84047463

29178233.464835219225017.602253215631570.966709319728545.30758910762129.13189959919865.953718773057.882263113392626.339732526811883.02754948163522.978112929391093.28401216

29178233.464835219225017.602253215631570.966709319728545.30758910762129.13189959919865.953718773057.882263113392626.339732526811883.02754948163522.978112929391093.28401216

22116557.852439411717936.34909477355488.1276989215251636.04385954578354.496680225077768.34434377791.130520526895047.7162655917356676.24854154604387.202401116786868.2579697

22116557.852439411717936.34909477355488.1276989215251636.04385954578354.496680225077768.34434377791.130520526895047.7162655917356676.24854154604387.202401116786868.2579697

22116557.852439411717936.34909477355488.1276989215251636.04385954578354.496680225077768.34434377791.130520526895047.7162655917356676.24854154604387.202401116786868.2579697

7061675.612395837507081.25315858276082.839010374476909.263729456183774.635219284842097.609414395266.75174266497578.623466959455206.779007943559135.775711812604225.02604245

7061675.612395837507081.25315858276082.839010374476909.263729456183774.635219284842097.609414395266.75174266497578.623466959455206.779007943559135.775711812604225.02604245

7061675.612395837507081.25315858276082.839010374476909.263729456183774.635219284842097.609414395266.75174266497578.623466959455206.779007943559135.775711812604225.02604245

7427010.507303152228486.721952485099052.151609693304524.6648470910047927.00130122568128.40095691273.936594637415521.009135399861859.507415975040692.183695416266024.7198974

3565872.9633421257.7491303722408533.65189266596832.499660984922284.6933514191212646.55032267782.907529966083754.182537056247607.444559311062449.422923515351470.08245332

3565872.9633421257.7491303722408533.65189266596832.499660984922284.6933514191212646.55032267782.907529966083754.182537056247607.444559311062449.422923515351470.08245332

3565872.9633421257.7491303722408533.65189266596832.499660984922284.6933514191212646.55032267782.907529966083754.182537056247607.444559311062449.422923515351470.08245332

3565872.9633421257.7491303722408533.65189266596832.499660984922284.6933514191212646.55032267782.907529966083754.182537056247607.444559311062449.422923515351470.08245332

920836.791791737367691.811022807771582.717370647977850.3647491281520189.285280551024448.9631921269.91995518340469.4889421551414805.101304351159198.178367710262275.1989256

920836.791791737367691.811022807771582.717370647977850.3647491281520189.285280551024448.9631921269.91995518340469.4889421551414805.101304351159198.178367710262275.1989256

920836.791791737367691.811022807771582.717370647977850.3647491281520189.285280551024448.9631921269.91995518340469.4889421551414805.101304351159198.178367710262275.1989256

920836.791791737367691.811022807771582.717370647977850.3647491281520189.285280551024448.9631921269.91995518340469.4889421551414805.101304351159198.178367710262275.1989256

2940300.752211421439537.16179931918935.782346381729841.800436977605453.0226692331032.88761502221.10910949991297.3376561892199446.961552312819044.58240419652279.438518464

2940300.752211421439537.16179931918935.782346381729841.800436977605453.0226692331032.88761502221.10910949991297.3376561892199446.961552312819044.58240419652279.438518464

265513.782440615890548.06481040150.16809128913089.3677989871451390.0474261218170.8481862895.58519797283953.171192987771041.578515763821096.623204191

265513.782440615890548.06481040150.16809128913089.3677989871451390.0474261218170.8481862895.58519797283953.171192987771041.578515763821096.623204191

2674786.9697708548989.096999303878785.614255095816752.4326379886154062.9752431112862.0395639325.523911523707344.1664632021428405.383036551997947.9592652279.438518464

260004.386770802140653.5552

2414782.583548989.096999303878785.614255095816752.4326379886154062.9752431112862.0395639325.523911523707344.1664632021428405.383036551857294.404652279.438518464

85773.5429187597999158.144254909513340.477335615638896.931976181207192.675139074928102.1999100257.7190166171009541.14816277

85773.5429187597999158.144254909513340.477335615638896.931976181207192.675139074928102.1999100257.7190166171009541.14816277

85773.5429187597999158.144254909513340.477335615638896.931976181207192.675139074928102.1999100257.7190166171009541.14816277

85773.5429187597999158.144254909513340.477335615638896.931976181207192.675139074928102.1999100257.7190166171009541.14816277

85773.5429187597999158.144254909513340.477335615638896.931976181207192.675139074928102.1999100257.7190166171009541.14816277

85773.5429187597999158.144254909513340.477335615638896.931976181207192.675139074928102.1999100257.7190166171009541.14816277

45739653.498912214794963.233167324236464.219936313927046.321892115172848.893623310486787.8041619814664.427288427549878.874117844289.270097832534314.639753116545867.5299886

45739653.498912214794963.233167324236464.219936313927046.321892115172848.893623310486787.8041619814664.427288427549878.874117844289.270097832534314.639753116545867.5299886

217966.257638971445640.526742992448114.21427560186743.5418524459552686.86315793265793.5626

217966.257638971445640.526742992448114.21427560186743.5418524459552686.86315793265793.5626

217966.257638971445640.526742992448114.21427560186743.5418524459552686.86315793265793.5626

217966.257638971445640.526742992448114.21427560186743.5418524459552686.86315793265793.5626

2706707.161791469.9764804762227616.03695713805051.329520631154078.555406831902353.823766563503760.31692675982243.6382761943.2747892741357704.63919229

2706707.161791469.9764804762227616.03695713805051.329520631154078.555406831902353.823766563503760.31692675982243.6382761943.2747892741357704.63919229

2706707.161791469.9764804762227616.03695713805051.329520631154078.555406831902353.823766563503760.31692675982243.6382761943.2747892741357704.63919229

2706707.161791469.9764804762227616.03695713805051.329520631154078.555406831902353.823766563503760.31692675982243.6382761943.2747892741357704.63919229

370262.102041913356485.026451322549709.334638153533371.351065522090474.617430372044001.603212115809.372336274453618.877557621089606.240884682225431.79937763

370262.102041913356485.026451322549709.334638153533371.351065522090474.617430372044001.603212115809.372336274453618.877557621089606.240884682225431.79937763

370262.102041913356485.026451322549709.334638153533371.351065522090474.617430372044001.603212115809.372336274453618.877557621089606.240884682225431.79937763

1724839.263365071314620.531705051603530.55095409277286.00975112950030.71521428674.958526031905038.81840140151863.87838

370262.102041911631645.763086251235088.80293311929840.800111431813188.607679251093970.8881687134.413810243548580.059156221037742.362504682225431.79937763

16605822.63967888009716.7170130916494838.93186647435888.091104669997855.670911355257001.3335514087147.183821623038672.462084110622406.236736930046330.819890410676541.4470898

16605822.63967888009716.7170130916494838.93186647435888.091104669997855.670911355257001.3335514087147.183821623038672.462084110622406.236736930046330.819890410676541.4470898

16605822.63967888009716.7170130916494838.93186647435888.091104669997855.670911355257001.3335514087147.183821623038672.462084110622406.236736930046330.819890410676541.4470898

16605822.63967888009716.7170130916494838.93186647435888.091104669997855.670911355257001.3335514087147.183821623038672.462084110622406.236736930046330.819890410676541.4470898

25838895.33855252191650.986479462516185.702198982065992.00834881377753.186716852919991.30481709354.04736391007446.09508911786020.51760325636434.3041887192286189.64432892

25838895.33855252191650.986479462516185.702198982065992.00834881377753.186716852919991.30481709354.04736391007446.09508911786020.51760325636434.3041887192286189.64432892

25838895.33855252191650.986479462516185.702198982065992.00834881377753.186716852919991.30481709354.04736391007446.09508911786020.51760325636434.3041887192286189.64432892

25838895.33855252191650.986479462516185.702198982065992.00834881377753.186716852919991.30481709354.04736391007446.09508911786020.51760325636434.3041887192286189.64432892

994684.0076937635928133.490172734644222.654184018961461.565302162740847.329011346962948.64554568277.8023826913299117.91550666907946.985954437772748.2145951313521386.8206487

865285.0498074595384078.924966093300348.011118374817456.72274352551477.791868776503490.82493393632.5433321813299117.91550664012194.629520084493069.838380683818213.12470294

659357.4523457963248942.210112513133640.456705464277271.258709641010756.437255124400918.81041917012.6330174812306322.25122064012194.629520084431214.335920682202196.27294685

659357.4523457963248942.210112513066405.780485464277271.258709641010756.437255123733659.45971917012.6330174810967882.95480763867836.366805994410239.002049992202196.27294685

246388.441677161126752.34553097833441.1438161061

246388.441677161126752.34553097833441.1438161061

659357.4523457963002553.768435353066405.780485464150518.91317867977315.2934390173733659.45971917012.6330174810967882.95480763867836.366805994410239.002049992202196.27294685

659357.4523457962707230.558470112250079.015923862613476.74210261977315.2934390173171680.34131296179.427810191493787.363971123554777.279410364019442.252949991609421.2332626

295323.209965243816326.7645616081537042.17107606561979.1184620833.2052072979474095.59083644313059.087395626390796.7491592775.03968425

67234.67622144358.26271408620975.3338706844

67234.67622144358.26271408620975.3338706844

67234.67622144358.26271408620975.3338706844

667259.35071338439.296413

667259.35071338439.296413

667259.35071338439.296413

205927.5974616632135136.71485358166707.554412901540185.4640338541540721.354613642102572.01451476619.91031469992795.66428607461855.502461616016.85175609

205927.5974616632135136.71485358166707.554412901540185.4640338541540721.354613642102572.01451476619.91031469992795.66428607461855.502461616016.85175609

1781796.12761343540185.4640338541540721.354613641748315.1191476619.91031469992795.6642860741616016.85175609

1781796.12761343540185.4640338541540721.354613641748315.1191476619.91031469992795.6642860741616016.85175609

205927.597461663353340.587240157166707.554412901354256.895561855.50246

205927.597461663353340.587240157166707.554412901354256.895561855.50246

143846.325328909155385.339959135136418.451876699189369.537142572129263.5631184165.882986297

143846.325328909155385.339959135136418.451876699189369.537142572129263.5631184165.882986297

143846.325328909155385.339959135136418.451876699189369.537142572129263.5631184165.882986297

143846.325328909155385.339959135136418.451876699189369.537142572129263.5631184165.882986297

143846.325328909155385.339959135136418.451876699189369.537142572129263.5631184165.882986297

129398.957886304400208.2398777291188489.303106514007586.39068197330194.2575990479.3760642152895752.356434353279678.376214459703173.69594573

129398.957886304400208.239877729709088.1489324821838301.05224032368073.1187214962895752.356434353279678.37621445

129398.957886304400208.239877729709088.1489324821838301.05224032368073.1187214962895752.356434353279678.37621445

129398.957886304400208.239877729709088.1489324821838301.05224032368073.1187214962895752.356434353279678.37621445

129398.957886304156499.50962714594744.0276740941630416.6716663368073.118721496440672.9913323722983834.9678483

243708.730250589114344.121258388207884.3805740182455079.36510198295843.408366145

151454.973333508798019.299079346176458.6046113576.8995131074655084.498

151454.973333508798019.299079346176458.6046113576.8995131074655084.498

151454.973333508798019.299079346176458.6046113576.8995131074655084.498

151454.973333508798019.299079346176458.6046113576.8995131074655084.498

327946.180840521371266.0393623153735.6529508829.3578296125048089.19794573

327946.180840521371266.0393623153735.6529508829.3578296125048089.19794573

327946.180840521371266.0393623153735.6529508829.3578296125048089.19794573

327946.180840521371266.0393623153735.6529508829.3578296125048089.19794573

23081713.370267426547778.601805716710260.032364828372325.044239629887804.54425513693510.2272323991342.19416975140681.203012619659171.517411616312514.690388510972766.7689301

23081713.370267426547778.601805716710260.032364828372325.044239629887804.54425513693510.2272323991342.19416975140681.203012619659171.517411616312514.690388510972766.7689301

23081713.370267426547778.601805716710260.032364828372325.044239629887804.54425513693510.2272323991342.19416975140681.203012619659171.517411616312514.690388510972766.7689301

23081713.370267426547778.601805716710260.032364828372325.044239629887804.54425513693510.2272323991342.19416975140681.203012619659171.517411616312514.690388510972766.7689301

23081713.370267426547778.601805716710260.032364828372325.044239629887804.54425513693510.2272323991342.19416975140681.203012619659171.517411616312514.690388510972766.7689301

23081713.370267426547778.601805716710260.032364828372325.044239629887804.54425513693510.2272323991342.19416975140681.203012619659171.517411616312514.690388510972766.7689301

85985.934865889471366.9928458538136.7109887541047059.12104795647712.90665982502513.38368707415.151645852795920.7688615096018752.48446657580334.466611356

85985.934865889471366.9928458538136.7109887541047059.12104795647712.90665982502513.38368707415.151645852795920.7688615096018752.48446657580334.466611356

85985.934865889471366.9928458538136.7109887541047059.12104795647712.90665982502513.38368707415.151645852795920.7688615096018752.48446657580334.466611356

85985.934865889471366.9928458538136.7109887541047059.12104795647712.90665982502513.38368707415.151645852795920.7688615096018752.48446657580334.466611356

85985.934865889471366.9928458538136.7109887541047059.12104795647712.90665982502513.38368707415.151645852795920.7688615096018752.48446657580334.466611356

85985.934865889471366.9928458538136.7109887541047059.12104795647712.90665982502513.38368707415.151645852795920.7688615096018752.48446657580334.466611356

64659379.208298125421458.91013741021168.207309442458524.3710573188789087.33467634374016.78565216271887.63453320141439.852323947564212.139882935873282.843837528881684.2890964

13498980.569725810931614.90808168569269.2945954410541670.946070915693988.844418312249629.1346311667853.3951247792341.4026407620933721.722064710379910.67013227746999.48500557

2465475.83220027930456.72534409817018.035380109282510.070452847633393.8339473113.755221205221859.6629543506.722538391771964.727602991501663.47247351

2465475.83220027930456.72534409817018.035380109282510.070452847633393.8339473113.755221205221859.6629543506.722538391771964.727602991501663.47247351

2465475.83220027930456.72534409817018.035380109282510.070452847633393.8339473113.755221205221859.6629543506.722538391771964.727602991501663.47247351

2465475.83220027930456.72534409817018.035380109282510.070452847633393.8339473113.755221205221859.6629543506.722538391771964.727602991501663.47247351

9700184.998262766365690.819881514975254.678510617932651.3309321814111405.93239349169191.339739835579.600703414803255.7688071219685794.59553738007432.797653415914993.49910149

9700184.998262766365690.819881514975254.678510617932651.3309321814111405.93239349169191.339739835579.600703414803255.7688071219685794.59553738007432.797653415914993.49910149

9700184.998262766365690.819881514975254.678510617932651.3309321814111405.93239349169191.339739835579.600703414803255.7688071219685794.59553738007432.797653415914993.49910149

9700184.998262766365690.819881514975254.678510617932651.3309321814111405.93239349169191.339739835579.600703414803255.7688071219685794.59553738007432.797653415914993.49910149

731785.7246158931909280.511893582628856.325295142259500.488908271470745.46849781367375.491204758.252066262767225.97093364499920.657896499291206.0390277480760.5757025382

731785.7246158931909280.511893582628856.325295142259500.488908271470745.46849781367375.491204758.252066262767225.97093364499920.657896499291206.0390277480760.5757025382

731785.7246158931909280.511893582628856.325295142259500.488908271470745.46849781367375.491204758.252066262767225.97093364499920.657896499291206.0390277480760.5757025382

731785.7246158931909280.511893582628856.325295142259500.488908271470745.46849781367375.491204758.252066262767225.97093364499920.657896499291206.0390277480760.5757025382

601534.0146468311726186.85096247148140.25540958367009.0557776498111837.4435271531079668.471154401.787133085204499.746092487309307.105848056249581.93772803

601534.0146468311726186.85096247148140.25540958367009.0557776498111837.4435271531079668.471154401.787133085204499.746092487309307.105848056249581.93772803

601534.0146468311726186.85096247148140.25540958367009.0557776498111837.4435271531079668.471154401.787133085204499.746092487309307.105848056249581.93772803

601534.0146468311726186.85096247148140.25540958367009.0557776498111837.4435271531079668.471154401.787133085204499.746092487309307.105848056249581.93772803

4692577.334152931218009.621848062700921.264397611725404.654589371147162.269369312610561.9281372771.118057681339975.018828343690055.36074103531384.0119453541519470.98586185

4692577.334152931218009.621848062700921.264397611725404.654589371147162.269369312610561.9281372771.118057681339975.018828343690055.36074103531384.0119453541519470.98586185

4692577.334152931218009.621848062700921.264397611725404.654589371147162.269369312610561.9281372771.118057681339975.018828343690055.36074103531384.0119453541519470.98586185

4692577.334152931218009.621848062700921.264397611725404.654589371147162.269369312610561.9281372771.118057681339975.018828343690055.36074103531384.0119453541519470.98586185

4692577.334152931218009.621848062700921.264397611725404.654589371147162.269369312610561.9281372771.118057681339975.018828343690055.36074103531384.0119453541519470.98586185

2824069.029787752917980.174248264629914.792442673096139.944749112940244.389162723917873.581243242766.849094164837491.571296426141986.64093963299384.355033044028853.4559191

2109939.729682382396975.15835742630012.136605882382848.705619872940244.389162723716712.00662858663.325522274739490.718247395627747.425022551526643.458970484028853.4559191

2109939.729682382396975.15835742630012.136605882382848.705619872940244.389162723716712.00662858663.325522274739490.718247395627747.425022551526643.458970484028853.4559191

2109939.729682382396975.15835742630012.136605882382848.705619872940244.389162723716712.00662858663.325522274739490.718247395627747.425022551526643.458970484028853.4559191

2109939.729682382396975.15835742630012.136605882382848.705619872940244.389162723716712.00662858663.325522274739490.718247395627747.425022551526643.458970484028853.4559191

714129.300105373521005.0158908551999902.6558368713291.239129243201161.57464384103.52357189198000.8530490375514239.2159170431772740.89606256

714129.300105373521005.0158908551999902.6558368713291.239129243201161.57464384103.52357189198000.8530490375514239.2159170431772740.89606256

714129.300105373521005.0158908551999902.6558368713291.239129243201161.57464384103.52357189198000.8530490375514239.2159170431772740.89606256

714129.300105373521005.0158908551999902.6558368713291.239129243201161.57464384103.52357189198000.8530490375514239.2159170431772740.89606256

43643752.274631610353854.205959125121062.855873627095308.8256479169007691.83172615595952.14178199988496.2722576171631.8595584216798448.416137521662603.806726915586360.3623099

32809043.94154422142457.455453861592329.665224992805428.9081972827313673.46728081351524.196226536374.97280013233155.634607811109328.429444513662053.2225845911096802.8264568

32809043.94154422142457.455453861592329.665224992805428.9081972827313673.46728081351524.196226536374.97280013233155.634607811109328.429444513662053.2225845911096802.8264568

32809043.94154422142457.455453861592329.665224992805428.9081972827313673.46728081351524.196226536374.97280013233155.634607811109328.429444513662053.2225845911096802.8264568

32809043.94154422142457.455453861592329.665224992805428.9081972827313673.46728081351524.196226536374.97280013233155.634607811109328.429444513662053.2225845911096802.8264568

3099992.061501316189464.218837996618738.220708294755237.425801571333093.43992889472496.69939148254011.666971511047.0331408371672546.947422711212498.07823555943217.659130454

3099992.061501316189464.218837996618738.220708294755237.425801571333093.43992889472496.69939148254011.666971511047.0331408371672546.947422711212498.07823555943217.659130454

3099992.061501316189464.218837996618738.220708294755237.425801571333093.43992889472496.69939148254011.666971511047.0331408371672546.947422711212498.07823555943217.659130454

67445846.4428513145079665.632862653663.764598546

2094873.502766144937095.476459745787328.783025932064466.338648483294919.08877988620078.1282016522.104436691013779.96036121235998.574843072943217.659130454

1005118.558735181252368.74237825831409.4376823662690771.08715302592327.908297769852418.571391157823.92967169511047.033140837658766.987061504322835.738793933

7573622.717486161722970.6172136716079887.997884118622552.913849170070373.533142998672.4561924529629.03071531778886.1393331113515303.740765613984931.46363142308420.92224848

7573622.717486161722970.6172136716079887.997884118622552.913849170070373.533142998672.4561924529629.03071531778886.1393331113515303.740765613984931.46363142308420.92224848

7573622.717486161722970.6172136716079887.997884118622552.913849170070373.533142998672.4561924529629.03071531778886.1393331113515303.740765613984931.46363142308420.92224848

7573622.717486161722970.6172136716079887.997884118622552.913849170070373.533142998672.4561924529629.03071531778886.1393331113515303.740765613984931.46363142308420.92224848

161093.5541298961.914453558830106.972056278912089.5778290551.3913760931773258.79668480.601770689648543.052476664501269.2985046852803121.042275381237918.95447415

161093.5541298961.914453558830106.972056278912089.5778290551.3913760931773258.79668480.601770689648543.052476664501269.2985046852803121.042275381237918.95447415

161093.5541298961.914453558830106.972056278912089.5778290551.3913760931773258.79668480.601770689648543.052476664501269.2985046852803121.042275381237918.95447415

161093.5541298961.914453558830106.972056278912089.5778290551.3913760931773258.79668480.601770689648543.052476664501269.2985046852803121.042275381237918.95447415

176700870.722246188860405.137656268220899.984226181779742.483894176185477.01532182421910.91031190081587.93081172834320.007652202014575.71165157266303.150208199309166.575603

6174547.840416739213244.5943108725043948.205500311040097.61879429201388.4574648622212878.727337965935.745896875629869.914302019612661.08709968202752.5936808911141200.9279988

6174547.840416739213244.5943108725043948.205500311040097.61879429201388.4574648622212878.727337965935.745896875629869.914302019612661.08709968202752.5936808911141200.9279988

5359787.483641218060660.0836399923018666.09858539257611.63909487759455.215184817659348.39056954426.288622521481700.22356084484808.59853227448046.18013623165038.16303165

5359787.483641218060660.0836399923018666.09858539257611.63909487759455.215184817659348.39056954426.288622521481700.22356084484808.59853227448046.18013623165038.16303165

2799037.237765115614908.1456845820912451.21497495529482.798882314811364.6559494412007619.09584340452.730499231405859.81913082334114.469106446620229.187747581568744.62764681

1521902.10473079992720.6805821151141290.345316862046778.034556431030779.710280781923612.4021603447.81392681573244.3200137459923.0897927434.476741163

647842.858245308132762.714104834633642.3802028553520953.364

391005.2829452348.854719161832161.824188716737041.90121867347557.131017284207163.5287265239.61940703675840.40443577449.80941206367893.902688618668859.058643678

1000682.40265414944308.904437387936111.337734442745286.12478946

814760.3567755211152584.510670882025282.106914971782485.979699391441933.242280064553530.336831011509.457274344148169.690741215127852.4885674754706.413544697976162.7649672

814760.3567755211152584.510670882025282.106914971782485.979699391441933.242280064553530.336831011509.457274344148169.690741215127852.4885674754706.413544697976162.7649672

126256.120047405348272.442317981446969.612525087483800.938871521565771.728392851766424.126855534118019.582223475342244.850726455148858.2655

688504.236728115804312.0683528951578312.494389881298685.04082787876161.513887214553530.33683245085.3304188114030150.108517744785607.63784095605848.148044697976162.7649672

170016904.479273177572055.101183242766407.6404169927439.1115164171177.403039158638808.41368178589308.780127159952561.13135187323060.781446148602967.624174187882774.386

1899293.876423472986711.023637742265216.89692461711417.7967224391853007.13719693266545.97381392974.90212916437214.023360511750839.92899735155132.127489689

1899293.876423472986711.023637742265216.89692461711417.7967224391853007.13719693266545.97381392974.90212916437214.023360511750839.92899735155132.127489689

1899293.876423472986711.023637742265216.89692461711417.7967224391853007.13719693266545.97381392974.90212916437214.023360511750839.92899735155132.127489689

1899293.876423472986711.023637742265216.89692461711417.7967224391853007.13719693266545.97381392974.90212916437214.023360511750839.92899735155132.127489689

122745432.818291133892125.817907200441939.598545134603895.860175137089391.396063127187183.15646152982645.010284116575275.215579154857856.095516117138007.433802126489656.092868

373243.4147189159.850197849319918.539242751164209.776761094270852.6693659831.680487466665262.209725035385297.5939570891101929.28292452

373243.4147189159.850197849319918.539242751164209.776761094270852.6693659831.680487466665262.209725035385297.5939570891101929.28292452

373243.4147189159.850197849319918.539242751164209.776761094270852.6693659831.680487466370316.658525035385297.5939570891101929.28292452

294945.5512

14506595.370175712868085.200541652286140.060521918357966.29983369138326.219894698233304.4702813459110.663684112525015.54500778493470.385700325122796.338976678777512.48039173

14506595.370175712868085.200541652286140.060521918357966.29983369138326.219894698233304.4702813459110.663684112525015.54500778493470.385700325122796.338976678777512.48039173

9225311.652413413558513.9136119947156269.6329112214096.13797063584117.979717473423416.813787805075.822140875309374.891134593799229.374044341643695.647243461688822.4369003

5281283.717762269309571.286929655129870.427611896143870.161863035554208.240177224809887.65655654034.841543277215640.653873154694241.011655993479100.691733217088690.04349143

1475350.098345392913471.07251826324.6913471699.28822572474596.387421866100281.4462406551.030599141274056.467115345219296.562305681968601.655685742497503.29378377

1475350.098345392913471.07251826324.6913471699.28822572474596.387421866100281.4462406551.030599141274056.467115345219296.562305681968601.655685742497503.29378377

1475350.098345392913471.07251826324.6913471699.28822572474596.387421866100281.4462406551.030599141274056.467115345219296.562305681968601.655685742497503.29378377

27758395.315930339004301.123312234438662.172685115392989.771211327528507.736882513832882.4515430870942.937799215245907.849928115189175.98627515063994.166132619331990.6658603

479913.404020962543704.15308411495128.443382128639833.270892147668361.6854882651020065.69503060.9452821251319834.46470282166668.337352691491182.505896749

479913.404020962543704.15308411495128.443382128639833.270892147668361.6854882651020065.69503060.9452821251319834.46470282166668.337352691491182.505896749

27278481.911909338460596.970228133943533.729302914753156.500319126860146.051394212812816.7615430367881.992517115245907.849928113869341.521572214897325.828779918840808.1599635

11023775.43444832335245.182759812182671.772660792980306.147994481509337.279424093193457.50751347070.392715973564189.083967121179698.278779061674623.20978053068797.80421369

818858.5508037113504911.380400111770168.62926816479771.0613380511852464.114902783547766.40893800248.18286926204717.61151156960.887345851215111.05407341559333.59467291

84315.51215154965.072978923

1068678.5739885608748.23181362438394.031957072914983.692574541758131.7439676116.42621054962.197267292437367.6996223676.0101513991521762.64536749332786.309738241

3796258.72412382910205.714966725421784.620556712063311.687788863695193.66879647771711.749385822740.201303743504659.068090432041003.407939382825511.690509741483617.5616856

1541822.034329281019935.540176751157861.789184851791883.54528177360397.080888851799384.30610043756.1203578644981.30314730145.0962873562278.202011273693910.932929881

210390.006635164366199.186869053414864.492992866249001.647418519424616.2672202632.326743678364555.652069581

8818698.5875805627715351.73324222972652.88567536108035.8723486111435620.51606352399764.096368299104.898003024687360.756926874537857.841069226649167.8628179211547396.8837443

15287957.228177316555896.47407533609499.07171119224408.839369731841900.962713314899820.806727332704.415441413433122.083824818461749.596686110078321.472370417602191.1144638

11375185.822389711769679.931110328183863.02790795611514.68356926376128.367410210329663.358421716000.18831267800098.594346799187510.333219122724475.140634066877746.16809284

11375185.822389711769679.931110328183863.02790795611514.68356926376128.367410210329663.358421716000.18831267800098.594346799187510.333219122724475.140634066877746.16809284

3912771.405787644786216.542964695425636.043803183612894.15580075465772.595303164570157.44835616704.227128825633023.489478029274239.263466957353846.3317363210724444.9463709

3912771.405787644786216.542964695425636.043803183612894.15580075465772.595303164570157.44835616704.227128825633023.489478029274239.263466957353846.3317363210724444.9463709

809779.907751417959503.3734656191467161.598511242064439.761130461509725.187258797739850.2392378362.700019462049.7769312081326682.71739902253779.69048541032.64268026

809779.907751417959503.3734656191467161.598511242064439.761130461509725.187258797739850.2392378362.700019462049.7769312081326682.71739902253779.69048541032.64268026

809779.907751417959503.3734656191467161.598511242064439.761130461509725.187258797739850.2392378362.700019462049.7769312081326682.71739902253779.69048541032.64268026

4869830.389418016618002.858366537626688.4426670333074123.5614797551985.416996797920569.30714994954.373468613386278.752116056188567.074360462641276.096379993419096.63062611

4869830.389418016618002.858366537626688.4426670333074123.5614797551985.416996797920569.30714994954.373468613386278.752116056188567.074360462641276.096379993419096.63062611

1344747.097155721835410.102668231372330.386188173259533.745295921105779.98302542662671.01221148993.2366155521462.1817532231079827.77607914422518.3258049611641916.37990516

3525083.292262294782592.755698296254358.0564788629814589.8161836446205.433971377257898.29493845961.136853122864816.570362835108739.298281312218757.770575031777180.25072095

3972955.352216482162803.133012971287506.724056491439194.247822731235191.36949031284697.72534811724.0185128655373851.792847692123322.22025039817615.965415271692281.234020903

3972955.352216482162803.133012971287506.724056491439194.247822731235191.36949031284697.72534811724.0185128655373851.792847692123322.22025039817615.965415271692281.234020903

3972955.352216482162803.133012971287506.724056491439194.247822731235191.36949031284697.72534811724.0185128655373851.792847692123322.22025039817615.965415271692281.234020903

1951006.514161971875740.52373481705350.441800512346597.502706541166657.862560427692826.48142476595.188619553082131.236496882642390.397839137616392.282263885559694.58484088

1951006.514161971875740.52373481705350.441800512346597.502706541166657.862560427692826.48142476595.188619553082131.236496882642390.397839137616392.282263885559694.58484088

1951006.514161971875740.52373481705350.441800512346597.502706541166657.862560427692826.48142476595.188619553082131.236496882642390.397839137616392.282263885559694.58484088

3054267.59722093013264.426670674792361.555132444070844.970943152720768.8419727210580523.66184307353.76449533938628.478137384123242.6430575217845083.89176175947232.80309448

3054267.59722093013264.426670674792361.555132444070844.970943152720768.8419727210580523.66184307353.76449533938628.478137384123242.6430575217845083.89176175947232.80309448

915536.515837651369275.284818284598027.14844507153975.3411763862.493619914530308.024524219576014.238731502304423.6016

2138731.081383253013264.426670674423086.270314163472817.822498082720768.8419727210526548.32073543491.270875383938628.478137383592934.618533317269069.65303025642809.20149448

279104.031539892190252.62714674827849.327355551449334.171832284261362.425336192268874.705430852714844.32143270789866.6400300742

279104.031539892190252.62714674827849.327355551449334.171832284261362.425336192268874.705430852714844.32143270789866.6400300742

279104.031539892190252.62714674827849.327355551449334.171832284261362.425336192268874.705430852714844.32143270789866.6400300742

3680595.340424934754688.364340263343879.874568043607820.567145017585403.589162476387871.32869620844.9770783714729436.579367718130214.854903321638436.11280424845125.79264737

3680595.340424934754688.364340263343879.874568043607820.567145017585403.589162476387871.32869620844.9770783714729436.579367718130214.854903321638436.11280424845125.79264737

3680595.340424934754688.364340263343879.874568043607820.567145017585403.589162476387871.32869620844.9770783714729436.579367718130214.854903321638436.11280424845125.79264737

27614601.815933222402687.702557630935498.622252422759965.376655521886008.336318823421402.2711624435474.82282828476304.316856350682215.944836625470763.123977834311659.7194421

1058675.562903382173.149610722871911.679897401180653.745115238665696.23323193157483.60176862745.39551080231.80614551583260.215822713560417.27192801610277257.7685688

1058675.562903382173.149610722871911.679897401180653.745115238665696.23323193157483.60176862745.39551080231.80614551583260.215822713560417.27192801610277257.7685688

8778318.006552786145409.862672919015282.219467735657160.374001245988498.078662893569499.66727251893.714551110377618.599803836489308.892165810679717.512094114747424.8831318

8778318.006552786145409.862672919015282.219467735657160.374001245988498.078662893569499.66727251893.714551110377618.599803836489308.892165810679717.512094114747424.8831318

9706243.8350775910427021.817024312184598.740759411799893.9170699639251.9978757310997861.37212569265.845605212511905.8210717803662.9858991610066106.29710235871654.6498387

9527749.3636365410427021.817024311862104.319234811799893.9170699639251.9978757310997861.37212569265.845605212511905.8210717803662.985899169734589.306458925871654.6498387

178494.471441051322494.421524609250941.796579293

80575.1940641297

2052568.62935686484028.86880709381304.370116525206246.91924099870365.7332278806.3112983921284907.356830411794364.93638424325790.238515665

2052568.62935686484028.86880709381304.370116525206246.91924099870365.7332278806.3112983921284907.356830411794364.93638424325790.238515665

6018795.782042974964054.00444258482401.612011394916010.421228975592562.026548217926191.8973472763.555873323221640.733005524011618.914564683838731.804337653415322.4179028

218409.370283519

1677274.0896413038123.132964912014303.992691712980541.376065491214886.7412090001.03069219867055.4237959321612925.17015187440772.6428

5704385.657960122561744.236684892639899.136046481722963.182074611268314.146938821025604.9121382762.525181131242714.819219832011706.401602731097184.762421291753855.62768899

314410.124082851725035.6781166132804379.3431178743.246462651343706.50354395685700.2441978925.91378569914447.7188825011128621.871764481220694.1474138

2192841.256592585245746.112379395904261.258630951603919.641920533080958.046258917962696.371411748460.6341931452389.173597923116413.232008381612790.688004276

1941600.069192584889394.570809235663552.376285841603919.641920533080958.046258917944422.8131748460.6341931452389.173597923116413.232008381612790.688004276

1941600.069192584889394.570809235663552.376285841603919.641920533080958.046258917944422.8131748460.6341931452389.173597923116413.232008381612790.688004276

251241.1874356351.541570158240708.88234510218273.55841

251241.1874356351.541570158240708.88234510218273.55841

10138720.049266212006283.601384510820423.231590611348305.735837212004095.50325556975260.3888321129809.63357648996289.7531470615995930.11815335740565.197365179919478.04220359

10138720.049266212006283.601384510820423.231590611348305.735837212004095.50325556975260.3888321129809.63357648996289.7531470615995930.11815335740565.197365179919478.04220359

10138720.049266212006283.601384510820423.231590611348305.735837212004095.50325556975260.3888321129809.63357648996289.7531470615995930.11815335740565.197365179919478.04220359

584452.444814458798809.5297933724126918.748122872146735.373079552358488.621518281757971.0011660322.316421722776308.655586831106416.04454294543749.2986689671584447.95624019

518176.456773572424637.047663615528185.540504711640317.8647246234.590751227

518176.456773572424637.047663615528185.540504711640317.8647246234.590751227

584452.444814458567140.0598435491892779.199526311635943.879811481422909.67591631932695.5521263431.438306771628490.49630152444168.050575575382278.228377444938752.780891698

522426.204018085567140.0598435491692584.51850631528171.011289221241003.45861122487320.70361086889.474568421628490.49630152163218.489584817382278.228377444541929.287437541

62026.2407963725200194.68102001107772.868522263181906.217305091445374.8484176541.963738358280949.560990758396823.493454157

231669.4699498231715963.0918229886154.4456044533407393.405097261184957.5843396890.878114949901583.568534076662247.993967366161471.070291524645695.175348489

231669.4699498231715963.0918229886154.4456044533407393.405097261184957.5843396890.878114949901583.568534076662247.993967366161471.070291524645695.175348489

4195736.691622312712842.321772715891850.627949843347117.056217184393233.365764042126372.5374428239.427723732607019.222785213342685.844450241745054.675603911745689.16164371

4195736.691622312712842.321772715891850.627949843347117.056217184393233.365764042126372.5374428239.427723732607019.222785213342685.844450241745054.675603911745689.16164371

4195736.691622312712842.321772715891850.627949843347117.056217184393233.365764042126372.5374428239.427723732607019.222785213342685.844450241745054.675603911745689.16164371

45372177.784558340693218.259637740059251.144929934612125.454602425228778.86977928185079.2834224213688.867713343377285.915770432027990.662569429714120.261374661237986.1656428

4017135.06014132738239.4092274356180612.02463803250068.3612115272188377.565542551235421.89922932450.42068729313243.3955512053.448932321197648.4759078041000417.62558159

4017135.06014132738239.4092274356180612.02463803250068.3612115272188377.565542551235421.89922932450.42068729313243.3955512053.448932321197648.4759078041000417.62558159

4017135.06014132738239.4092274356180612.02463803250068.3612115272188377.565542551235421.89922932450.42068729313243.3955512053.448932321197648.4759078041000417.62558159

6933032.786768534256857.740117527016194.344773845017068.943923694519656.779962263992234.369914018834.759625647243033.3111898110320111.213715818369577.546052112445716.0887026

5408177.193313332664012.884388413744979.49620292573222.188417683620510.96806226817928.622212010753.103068914557736.292986698957833.7059869915528911.90560962015553.69081566

5408177.193313332664012.884388413744979.49620292573222.188417683620510.96806226817928.622212010753.103068914557736.292986698957833.7059869915528911.90560962015553.69081566

1524855.59345521592844.855729113271214.848570942443846.755506899145.81193174305.74772008081.656556732685297.018203121362277.507728842840665.640442510430162.3978869

1524855.59345521592844.855729113271214.848570942443846.755506899145.81193174305.74772008081.656556732685297.018203121362277.507728842840665.640442510430162.3978869

1608964.634753493466481.466236583762516.594076915967102.859438946417314.889292082794400.89824824357.903229642219012.18723715571.789073772110608.577818651421551.58990563

1608964.634753493466481.466236583762516.594076915967102.859438946417314.889292082794400.89824824357.903229642219012.18723715571.789073772110608.577818651421551.58990563

1608964.634753493466481.466236583762516.594076915967102.859438946417314.889292082794400.89824824357.903229642219012.18723715571.789073772110608.577818651421551.58990563

564956.70019252342649.130378631789984.94532681741.950055771043254.993384582671789.0173557924.644584946353539.578363262057304.88693895903856.4150032794485549.38337061

564956.70019252342649.130378631789984.94532681741.950055771043254.993384582671789.0173557924.644584946353539.578363262057304.88693895903856.4150032794485549.38337061

377363.1921638446.91232835990007.7262080457.27535577375064.01791898098.5411191218.70913895254816.756827513961437.690145166

187593.5081925704202.218050278799977.2193601284.6747668190.975484581773690.4763557924.644584946353539.57836326866086.1778649039.6581757663524111.69322545

675285.179784081323041.26376703187332.612733801951500.9631886796.839722168796867.21507201139.206861793443977.69770404595083.891292264

675285.179784081323041.26376703187332.612733801951500.9631886796.839722168796867.21507201139.206861793443977.69770404595083.891292264

675285.179784081323041.26376703187332.612733801951500.9631886796.839722168796867.21507201139.206861793443977.69770404595083.891292264

26051756.604397627347246.823053814350562.932555214465885.60157686491251.3000379514929301.913624592781.238581428325899.512145311810423.83202385770458.0238271538406079.1042674

24091362.173486420304133.587609313608101.004654913535532.87240666060829.7897379514550378.527224023129.640063822577402.04514536261111.091972352040558.1403928827848305.1821021

2815229.39250439244428.402207755684387.587819572484018.962648763080958.046258918200578.15641786714.874549912456759.55921867116413.23200838111054187.0997279

15841902.31361184334096.522860233704516.305375212499466.52326612464276.1660117682295678.35212294599.99094340519675366.3509036651385.4772471491315147.81326447

1166655.156862561374745.376531111617986.16340747892344.013236866846406.086863841947314.6627953811.210367923726245.5489555101.5830445231196766.111074963191768.21770481

4267575.310507745350863.286010182601210.948052697659703.373254821669189.490603442106807.356988003.5642025622175790.145341642597864.47246201727378.79730953512287202.0514049

1960394.430911147043113.23544449742461.927900236930352.729170208430421.5103378923.3864569651.5985176045748497.4675549312.740051483729899.8834342810557773.9221653

1960394.430911147043113.23544449742461.927900236930352.729170208430421.5103378923.3864569651.5985176045748497.4675549312.740051483729899.8834342810557773.9221653

310096.8980569851642686.236910211865909.6354495692305.44678433616.18389119118019.58222347557834.5704564638179758.065612672

310096.8980569851642686.236910211865909.6354495692305.44678433616.18389119118019.58222347557834.5704564638179758.065612672

310096.8980569851642686.236910211865909.6354495692305.44678433616.18389119118019.58222347557834.5704564638179758.065612672

4947788.772291311313601.638608915001962.039638763208697.463793944122523.732902491949328.630146176759.504800993230739.277021762953674.155031661907061.739920612460439.91601712

3372995.68866546459066.133058372895526.529781322151265.340692121601329.012144992691988.546830393120404.44372176967872.8636078481537668.51788914461780.622139332

3372995.68866546459066.133058372895526.529781322151265.340692121601329.012144992691988.546830393120404.44372176967872.8636078481537668.51788914461780.622139332

1574793.08362586854535.505550542106435.509857451057432.123101822521194.72075751949328.630143484770.9579706110334.83331985801.29142381369393.2220314681998659.29387779

901347.33175682449018.757609068664376.625428903297435.711805936749240.158216181363265.019211980687.84826534110334.8333374012.58987218787772.8604600021393720.94192631

136189.888611863177205.26013114130.570704255440051.185649632

673445.751869037405516.7479414711305868.99581668759996.4112958891771954.562541321408858.35081504083.109705261611788.70155162167489.79086721164887.166301842

573258.046229494595003.890190873227399.4143031531104149.67597223359602.768934929423429.8932475825.0054503851129821.37442279658851.336853021397074.912388556743390.500892979

573258.046229494595003.890190873227399.4143031531104149.67597223359602.768934929423429.8932475825.0054503851129821.37442279658851.336853021397074.912388556743390.500892979

573258.046229494595003.890190873227399.4143031531104149.67597223359602.768934929423429.8932475825.0054503851129821.37442279658851.336853021397074.912388556743390.500892979

509418.4025565932075105.44216213410544.138326286812205.75362812911.154815991570223.76933526343.404786397251888.9625078853.8431046460582.932353042285191.261603538

509418.4025565932075105.44216213410544.138326286812205.75362812911.154815991570223.76933526343.404786397251888.9625078853.8431046460582.932353042285191.261603538

509418.4025565932075105.44216213410544.138326286812205.75362812911.154815991570223.76933526343.404786397251888.9625078853.8431046460582.932353042285191.261603538

509418.4025565932075105.44216213410544.138326286812205.75362812911.154815991570223.76933526343.404786397251888.9625078853.8431046460582.932353042285191.261603538

509418.4025565932075105.44216213410544.138326286812205.75362812911.154815991570223.76933526343.404786397251888.9625078853.8431046460582.932353042285191.261603538
